# Systematics of *Huicundomantis*, a new subgenus of *Pristimantis* (Anura, Strabomantidae) with extraordinary cryptic diversity and eleven new species

**DOI:** 10.3897/zookeys.868.26766

**Published:** 2019-08-01

**Authors:** Nadia B. Páez, Santiago R. Ron

**Affiliations:** 1 Museo de Zoología, Escuela de Biología, Pontificia Universidad Católica del Ecuador, Av. 12 de Octubre y Roca, Aptdo. 17-01-2184, Quito, Ecuador Pontificia Universidad Católica del Ecuador Quito Ecuador; 2 Department of Zoology, University of British Columbia, Vancouver, British Columbia, Canada V6T 1Z4 University of British Columbia Vancouver Canada

**Keywords:** Andes, cryptic diversity, integrative taxonomy, Neotropics, *Pristimantis phoxocephalus* species group, *P. cryptomelas* species group

## Abstract

*Pristimantis* is the most diverse genus of tetrapods comprising 532 described species. It contains a large number of morphologically cryptic species that are being discovered with the assistance of genetic evidence. We use molecular, morphological, bioacoustic, and environmental data to assess the phylogenetic relationships and determine the species within an Andean clade of *Pristimantis*, which is distributed from central Ecuador to northern Peru. We assign to this clade the name *Huicundomantis* and propose it as a subgenus. Our results show that *Huicundomantis* is composed of two large clades which we name as the *P. phoxocephalus* species group and the *P. cryptomelas* species group. *Huicundomantis* is composed of 28 species of which 12 have been described and 16 are new. We describe 11 of these undescribed species. The most effective characters to discriminate among species are DNA sequences, qualitative morphology, and advertisement calls. Morphometric and environmental characters are not very useful to define species limits. We clarify the identity of *P. riveti* and show that populations from southern Ecuador traditionally ascribed to *P. riveti* are a new species, *P. lutzae***sp. nov.** We also show that *P. prometeii* is a junior synonym of *P. hampatusami*. The current diversity and geographic distribution of *Huicundomantis* are consistent with a model of allopatric speciation. All species have a restricted distribution range (less than 4330 km^2^) and are assigned to the Red List categories Data Deficient or threatened with extinction. We provide new reasons to increase conservation efforts for these species and their habitat. Taking our results into account, *Pristimantis* species richness in Ecuador increases from 211 to 221 species, and the number of species endemic to Ecuador from 119 to 129.

## Abstract (es)

*Pristimantis* es el género más diverso de tetrápodos, contando con 532 especies descritas. Contiene un gran número de especies morfológicamente crípticas que están siendo descubiertas con el uso de evidencia genética. En el presente estudio usamos análisis integrativos, incluyendo información molecular, morfológica, bioacústica y ambiental para determinar el contenido de especies de un clado andino de *Pristimantis* que se distribuye desde el centro del Ecuador hasta el norte de Perú. Asignamos a este clado el nombre de *Huicundomantis* y lo proponemos con el rango de subgénero. Nuestros resultados indican que *Huicundomantis* está compuesto por dos grandes clados que nombramos como los grupos de especies *P. phoxocephalus* y *P. cryptomelas*. *Huicundomantis* contiene 28 especies de las cuales 12 están descritas y 16 son nuevas. En este estudio describimos 11 de las especies nuevas. Los caracteres más efectivos para discriminar entre especies de *Huicundomantis* son secuencias de ADN, morfología cualitativa y cantos de anuncio. Diferencias morfométricas y ambientales entre especies son de poca utilidad para delimitar especies. En este estudio también clarificamos la identidad de *P. riveti* y determinamos que poblaciones del sur del Ecuador, tradicionalmente consideradas *P. riveti*, corresponden a la nueva especie *P. lutzae***sp. nov.** Además, reportamos a *P. prometeii* como sinónimo junior de *P. hampatusami*. La diversidad y distribución geográfica de *Huicundomantis* son consistentes con un modelo de especiación alopatrica. Todas las especies tienen un rango de distribución restringido (menos de 4330 km^2^) y son asignadas a las categorías de Lista Roja de Datos Insuficientes o amenazadas de extinción. Nuestros resultados son un nuevo argumento para aumentar los esfuerzos de conservación de estas especies y su hábitat. Tomando en cuenta nuestros resultados, la riqueza de especies de *Pristimantis* en el Ecuador aumenta de 211 a 221 especies y su número de especies endémicas, de 119 a 129.

## Introduction

*Pristimantis* Jiménez de la Espada, 1870 is the most speciose genus of tetrapods with 532 described species (Frost 2019). It is distributed from Honduras in Central America to northern Argentina and southern Brazil, from the sea level to elevations above 4000 m a.s.l. (Frost 2019). In Ecuador, it comprises 34.3% of the anuran diversity with 204 species, of which 114 are endemic to the country (Ron et al. 2019).

*Pristimantis* taxonomy has been complex and labile because early studies were based almost exclusively on external morphology (e.g., Lynch and Duellman 1997; Lynch and Duellman 1980; Duellman and Lehr 2009), which was frequently insufficient to infer species limits and phylogenetic relationships. Recent studies have shown that species richness of *Pristimantis* is underestimated due to the existence of morphologically cryptic species (e.g., Elmer and Cannatella 2009; Padial and de la Riva 2009; Hutter and Guayasamin 2015; Ortega et al. 2015). The use of DNA sequences in combination with morphological and behavioral data has allowed objective species delimitation in a framework known as ‘integrative taxonomy’ (Will et al. 2005; Bickford et al. 2007; Padial et al. 2010). This approach has been increasingly applied to Neotropical amphibians resulting in the discovery of large numbers of cryptic species (e.g., Ron et al. 2006; Fouquet et al. 2007; Elmer and Cannatella 2009; Vieites et al. 2009; Funk et al. 2012; Caminer and Ron 2014; Ortega et al. 2015).

Unfortunately, relatively few groups of *Pristimantis* have been subject of systematic reviews based on genetic characters. Among the species that await such review are *Pristimantis phoxocephalus* (Lynch, 1979) and its close relatives *Pristimantis riveti* (Despax, 1911), *P. spinosus* (Lynch, 1979), and *Pristimantis versicolor* (Lynch, 1979). *Pristimantis phoxocephalus* was described from the Pacific versant of the Andes of central and southern Ecuador; its type locality is Pilaló in Cotopaxi Province (central Ecuador) at an elevation of 2340 m (Lynch 1979). Duellman and Wild (1993) provided two records from the Huancabamba Cordillera in northern Peru. Lynch and Duellman (1997) published a species account and hypothesized a close relationship with *P. eugeniae*, *P. nyctophylax*, and *P. subsigillatus*. They also suggested the inclusion of these species in the *P. lacrimosus* species group. Duellman and Pramuk (1999) commented on the existence of morphological differences between Ecuadorian and Peruvian populations. Duellman and Lehr (2009) provided a species account, noted additional morphological differences between Ecuadorian and Peruvian populations, and recommended the use of molecular characters to test the conspecificity of Ecuadorian and Peruvian populations. As currently defined, *P. phoxocephalus* has a wide distribution range, occurring in the Andean slopes from central Ecuador to northern Peru between 1800 and 3400 m a.s.l. (Duellman and Lehr 2009; Ron et al. 2019).

*Pristimantis riveti* was described from a single specimen collected in Ecuador, El Mirador (Despax 1911). This species name was not associated with any population until Lynch (1969) incorrectly identified populations of *P. curtipes* as “*P. riveti*” (Lynch 1979). Subsequently, Lynch (1979) provided a species account including a detailed description of morphological variation, comparisons with similar species, and natural history. The species account was based on specimens collected near Cuenca, Azuay Province, in southern Ecuador. Remarkably, Lynch characterized the distribution range of *P. riveti* as “both Andean Cordilleras and the connecting nudos at elevations of 2620–3420 m surrounding the Cuenca hoya in southern Ecuador”, a range that excludes the type locality. Subsequent references to the species (e.g., Almendáriz and Orcés 2004; Székely et al. 2016) relied on the morphological characterization provided by Lynch (1979) and ascribed the binomen to populations from Azuay Province and the adjacent Cañar and Loja Provinces. Heinicke et al. (2007) included a sample of “*P. riveti*” in their phylogeny. The sample was also from Azuay Province. Lynch and Duellman (1980) assigned *P. riveti* to the *P. unistrigatus* assemblage, and Hedges et al. (2008) later assigned it to the *P. unistrigatus* species group. Padial et al. (2014) left it unassigned.

*Pristimantis spinosus* was described by Lynch (1979) with the type locality “Sapote, Provincia Morona-Santiago, Ecuador, 2470 m”. Lynch (1979) hypothesized a close relationship with *P. nigrogriseus* and *P. cryptomelas*. Heinicke et al. (2007) included in their phylogeny a sample from Ecuador, Morona-Santiago, 10.6 km west of Plan de Milagro. Hedges et al. (2008) assigned it to the *Pristimantis unistrigatus* species group, and Padial et al. (2014) left it unassigned.

*Pristimantis versicolor* was described by Lynch (1979) from specimens collected at “15 km E Loja, 2800 m” in Loja Province, southern Ecuador. Duellman and Pramuk (1999) provided a species account and reported populations from Cordillera del Cóndor in northern Peru. Heinicke et al. (2007) included a sample of *P. versicolor* in their phylogeny which showed a close relationship with *P. riveti* and *P. phoxocephalus*. Lynch (1979) hypothesized a close relationship with *P. cajamarcensis* and *P. unistrigatus*. Lynch and Duellman (1980) assigned it to the *P. unistrigatus* assemblage, and Hedges et al. (2008) later assigned it to the *P. unistrigatus* species group. As with *P. riveti*, Padial et al. (2014) left it unassigned.

Our study aims to determine the content, identity, and phylogenetic relationships of species within *P. phoxocephalus* and closely related species. Our systematic review includes the description of 11 new species of which 10 belong to the *P. phoxocephalus* species group and one to the *P. cryptomelas* species group. Our taxonomic review is based on genetic, morphologic, bioacoustic, and environmental information.

## Materials and methods

### Species sampling

We focused our analysis in *P. phoxocephalus* and closely related species (Pyron and Wiens 2011; Padial et al. 2014). We expanded our study group to include morphologically similar species and species shown to be closely related to *P. phoxocephalus* based on an unpublished phylogeny of *Pristimantis* obtained by SRR as part of a large-scale review of Ecuadorian *Pristimantis*.

### Phylogenetic analyses

For the molecular phylogenetic analyses, we used sequences of 85 individuals ascribed to the species above and added sequences of 48 individuals as the outgroup (46 *Pristimantis* spp., *Craugastor talamancae*, *Epipedobates boulengeri*). Our final dataset consists of sequences of 133 individuals from 68 localities. Sequences of 117 of these individuals were newly generated and obtained from tissues deposited at the genome bank of the Zoology Museum, Pontificia Universidad Católica del Ecuador (QCAZ), representing 25 described and at least 22 undescribed species of *Pristimantis*. The remaining sequences, representing 16 species, were downloaded from GenBank (http://www.ncbi.nlm.nih.gov/genbank). Vouchers and GenBank accession numbers are shown in Table 1.

**Table 1.** Genbank accession numbers for DNA sequences used in the phylogenetic analyses.

Species	Voucher	16S	ND1	RAG1	GenSeq Nomenclature
* Craugastor talamancae *	QCAZ 30675	MK881414	MK881414	MK881322	genseq-3
* Epipedobates boulengeri *	QCAZ 42251	MK881437	NA	MK881337	genseq-4
* P. appendiculatus *	QCAZ 16365	MK881401	NA	MK881315	genseq-4
*P. atillo* sp. nov.	QCAZ 31946	MK881419	NA	MK881324	genseq-2
	QCAZ 40584	MK881435	NA	MK881335	genseq-2
	QCAZ 42485	MK881438	NA	MK881338	genseq-2
	QCAZ 42486	MK881439	NA	MK881339	genseq-2
	QCAZ 42488	MK881440	NA	MK881340	genseq-2
	QCAZ 42489	MK881441	NA	MK881341	genseq-2
	QCAZ 42492	MK881442	NA	MK881342	genseq-2
	QCAZ 42496	MK881443	NA	MK881343	genseq-2
	QCAZ 42498	MK881444	NA	MK881344	genseq-2
	QCAZ 42499	MK881445	NA	MK881345	genseq-2
	QCAZ 42500	MK881446	NA	MK881346	genseq-1
	QCAZ 42501	MK881447	NA	MK881347	genseq-2
	QCAZ 42503	MK881448	NA	MK881348	genseq-2
	QCAZ 42505	MK881449	NA	MK881349	genseq-2
	QCAZ 42506	MK881450	NA	MK881350	genseq-2
	QCAZ 42512	MK881451	NA	MK881351	genseq-2
	QCAZ 42543	MK881452	NA	MK881352	genseq-2
	QCAZ 42548	MK881453	NA	MK881353	genseq-2
* P. atratus *	QCAZ 45580	MK881471	MK881471	MK881364	genseq-4
	QCAZ 45645	MK881473	MK881473	MK881366	genseq-4
* P. bicantus *	QCAZ 31860	MK881417	NA	NA	genseq-4
	QCAZ 31986	MK881420	NA	MK881325	genseq-4
* P. cajamarcensis *	KU 217845	EF493663	NA	NA	genseq-4
	QCAZ 17142	MK881403	MK881403	MK881317	genseq-4
	QCAZ 24648	MK881405	NA	NA	genseq-4
	QCAZ 31474	MK881416	NA	NA	genseq-4
* P. ceuthospilus *	CORBIDI 4306	MK881397	MK881397	MK881311	genseq-4
*P. chomskyi* sp. nov.	QCAZ 45666	MK881476	MK881476	MK881369	genseq-2
	QCAZ 45669	MK881477	MK881477	MK881370	genseq-1
* P. cryptomelas *	QCAZ 45612	MK881472	MK881472	MK881365	genseq-4
	QCAZ 45660	MK881475	MK881475	MK881368	genseq-4
* P. curtipes *	QCAZ 17505	MK881404	MK881404	MK881318	genseq-4
	QCAZ 39568	MK881429	MK881429	NA	genseq-4
	QCAZ 39569	MK881430	MK881430	NA	genseq-4
* P. eremitus *	QCAZ 43392	MK881460	NA	NA	genseq-4
* P. gagliardoi *	QCAZ 42575	MK881456	NA	MK881355	genseq-4
	QCAZ 46738	MK881480	NA	MK881372	genseq-3
* P. glandulosus *	QCAZ 40153	MK881431	MK881431	MK881333	genseq-4
*P. gloria* sp. nov.	KU 218035	EF493348	NA	NA	genseq-4
	QCAZ 16448	MK881402	MK881402	MK881316	genseq-2
	QCAZ 31455	MK881415	NA	NA	genseq-2
	QCAZ 56463	MK881502	NA	NA	genseq-2
	QCAZ 57201	MK881503	NA	NA	genseq-1
* P. hampatusami *	QCAZ 58042	MK881504	MK881504	MK881387	genseq-3
*P. jimenezi* sp. nov.	QCAZ 45170	MK881466	MK881466	MK881360	genseq-1
	QCAZ 45178	MK881468	MK881468	MK881362	genseq-2
	QCAZ 46977	MK881481	MK881481	MK881373	genseq-2
	QCAZ 46978	MK881482	MK881482	MK881374	genseq-2
* P. kichwarum *	QCAZ 25766	JN991458	NA	JQ025196	genseq-4
* P. latidiscus *	QCAZ 17101	EF493354	NA	EF493440	genseq-4
	QCAZ 25852	JN991451	NA	JQ025188	genseq-4
* P. lividus *	QCAZ 54322	MK881497	MK881497	MK881382	genseq-4
*P. lutzae* sp. nov.	QCAZ 27471	MK881410	NA	NA	genseq-2
	QCAZ 27534	MK881411	NA	NA	genseq-2
	QCAZ 27597	MK881412	NA	NA	genseq-2
	QCAZ 32785	MK881421	MK881421	MK881326	genseq-2
	QCAZ 32786	MK881422	MK881422	MK881327	genseq-2
	QCAZ 32791	MK881424	MK881424	MK881329	genseq-2
	QCAZ 37546	MK881428	NA	NA	genseq-2
	QCAZ 47211	MK881487	NA	NA	genseq-2
	QCAZ 53728	MK881495	NA	NA	genseq-1
*P. multicolor* sp. nov.	QCAZ 47213	MK881488	NA	NA	genseq-1
	QCAZ 47214	MK881489	NA	NA	genseq-2
* P. muscosus *	QCAZ 54857	MK881501	MK881501	MK881386	genseq-4
*P. nangaritza* sp. nov.	QCAZ 41710	MK881436	MK881436	MK881336	genseq-1
* P. omeviridis *	QCAZ 10564	MK881398	NA	MK881312	genseq-4
* P. petrobardus *	KU 212293	EF493367	NA	NA	genseq-2
* P. philipi *	KU 217863	EF493672	NA	NA	genseq-3
	QCAZ 37528	MK881425	MK881425	MK881330	genseq-3
	QCAZ 37537	MK881426	MK881426	MK881331	genseq-3
	QCAZ 37542	MK881427	MK881427	MK881332	genseq-3
* P. phoxocephalus *	QCAZ 58463	MK881507	MK881507	MK881390	genseq-3
* P. pichincha *	QCAZ 11673	MK881399	MK881399	MK881313	genseq-4
* P. pycnodermis *	KU 218028	EF493680	NA	NA	genseq-4
	KU 218030	EF493683	NA	NA	genseq-4
* P. quaquaversus *	QCAZ 25676	JN991463	NA	JQ025201	genseq-4
* P. rubicundus *	QCAZ 26551	MK881407	MK881407	MK881320	genseq-4
* P. sobetes *	QCAZ 11737	MK881400	NA	MK881314	genseq-4
* P. spinosus *	KU 218052	EF493673	NA	NA	genseq-4
* P. sternothylax *	CORBIDI 13316	MK881393	MK881393	MK881308	genseq-4
*P. teslai* sp. nov.	QCAZ 46213	MK881478	NA	NA	genseq-1
* P. thymelensis *	QCAZ 16428	EF493516	NA	EF493442	genseq-4
	QCAZ 62801	MK881508	MK881508	MK881391	genseq-4
	QCAZ 62803	MK881509	MK881509	MK881392	genseq-4
* P. tinguichaca *	QCAZ 31945	MK881418	MK881418	MK881323	genseq-3
	QCAZ 40581	MK881432	NA	NA	genseq-3
	QCAZ 40582	MK881433	NA	MK881334	genseq-3
	QCAZ 42570	MK881454	NA	NA	genseq-3
	QCAZ 42574	MK881455	NA	MK881354	genseq-3
	QCAZ 42576	MK881457	NA	MK881356	genseq-3
	QCAZ 42578	MK881458	NA	MK881357	genseq-3
	QCAZ 54601	MK881498	NA	MK881383	genseq-3
	QCAZ 54602	MK881499	NA	MK881384	genseq-3
	QCAZ 54603	MK881500	NA	MK881385	genseq-3
*P. torresi* sp. nov.	QCAZ 47342	MK881490	NA	NA	genseq-1
	QCAZ 47397	MK881492	MK881492	MK881380	genseq-2
*P. totoroi* sp. nov.	KU 218025	EF493349	NA	NA	genseq-4
	QCAZ 25105	MK881406	MK881406	MK881319	genseq-1
	QCAZ 58425	MK881505	MK881505	MK881388	genseq-2
* P. unistrigatus *	KU 218057	EF493387	NA	EF493444	genseq-4
*P. verrucolatus* sp. nov.	QCAZ 45174	MK881467	MK881467	MK881361	genseq-2
	QCAZ 45195	MK881469	MK881469	MK881363	genseq-2
	QCAZ 46982	MK881483	MK881483	MK881375	genseq-1
	QCAZ 46992	MK881484	MK881484	MK881376	genseq-2
	QCAZ 46993	MK881485	MK881485	MK881377	genseq-2
* P. versicolor *	KU 218096	EF493389	NA	EF493431	genseq-4
	QCAZ 45650	MK881474	MK881474	MK881367	genseq-4
	QCAZ 46310	MK881479	MK881479	MK881371	genseq-4
* P. wiensi *	KU 219796	EF493668	NA	NA	genseq-2
* P. yumbo *	QCAZ 52241	MK881494	NA	MK881381	genseq-3
	QCAZ 58450	MK881506	MK881506	MK881389	genseq-4
*Pristimantis* sp. (CCS1)	QCAZ 32790	MK881423	MK881423	MK881328	genseq-4
*Pristimantis* sp. (CCS2)	QCAZ 45129	MK881462	MK881462	MK881358	genseq-4
	QCAZ 45155	MK881465	MK881465	MK881359	genseq-4
*Pristimantis* sp. (UCS1)	QCAZ 53999	MK881496	NA	NA	genseq-4
*Pristimantis* sp. (UCS2)	QCAZ 45029	MK881461	NA	NA	genseq-4
*Pristimantis* sp. (UCS3)	QCAZ 26642	MK881409	NA	NA	genseq-4
*Pristimantis* sp.	CORBIDI 14804	MK881394	MK881394	MK881309	genseq-4
	CORBIDI 14805	MK881395	NA	NA	genseq-4
	CORBIDI 2848	MK881396	MK881396	MK881310	genseq-4
	QCAZ 26586	MK881408	NA	MK881321	genseq-4
	QCAZ 29206	MK881413	NA	NA	genseq-4
	QCAZ 40583	MK881434	NA	NA	genseq-4
	QCAZ 43024	MK881459	NA	NA	genseq-4
	QCAZ 45131	MK881463	NA	NA	genseq-4
	QCAZ 45142	MK881464	NA	NA	genseq-4
	QCAZ 45210	MK881470	NA	NA	genseq-4
	QCAZ 47002	MK881486	MK881486	MK881378	genseq-4
	QCAZ 47353	MK881491	MK881491	MK881379	genseq-4
	QCAZ 50732	MK881493	NA	NA	genseq-4

DNA was extracted from liver or muscle tissue preserved in 95% ethanol or tissue storage buffer using a guanidine thiocyanate protocol (M. Fujita, unpublished) with some modifications. Primers used for the PCR amplification of the below-mentioned genes are listed in Suppl. material 1. Amplicons were sequenced by the Macrogen Sequencing Team (Macrogen Inc., Seoul, Korea).

The phylogenetic analyses were based on a 3199 bp dataset containing DNA sequences of the mitochondrial genes 16S (1318 bp, partial sequence), tRNA^Leu^ (90 bp), NADH dehydrogenase subunit 1 ND1 (961 bp), tRNA^Ile^ (73 bp), tRNA^Gln^ (71 bp), and tRNA^Met^ (31 bp), and the nuclear gene RAG1 (655 bp). The alignment of the sequences was performed in GeneiousPro v. 5.4.6 (GeneMatters Corp.) with the plug-in MAFFT (Katoh et al. 2002) and a posterior manual alignment with Mesquite v. 3.02 (Maddison and Maddison 2014). The aligned matrix is available at http://zenodo.org under https://doi.org/10.5281/zenodo.2555411. The best partitioning scheme and the best-fit model of molecular evolution for each partition were estimated with the software PartitionFinder v. 1.1.1 (Lanfear et al. 2012) under the greedy algorithm and the Bayesian information criterion (BIC).

Phylogenetic analyses were performed under two approaches, Bayesian Inference (BI) and Maximum-likelihood (ML). The Bayesian analysis was carried out with MrBayes v. 3.2.1 (Ronquist et al. 2012) in two independent searches for 3×10^7^ generations; each search had four Monte Carlo Markov Chains. The temperature parameter was set at 0.5. Trees were sampled every 1000 generations. We evaluated the convergence of the chains with software Tracer v. 1.6. (Rambaut et al. 2014) by inspecting plots of parameter values vs generation and confirming stationarity in their values. We also evaluated the Effective Sample Size and considered the search finished when all values were greater than 200. We discarded 25% of the 30000 trees as burn-in. The remaining trees were combined to obtain a 50% majority-rule consensus tree as well as the branch posterior probabilities (BPP).

For the ML analysis, we used Garli v. 2.0 (Zwickl 2006). We run ten replicate searches starting from stepwise trees and 10 from random trees. We finished the search after 100000 generations without topology improvements (genthreshfortopoterm = 100000); we used default values for the remaining parameters. Non-parametric bootstrap values (NPB) were obtained with 500 pseudoreplicates with the same settings of the random search, each with two independent searches. The 50% majority rule consensus for the bootstrap trees was obtained with Mesquite v. 2.75. We used the same settings to run two additional ML analyses, one exclusively for the nuclear gene and one for the mitochondrial genes. Throughout the text, we considered that a node has “strong support” when its bootstrap value is >70 and its Bayesian posterior probability is 0.95 or higher.

In order to identify candidate species, we calculated uncorrected *p* genetic distances for gene 16S (~1300 bp), within and between clades, with software MEGA v.7.0 (Kumar et al. 2016). See Integrative Analyses section for details.

### Morphological analyses

**Figure 1.**
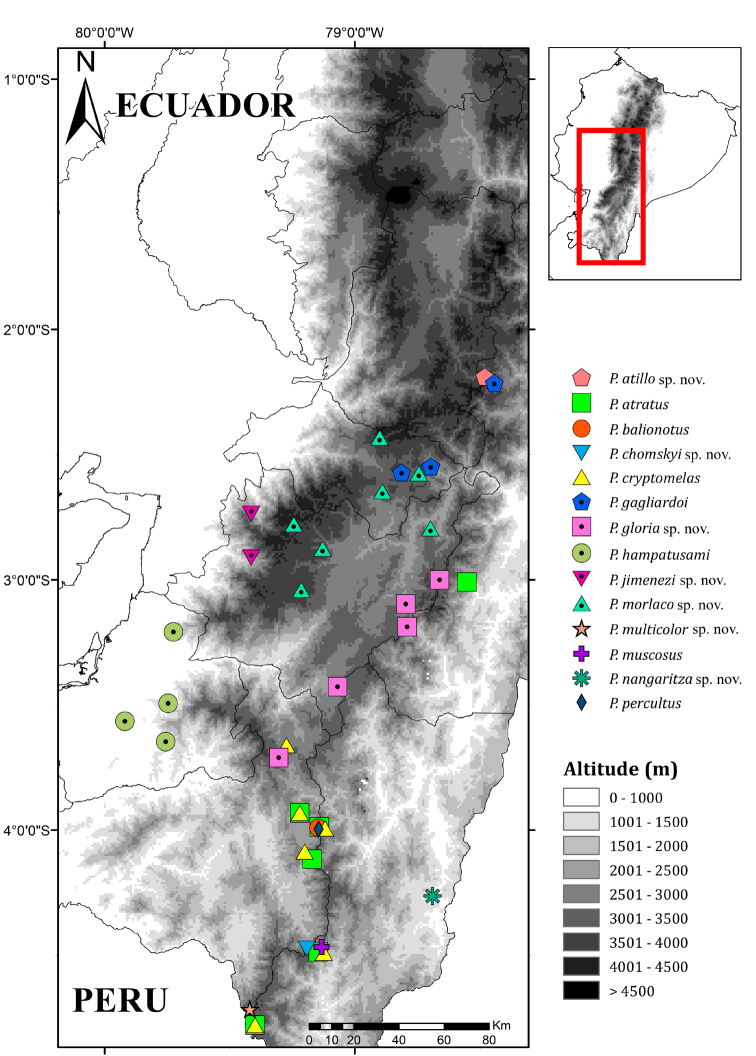
Distribution of *Pristimantis*, subgenus Huicundomantis. Records are based on specimens deposited at the Museum of Zoology, Pontificia Universidad Católica del Ecuador (QCAZ). Locality information is shown in Suppl. material 2.

**Figure 2.**
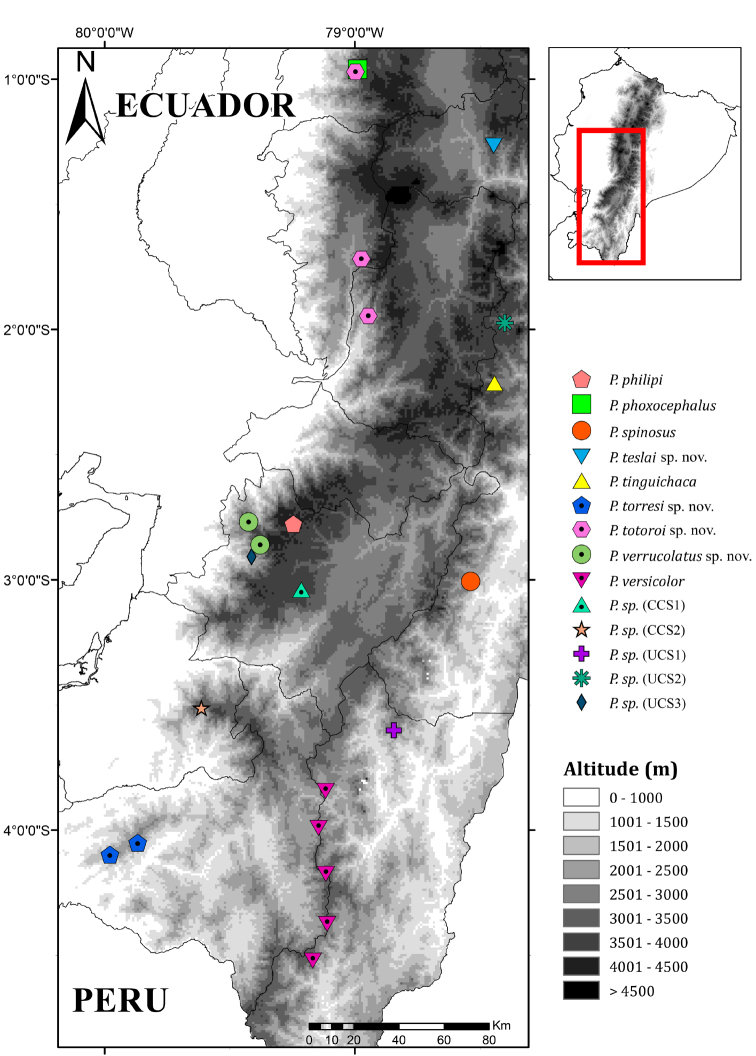
Distribution of *Pristimantis*, subgenus Huicundomantis. Records are based on specimens deposited at the Museum of Zoology, Pontificia Universidad Católica del Ecuador (QCAZ). Candidate species numbers correspond to those of the text and Figure 3. Locality information is shown in Suppl. material 2. Abbreviations: CCS = confirmed candidate species, UCS = unconfirmed candidate species.

Specimens deposited at the QCAZ collection were preserved in 10% formalin and stored in 70% ethanol. Photographs of preserved specimens were taken while these were submersed in ethanol to avoid reflections. We compared qualitative and quantitative morphological characters between clades. We used only adult specimens for these analyses but, when available, juveniles were considered to describe morphological variation. We examined 750 individuals from central and southern Ecuador ranging from 230 to 4200 m a.s.l. (Figs 1, 2), from collections of the Zoology Museum, Pontificia Universidad Católica del Ecuador (QCAZ), Ecuadorian Museum of Natural Sciences (MECN), Museum of Natural History, University of Kansas, USA (KU), Gustavo Orcés Museum of Natural History, Escuela Politécnica Nacional, Ecuador (MEPN), National Museum of Natural History, Smithsonian Institution, USA (USNM), and French Museum of Natural History of Paris (MNHNP); information for each individual is detailed in Suppl. material 2.

We determined sex and reproductive condition by looking for vocal sacs, vocal slits, and nuptial pads, and by gonadal inspection. For males, the presence of vocal sac, vocal slits, or nuptial pads was used as indicator of adulthood; when these characters were absent, we examined the gonads and categorized an individual as adult if the testes were swollen and enlarged. For females, we considered the presence of convoluted oviducts and large ovarian eggs as features of adulthood (Duellman and Lehr 2009).

Diagnosis and description of species follow Duellman and Lehr (2009) terminology. We use the term “distinct” for a character that is clearly visible, and “indistinct” for a character that is barely visible. When referring to the tympanum state, we use “prominent” to indicate that it protrudes from the skin surface; when referring to tubercles, we use “prominent” to imply they are conspicuous and elevated. Following Lynch and Duellman (1997), we qualify Toe V as slightly longer than Toe III when the distal end of Toe V does not reach the proximal edge of the distal subarticular tubercle on Toe IV when they are adpressed. Toe V is called longer than Toe III when its distal end reaches the proximal edge of the distal subarticular tubercle in Toe IV and does not extend to its distal edge. Toe V is much longer than Toe III if the distal end of Toe V reaches, or extends beyond, the distal edge of the distal subarticular tubercle on Toe IV. Abbreviations used for quantitative descriptive characters are: snout-vent length (SVL, distance from the tip snout to posterior margin of vent); head width (HW, greatest width of head); head length (HL, distance from the tip of snout to posterior angle of jaw articulation); eye diameter (ED, distance between anterior and posterior borders of eye); interorbital distance (IOD); upper eyelid width (EW); eye-nostril distance (EN); internarial distance (IND); tympanic diameter (TD, distance between external anterior and posterior margins of tympanic annulus); tympanum-eye distance (TED, distance between anterior margin of the tympanum to the closest point of the posterior margin of the eye); tibia length (TL, length of flexed leg from knee to heel); foot length (FL, distance from heel to tip of toe IV). All measurements were taken with a digital caliper with a precision of 0.01 mm.

For morphometric analyses, we measured 232 individuals of the candidate species and *P. phoxocephalus* and *P. versicolor*. We performed separate analyses for males and females. As they have been reported to be variable among *Pristimantis* species (Arroyo et al. 2005), we used SVL, HL, HW, TD, and ED measurements for morphometrics. To assess the degree of morphometric differentiation between candidate species, we performed a Discriminant Function Analysis (DFA) including the variables mentioned above without size correction. For pairwise comparisons, we used the Wilcoxon signed-rank test with corrected measurements. To remove the effect of co-varying body size, we made linear regressions between each variable and SVL. Statistical analyses were performed using software JMP v. 9.0.1 (SAS Institute Inc. 2010).

### Bioacoustic analyses

We analyzed recordings of eight individuals belonging to four candidate species of the clade under study, namely *Pristimantis jimenezi* sp. nov., *Pristimantis phoxocephalus*, *Pristimantis totoroi* sp. nov, and *Pristimantis verrucolatus* sp. nov. (see Results).

Recordings were obtained from the QCAZ audio library and are available in BIOWEB (http://bioweb.bio/faunaweb/amphibiaweb/). Recording temperatures ranged between 8.8 and 12 °C. Recordings of *P. phoxocephalus* were obtained at its type locality, Pilaló, Cotopaxi Province. We recorded one collected and two non-collected individuals from the same chorus. Their calls had similar properties; however, we did not include the advertisement call of the collected individual in the analysis to avoid accounting for effects of stress on the characteristics of the call given that it was recorded from a plastic bag after the individual’s capture. Advertisement calls for *P. jimenezi* sp. nov. were recorded from Azuay Province at two localities: at the border of Cajas National Park (QCAZ 45178) and Zadracay River (QCAZ 46977) (see Suppl. material 2 for details on localities); both individuals were sequenced. For *P. verrucolatus* sp. nov., we had recordings available for two individuals (QCAZ 46984, QCAZ 46981) from Shoupshe, Azuay Province. Finally, recordings for *P. totoroi* sp. nov. were obtained from Bosque Protector Cashca Totoras, Bolívar Province. We recorded a non-collected and a collected individual (QCAZ 25105), which was subsequently sequenced.

We analyzed calls with the software Raven Pro v. 1.3 (Cornell Lab of Ornithology 2003–2008) using a sampling rate of 44.1 kHz and a frequency resolution of 10.8–11.2 Hz. Temporal data was measured on oscillograms and frequency data on power spectrums (e.g., Caminer and Ron 2014). We randomly chose five advertisement calls from each recording and measured: (1) number of notes per call; (2) number of harmonics; (3) note duration; (4) inter-note interval; (5) peak time of the note (time from beginning of the note to peak energy); (6) dominant frequency; (7) frequency of the second harmonic; (8) initial and (9) final frequency of the note; and (10) frequency change in each note, estimated from the difference in frequency between the beginning and end of a note. Because of the low number of individuals recorded per species, we could not apply statistical comparisons. However, each species had a highly stereotyped call, qualitatively distinct from the others.

### Environmental analyses

We considered environmental differentiation as additional evidence to define species boundaries (e.g., Rissler and Apodaca 2007; Ortega et al. 2015). Accordingly, we analyzed differences in bioclimatic and vegetation-related variables between candidate species.

Values at each presence locality were extracted from raster maps using software ArcGIS 10.0 (ESRI). Following Menéndez-Guerrero and Graham (2013), we used eight bioclimatic variables with 30" resolution, downloaded from WorldClim (http://www.worldclim.org). The variables are: (1) annual mean temperature; (2) mean diurnal range; (3) temperature seasonality; (4) maximum temperature of warmest month; (5) minimum temperature of coldest month; (6) annual precipitation; and precipitation of (7) warmest and (8) coldest quarter. We also included three 30 m resolution layers of vegetation characteristics downloaded from Reverb (http://reverb.earthdata.nasa.gov): (1) leaf area index (LAI); (2) normalized difference vegetation index (NDVI); and (3) gross primary production (GPP). We used Principal Component Analysis (PCA) to examine environmental differentiation between candidate species using software JMP v. 9.0.1.

To avoid spatial autocorrelation, we reduced the number of localities using a buffer with a 5 km radius around each point; if two or more localities of the same species were at a distance <10 km from each other, we randomly chose one of them. We obtained a total of 62 localities for the analysis. As species limits in this clade are incorrectly defined, we did not include geographic records from the literature. Locality data are available in Suppl. material 2. A distribution map is presented in Figures 1, 2.

For the description of the habitat and distribution of the species, we assigned natural regions according to Ron et al. (2019); we estimated the Extent of Occurrence and Area of Occupancy of each species with the R package *red* (Cardoso 2017).

### Integrative analyses

To determine species limits, we examined the covariation of independent sets of characters under the framework of integrative taxonomy (Dayrat 2005; Vieites et al. 2009). Our datasets consisted of genetic, morphological, bioacoustic, and environmental characters. We assumed that covariation between independent sets of characters is an indication of evolutionary independence between lineages (Vieites et al. 2009).

Based on the correspondence between morphological and genetic divergence, we set a 0.02 threshold of uncorrected *p* distances for the gene 16S to identify candidate species. We chose a lower threshold than that proposed by Fouquet et al. (2007) for amphibians (0.03) because there is evidence indicating that sister species in *Pristimantis* (e.g., Ortega et al. 2015; Padial and de la Riva 2009) and other Neotropical frogs (e.g., Caminer and Ron 2014; Jungfer et al. 2013; Coloma et al. 2012; Funk et al. 2012) often differ by genetic distances <0.03. Candidate species categories follow Vieites et al. (2009). Each candidate species was categorized as: (1) unconfirmed candidate species (UCS) when genetic distance was >0.02 and there were no additional character sets available; (2) confirmed candidate species (CCS) when genetic distance was >0.02 and there was covariation between genetic and morphological, bioacoustic and/or environmental characters; (3) deep conspecific linage (DCL) when genetic distance was >0.02 and there was not covariation between genetic and morphological, bioacoustics, and environmental characters. We followed the species concept proposed by de Queiroz (2007) that defines a species as a separately evolving metapopulation lineage.

## Results

### Phylogeny and genetic variation

The best partition scheme for the concatenated matrix consisted of the following five partitions and models: 16S, ND1 1^st^ position (GTR+I+G); ND1 2^nd^ position (HKY+I+G); ND1 3^rd^ position (TrN+I+G); RAG1 3^rd^ position (K80+G); RAG1 1^st^ and 2^nd^ position (K81uf+I+G).

**Figure 3.**
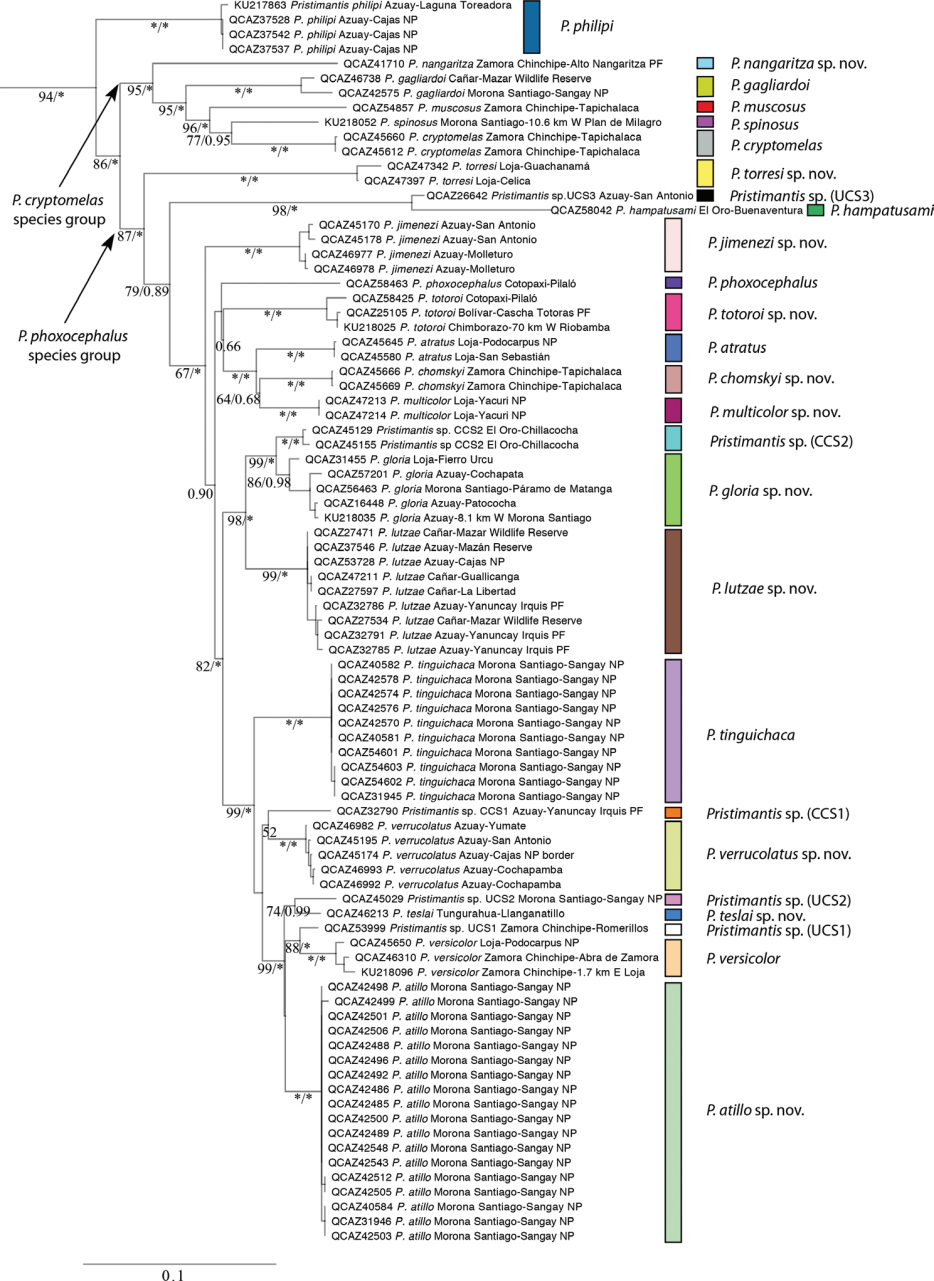
Phylogenetic relationships of *Pristimantis*, sugenus *Huicundomantis*. Maximum likelihood tree for genes 16S, ND1 and RAG1. Bootstrap values (%) followed by Bayesian posterior probabilities are shown under the corresponding branches. Asterisks indicate support values of 100 (bootstrap) or 1 (posterior probabilities); missing values indicate values below 50 (bootstrap) or 0.5 (posterior probability). The collection number, identification, province and locality of the samples are shown next to each terminal; all samples are from Ecuador. Outgroup is not shown. Abbreviations: CCS = confirmed candidate species, NP = national park, PF = protected forest, UCS = unconfirmed candidate species.

Tree topologies under ML and Bayesian inference were similar except for the order of divergence events of *P. tinguichaca*, *P.* sp. CCS1, and *P. atillo* sp. nov. (see Integrative results). The best ML tree based on nuclear and mitochondrial genes is shown in Figure 3; the consensus tree of the Bayesian analysis is available through zenodo.org (http://doi.org/10.5281/zenodo.2556418). We found a strongly supported clade (94 NPB, 1.0 BBP) that has *P. philipi* diverging basally and sister to a clade composed of two large subclades with strong support (Fig. 3 and Suppl. material 3). We are referring to these clades as the *P. phoxocephalus* and *P. cryptomelas* species groups. Figure 3 shows the species limits identified in the integrative analyses (see below). The topology of the ML tree based on mitochondrial genes was congruent with the one based on the complete dataset for branches with strong support (>75% NBP) (Suppl. material 4). The tree based on the nuclear gene (Suppl. material 5) has low resolution and low branch support.

Uncorrected *p*-genetic distances are summarized in Tables 2, 3. Average interspecific genetic distances (16S) for the ingroup range from 2.0 to 12.9% (1.8 to 5.9% between individuals of sister species). *Pristimantis gloria* sp. nov. was the species with the highest divergence between two populations; the population from Loja Province presented up to 1.3% of genetic distance with populations from Azuay Province (average intraspecific distance: 0.7%). We found 26 clades (i.e., candidate species) with genetic distances >2.0%. Ten of these clades are formerly described species: *P. atratus* (Lynch, 1979), *P. cryptomelas* (Lynch, 1979), *P. gagliardoi* Bustamante & Mendelson, 2008, *P. prometeii*Székely et al., 2016, *P. muscosus* (Duellman & Pramuk, 1999), *P. philipi*, *P. phoxocephalus*, *P. spinosus*, *P. tinguichaca*Brito et al., 2016, and *P. versicolor*; the remaining 16 clades are candidate species to be confirmed with the following analyses.

**Table 2.** Genetic divergence (Gene 16S) between species of the *Pristimantis phoxocephalus* species group. Mean uncorrected *p* distances (%) between groups are shown under the diagonal, standard error estimates above the diagonal. Mean uncorrected *p* distance within each clade and its standard error are shown on the diagonal. Number of samples is given in brackets after the name of each clade. Standard error estimates were obtained by a bootstrap procedure in MEGA 7.0.

		1	2	3	4	5	6	7	8	9	10	11	12	13	14	15	16	17	18	19	20
**1**	*P. atillo* (18)	0	0.9	0.9	0.7	0.9	1.0	0.8	0.9	1.0	0.6	0.8	1.0	0.9	0.8	0.7	0.8	0.8	0.5	0.7	0.9
**2**	*P. atratus* (2)	7.3	0	0.7	0.8	1.0	0.7	0.8	0.8	0.8	1.0	1.0	0.9	0.7	0.6	0.8	0.7	0.7	0.9	1.0	1.0
**3**	*P. chomskyi* (2)	7.1	5.4	0.2±0.1	0.7	1.0	0.7	0.7	0.8	0.8	1.0	1.0	0.9	0.7	0.7	0.7	0.7	0.7	0.9	1.0	1.0
**4**	*P. gloria* (5)	5.1	5.8	6.3	0.7±0.2	0.9	0.7	0.6	0.8	0.8	0.8	0.9	0.9	0.7	0.7	0.7	0.7	0.4	0.8	0.9	0.9
**5**	*P. hampatusami* (1)	7.0	11.1	10.9	8.7	NA	1.0	0.9	1.0	1.0	1.0	0.9	1.0	1.0	0.9	0.9	1.0	0.9	1.0	1.1	0.7
**6**	*P. jimenezi* (4)	7.7	6.8	7.8	5.8	10.7	0.5±0.2	0.7	1.0	0.9	1.0	1.0	0.9	0.8	0.6	0.7	0.7	0.6	1.0	1.1	1.0
**7**	*P. lutzae* (9)	5.0	5.7	5.6	2.9	7.5	5.3	0.2±0.1	0.9	0.8	0.8	0.8	0.9	0.7	0.6	0.7	0.7	0.6	0.8	0.9	0.9
**8**	*P. multicolor* (2)	6.7	4.4	4.9	5.5	8.9	6.7	5.5	0	0.9	1.0	1.0	1.0	0.9	0.9	1.0	0.9	0.8	1.0	1.0	1.0
**9**	*P. phoxocephalus* (1)	7.8	5.8	6.7	5.4	11.1	6.8	5.8	6.3	NA	1.1	1.0	1.0	0.7	0.8	0.9	0.8	0.9	1.0	1.1	1.0
**10**	*P. teslai* (1)	2.6	7.6	7.4	5.9	7.6	7.9	5.2	7.1	8.0	NA	0.9	1.0	1.0	0.8	0.8	0.8	0.9	0.6	0.6	1.0
**11**	*P. tinguichaca* (10)	4.7	7.8	7.7	6.1	7.0	7.8	5.3	7.5	7.7	5.7	0	1.0	0.9	0.8	0.8	0.8	0.9	0.8	0.9	0.9
**12**	*P. torresi* (2)	8.7	8.7	9.5	7.3	11.1	9.1	7.5	8.3	8.6	9.1	8.6	0.9±0.4	0.9	0.9	0.8	0.9	0.8	1.0	1.1	1.1
**13**	*P. totoroi* (3)	7.0	6.0	6.8	6.0	11.1	6.9	5.5	6.4	5.9	7.4	7.2	8.0	1±0.2	0.7	0.7	0.8	0.7	0.9	0.9	0.9
**14**	*P. verrucolatus* (5)	4.6	5.4	6.6	4.8	9.7	5.6	4.3	6.0	6.1	4.9	4.9	8.6	5.7	0.2±0.1	0.6	0.5	0.6	0.8	0.8	0.9
**15**	*P. versicolor* (3)	3.9	7.2	7.4	6.0	10.2	7.5	5.6	7.9	8.1	4.4	5.8	8.7	7.1	4.7	0.38±0.1	0.6	0.6	0.7	0.8	1.0
**16**	*Pristimantis* CCS1 (1)	4.4	6.2	7.2	5.3	10.4	6.4	5.1	6.9	6.8	4.7	4.6	8.6	6.1	3.2	5.0	NA	0.6	0.8	0.9	0.9
**17**	*Pristimantis* CCS2 (2)	5.4	5.8	6.0	2.0	7.5	5.8	2.9	5.4	6.0	6.4	5.8	7.6	5.9	4.5	5.7	5.1	0.19±0.1	0.8	0.9	0.9
**18**	*Pristimantis* UCS1 (1)	2.5	7.0	7.2	5.4	7.4	7.5	5.1	6.5	7.3	3.0	5.6	8.6	7.4	4.3	2.9	4.8	5.8	NA	0.6	0.9
**19**	*Pristimantis* UCS2 (1)	3.2	7.7	7.3	6.3	8.0	8.4	5.9	6.9	8.1	2.5	5.8	9.9	7.4	4.9	4.3	4.8	6.6	2.8	NA	1.0
**20**	*Pristimantis* UCS3 (1)	6.9	8.1	7.4	6.6	3.5	7.3	6.2	8.0	7.7	7.1	6.5	8.5	7.7	6.9	7.5	6.7	6.8	7.2	7.7	NA

**Table 3.** Genetic divergence (16S) between species of the *Pristimantis cryptomelas* species group. Mean uncorrected *p* distances (%) between groups are shown under the diagonal, standard error estimates above the diagonal. Mean uncorrected *p* distance within each clade and its standard error are shown on the diagonal. Number of samples is given in brackets after the name of each clade. Standard error estimates were obtained by a bootstrap procedure in MEGA 7.0.

		1	2	3	4	5
**1**	*P. cryptomelas* (2)	0	1.1	0.8	0.9	0.6
**2**	*P. gagliardoi* (2)	8.8	0.9±0.4	1.1	1.1	1.0
**3**	*P. muscosus* (1)	7.6	8.6	NA	1.0	0.7
**4**	*P. nangaritza* (1)	8.1	8.3	9.8	NA	0.9
**5**	*P. spinosus* (1)	5.9	7.6	7.4	8.1	NA

### Morphological analyses

**Figure 4.**
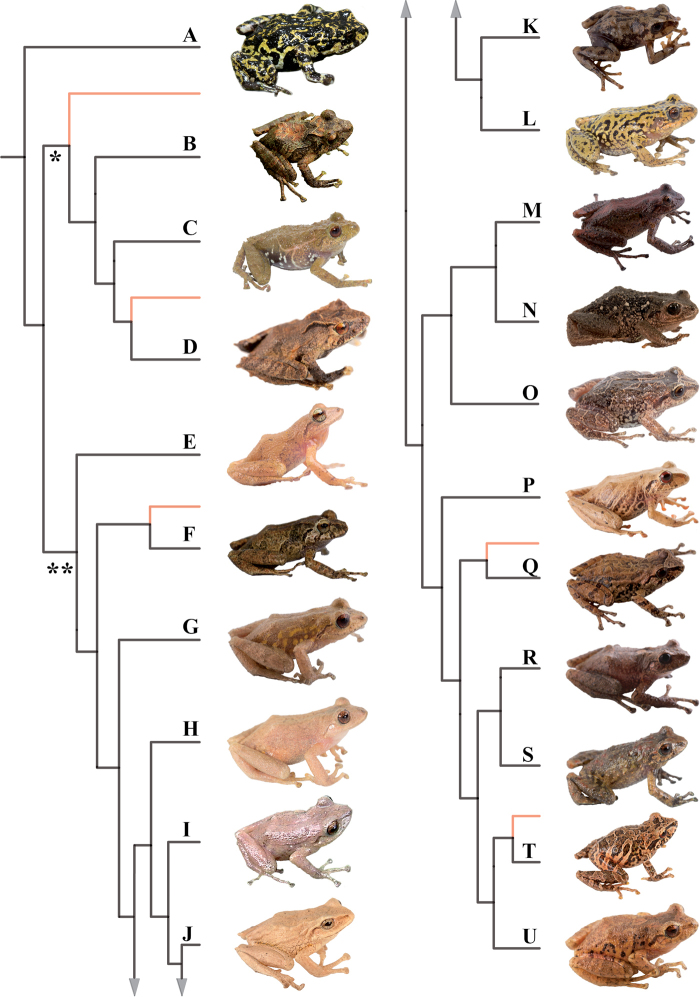
Morphological variation within *Pristimantis*, subgenus Huicundomantis. **A***Pristimantis philipi*. Pristimantis cryptomelas group (*): **B***Pristimantis gagliardoi***C***Pristimantis muscosus***D***Pristimantis cryptomelas. Pristimantis phoxocephalus* group (**): **E***Pristimantis torresi* sp. nov. **F***Pristimantis hampatusami***G***Pristimantis jimenezi* sp. nov. **H***Pristimantis phoxocephalus***I***Pristimantis totoroi* sp. nov. **J***Pristimantis atratus***K***Pristimantis chomskyi* sp. nov. **L***Pristimantis multicolor* sp. nov. **M***Pristimantis* sp. (CCS2) **N***Pristimantis gloria* sp. nov. **O***Pristimantis lutzae* sp. nov. **P***Pristimantis tinguichaca***Q***Pristimantis verrucolatus* sp. nov. **R***Pristimantis* sp. (UCS1) **S***Pristimantis teslai* sp. nov. **T***Pristimantis versicolor***U***Pristimantis atillo* sp. nov. Red branches are for clades without available photographs of live individuals. Not shown at the same scale.

Morphological variation is shown in Figure 4. The ingroup shares the absence of dorsolateral folds (except for *P. atratus*), presence of a tympanic annulus and membrane (except for *P. philipi*), dentigerous processes of vomers evident, first finger shorter than the second, presence of lateral fringes on fingers and toes, basal webbing on toes (except for *P. philipi* and *P. prometeii*), and small to prominent tubercles on heel. Additionally, all the species of the *P. cryptomelas* group bear postocular folds and prominent tubercles on eyelids and heels. Because postocular folds and prominent tubercles on eyelids and heels are absent in the *P. phoxocephalus* group and *P. philipi*, both characters appear to be synapomorphies for the *P. cryptomelas* group. Meanwhile, species of the *Pristimantis phoxocephalus* group have a keel or a papilla at the tip of the snout. The papilla is absent in *P. philipi* and all species of the *P. cryptomelas* group, except for *P. cryptomelas* which has papilla, suggesting that the presence of a papilla is a synapomorphy for the *P. phoxocephalus* group with an independent origin in *P. cryptomelas*. Traits that differ the most between species include snout shape, presence or absence of middorsal, lateral and postocular folds, dorsal skin texture, shape and width of discs, and coloration of groins, hidden surfaces of thighs, and iris. Character states for qualitative morphological traits are shown in Table 4.

**Table 4.** Qualitative morphological traits of *Pristimantis*, subgenus Huicundomantis. Coloration corresponds to live individuals unless otherwise noticed. + is for present; – is for absent.

	Dorsal texture	Cranial crests	Postocular/scapular fold/ridge	Middorsal fold	Tubercles row head	Dorsolateral folds	Lateral fold	Snout shape (dorsal; lateral)	Discs (expansion, shape)	Basal webbing	Iris coloration	Groin coloration
*P. atillo* sp. nov.	Shagreen with or without scattered small subconical tubercles	–	–	+/–	+	–	+/–	Acuminate; protruding; with a fleshy keel	Expanded to broadly expanded, rounded to elliptical	+	Copper with a faint medial horizontal darker streak	Bright orange, surrounded or not by yellow spots
* P. atratus *	Shagreen	Low	–	+/–	+/–	+	+	Subacuminate; rounded; with or without a papilla	Broadly expanded, rounded to elliptical	+	Pale yellow to pale bronze, thin brown streak	Black with white or yellow spots
* P. balionotus *	Tuberculate	–	–	–	–	–	+/–	Subacuminate; rounded; with a papilla	Slightly expanded, elliptical	–	Bronze with a red medial streak	In preservative, pale rusty brown with or without faint brown marbling
*P. chomskyi* sp. nov.	Shagreen	–	–	–	–	–	–	Subacuminate; rounded; with or without a papilla	Expanded, rounded to elliptical	+	Orange with a faint reddish brown streak	Dark chocolate brown with or without small cream flecks
*P. gloria* sp. nov.	Tuberculate to warty	–	–	+	–	–	–	Subacuminate; rounded; with or without a papilla	Expanded, truncate to elliptical	+	Light silver to cream with wide black reticulations and a red streak	Pinkish to purplish brown with irregular cream to light brown flecks or spots
* P. hampatusami *	Shagreen with scattered tubercles	–	Low, W-shaped	+	+	–	+	Subacuminate; rounded; with or without a papilla	Broadly expanded, elliptical to truncate	–	Golden to bronze with a red medial horizontal streak	Reddish brown with yellow spots
*P. jimenezi* sp. nov.	Shagreen with or without scattered small subconical tubercles	–	–	+	+	–	+	Acuminate; protruding; with a fleshy keel	Expanded to broadly expanded, elliptical to truncate	+	Copper with red medial streak to completely red	Pinkish, purplish or dark brown with small light brown to yellow spots
*P. lutzae* sp. nov.	Shagreen to tuberculate	–	–	+/–	+/–	–	+/–	Round to subacuminate, with or without a papilla	Expanded, elliptical to truncate	+	Golden to creamy brown with a reddish brown medial streak	Pinkish to reddish brown, suffused or not with orange, with cream or light brown spots
*P. multicolor* sp. nov.	Shagreen to warty	–	–	–	+/–	–	–	Subacuminate; rounded; with or without a papilla	Expanded to broadly expanded, elliptical to truncate	+	Copper to creamy yellow with or without a red to dark brown streak	Cream, orange, brown, or black with or without cream to yellow flecks or spots
* P. phoxocephalus *	Shagreen with scattered small tubercles	–	–	+	+	–	+/–	Acuminate; protruding; with a fleshy keel	Broadly expanded, rounded to elliptical	+	Golden with wide black reticulations	In preservative, brown with cream reticulation or spots
* P. percultus *	Tuberculate	Low	–	–	–	–	–	Subacuminate; acutely rounded; with a keel	Expanded, elliptical	+	Copper with or without a faint red medial streak	Yellow with black reticulations
*P. teslai* sp. nov.	Tuberculate with prominent and rounded tubercles	–	–	–	+	–	–	Acuminate; protruding; with a fleshy keel	Expanded to broadly expanded, elliptical to truncate	+	Copper	Dark brown with yellow irregular blotches
* P. tinguichaca *	Smooth	–	–	+	+	–	+	Subacuminate; rounded; with or without a papilla	Broadly expanded, rounded	+	Red with a dark medial streak	Reddish to dark brown
*P. torresi* sp. nov.	Shagreen	–	–	+	+/–	–	+	Acuminate; protruding; with a fleshy keel	Broadly expanded, elliptical to truncate	+	Golden to beige with red to reddish brown streak	Light purplish brown to brown with or without yellow spots
*P. totoroi* sp. nov.	Shagreen with or without scattered small tubercles	–	–	+	+	–	+	Acuminate; protruding; with a fleshy keel	Broadly expanded, rounded to elliptical	+	Golden with a medial horizontal red streak	In preservative, brown with or without pale spots
*P. verrucolatus* sp. nov.	Shagreen with or without scattered small tubercles	–	–	+/–	+/–	–	+	Acuminate; rounded; with a fleshy keel	Broadly expanded, elliptical to truncate	+	Coppery brown	Reddish brown with small light brown, orangey brown or yellow spots
* P. versicolor *	Shagreen to tuberculate	–	–	+/–	–	–	–	Subacuminate; rounded; with or without a papilla	Expanded, rounded to elliptical	+	Bronze-white with a reddish brown streak	Brown to black with cream to reddish pink flecks or spots
* P. philipi *	Tuberculate with low tubercles and warts	–	W-shaped ridge	+/–	+	–	–	Rounded	Slightly expanded, truncate	–	Grayish bronze	Black with or without white or yellow streaks
* P. cryptomelas *	Shagreen	–	Prominent,) (-shaped	+/–	+	–	+	Subacuminate; rounded; with or without a papilla	Broadly expanded, rounded to elliptical	+	Red, orange or cream with a red or orange streak	Black with or without yellow or white spots
* P. gagliardoi *	Shagreen with scattered tubercles	–	Prominent, W-shaped	+/–	+	–	+	Rounded	Broadly expanded, elliptical to truncate	+	Bronze with or without a brown medial horizontal streak	Pink to orange with or without brown blotches
* P. muscosus *	Smooth to shagreen	–	Low,) (-shaped	–	+	–	+	Rounded	Broadly expanded, elliptical to truncate	+	Reddish brown	Dark brown with orange, yellow or white spots
*P. nangaritza* sp. nov.	Finely tuberculate	–	Low,) (-shaped	+/–	+	–	+/–	Subacuminate; rounded	Broadly expanded, rounded to elliptical	+	Unknown	In preservative, light brown with or without pale flecks
* P. spinosus *	Finely tuberculate	Low	Low,) (-shaped	–	+	–	+	Subacuminate; rounded to truncate	Broadly expanded, rounded	+	Unknown	Black with big white spots

**Figure 5.**
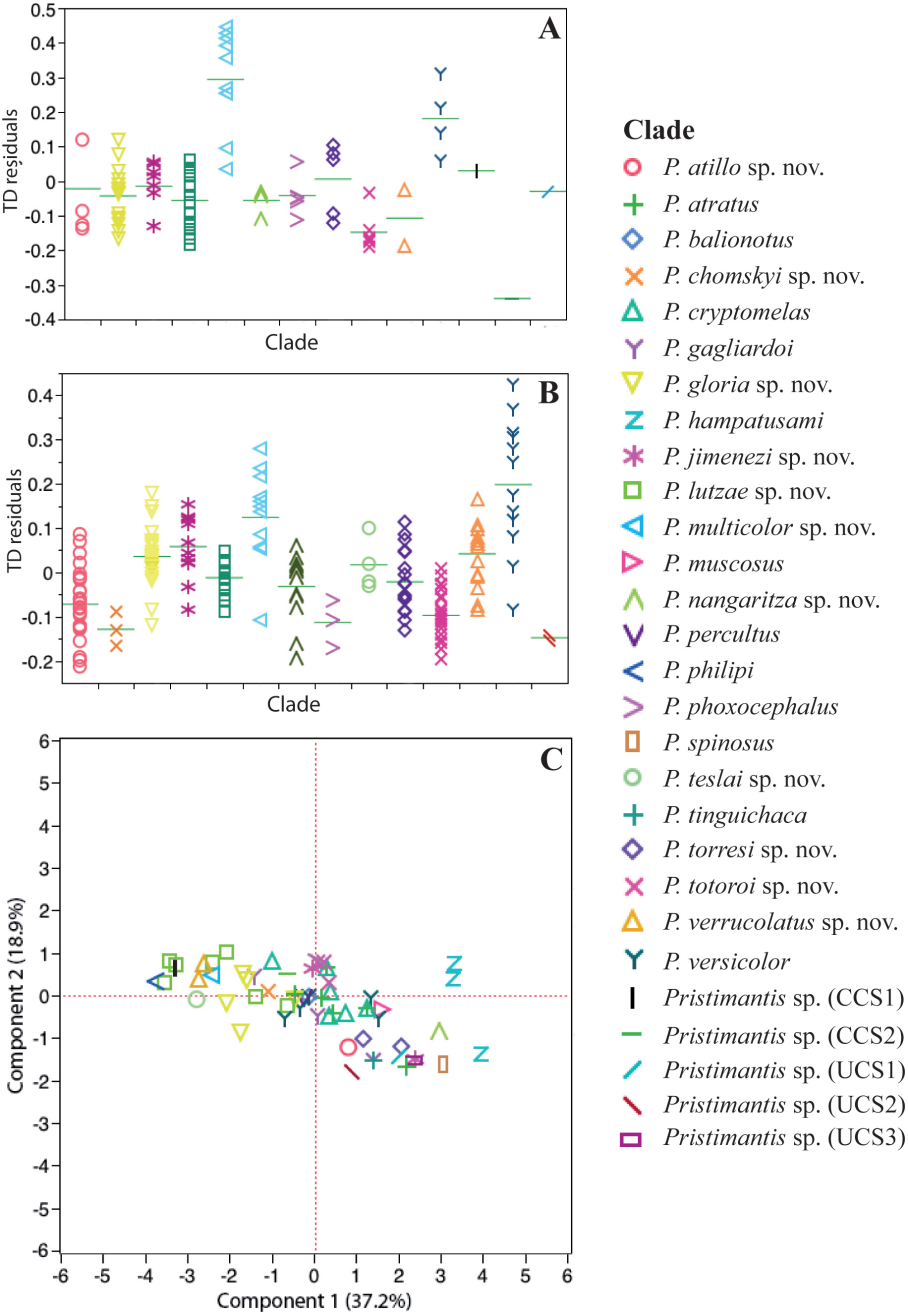
Morphological and environmental comparisons among species of *Pristimantis*, sugenus *Huicundomantis*. Intra and interspecific variation in tympanum size, respective to body, in **A** females and **B** males. Green horizontal lines represent the mean **C** Principal components from analysis of eleven environmental variables. See Table 7 for character loadings on each component.

Ranges of quantitative variables widely overlap among species (Fig. 5A, B). However, several species pairs differ significantly from each other according to one or more variables (see Fig. 5A, C for an example); these results will be mentioned when useful to discriminate similar species. The DFA classification assigned the correct species in 77.1% of the specimens in females (64 out of 83), and 68.7% in males (126 out of 182). Although the DFA classification was not perfect, it shows some morphometric differentiation among species. Summarized information of morphometric measurements is shown in Table 5.

**Table 5.** Descriptive statistics for morphometric variables of *Pristimantis*, subgenus Huicundomantis. Mean ± SD followed by the range of the measurements are given in each cell. ‘n’ is for the number of samples. Numbers in brackets after the species name refer to the bibliographic source of the data: (1) Lynch 1979, (2) Bustamante and Mendelson 2008, (3) Yánez-Muñoz et al. 2016, (4) Duellman and Pramuk 1999, (5) Lynch and Duellman 1995, (6) Brito et al. 2016; otherwise the source is this study. Abbreviations are: SVL = snout-vent length; HL = head length; HW = head width; TD = tympanum diameter; ED = eye diameter. All measurements are in mm.

Females					
Clade	SVL	HL	HW	TD	ED
*P. atillo* sp. nov. *n* = 5	31.3 ± 2.3; 29.4–35.3	11.0 ± 0.5; 10.3–11.7	11.5 ± 0.7; 10.9–12.7	1.7 ± 0.2; 1.5–1.8	3.2 ± 0.1; 3.1–3.3
*P. atratus* (1) *n* = 10	27.4 ± 2.1; 24.9–29.2	–	–	–	–
*P. balionotus* (1) *n* = 7	28.1 ± 0.6; 27.1–29.1	–	–	–	–
*P. cryptomelas* (1) *n* = 1	38.6	–	–	–	–
*P. gagliardoi* (2) *n* = 5	30.6 ± 3.1; 26.8–33.6	10.7 ± 0.6; 9.8–11.4	12.2 ± 1.0; 10.7–13.3	1.3 ± 0.2; 1.0–1.5	3.8 ± 0.3; 3.5–4.1
*P. gloria* sp. nov. *n* = 15	30.1 ± 3.0; 26.7–35.8	11.0 ± 0.7; 10.1–12.4	11.3 ± 1.1; 9.8–13.5	1.6 ± 0.1; 1.4–1.8	3.2 ± 0.2; 2.8–3.6
*P. hampatusami* (3) *n* = 11	30.6 ± 2.1; 25.9–34.1	–	–	–	–
*P. jimenezi* sp. nov. *n* = 9	35.0 ± 2.1; 31.1–37.4	12.0 ± 0.5; 11.0–12.6	13.1 ± 0.6; 13.0–13.9	1.9 ± 0.1; 1.7–2.1	3.7 ± 0.2; 3.5–4.0
*P. lutzae* sp. nov. *n* = 15	31.4 ± 1.3; 29.7–33.9	11.3 ± 0.4; 10.7–12.1	12.2 ± 0.5; 11.4–12.9	1.7 ± 0.1; 1.5–1.8	3.4 ± 0.2; 3.1–3.8
*P. multicolor* sp. nov. *n* = 10	35.3 ± 3.5; 29.4–40.5	13.4 ± 1.1; 11.6–15.1	14.4 ± 1.3; 11.9–16.1	2.2 ± 0.2; 1.8–2.5	4.0 ± 0.3; 3.6–4.4
*P. muscosus* (4) *n* = 4	37.8; 29.6–46.1	–	–	–	–
*P. nangaritza* sp. nov. *n* = 4	29.1 ± 2.7; 25.9–32.4	11.5 ± 0.8; 10.6–12.2	11.2 ± 1.0; 10.1–12.1	1.5 ± 0.1; 1.4–1.7	3.7 ± 0.3; 3.4–4.0
*P. percultus* (1) *n* = 1	38.2	–	–	–	–
*P. phillipi* (5) *n* = 6	31.2 ± 1.1; 26.5–33.7	–	–	–	–
*P. phoxocephalusn* = 5	36.9 ± 2.2; 34.2–39.9	12.9 ± 0.9; 11.5–13.8	13.1 ± 1.2; 11.3–14.2	1.9 ± 0.1; 1.8–2.1	3.8 ± 0.3; 3.5–4.1
*Pristimantis* CCS1 *n* = 1	37.0	12.5	13.8	2.0	3.5
*Pristimantis* CCS2 *n* = 1	38.3	13	14.4	1.7	3.5
*Pristimantis* UCS1 *n* = 1	31.2	12.4	12.4	1.7	3.8
*P. spinosus* (1) *n* = 29	31.8 ± 1.62; 28.3–34.5	–	–	–	–
*P. tinguichaca* (6) *n* = 9	29.7 ± 1.5; 28.1–31.7	10.6 ± 0.4; 10.1–11.0	11.0 ± 0.5; 10.1–11.5	1.4 ± 0.1; 1.3–1.7	3.3 ± 0.3; 2.8–3.7
*P. torresi* sp. nov. *n* = 5	34.7 ± 3.7; 30.1–39.5	12.2 ± 1.3 10.4–13.8	13.1 ± 1.2 11.5–14.6	1.9 ± 0.2 1.8–2.2	3.6 ± 0.3 3.3–3.9
*P. totoroi* sp. nov. *n* = 7	33.0 ± 0.9; 31.9–34.3	12.1 ± 0.4; 11.5–12.6	12.3 ± 0.3; 11.8–12.6	1.6 ± 0.1; 1.6–1.8	3.3 ± 0.2; 3.1–3.6
*P. verrucolatus* sp. nov. *n* = 2	43.6 ± 4.5; 40.4–46.8	15.0 ± 1.1; 14.2–15.8	16.6 ± 1.7; 15.3–17.8	2.2 ± 0.1; 2.1–2.3	4.2 ± 0.0; 4.2–4.3
*P. versicolorn* = 4	27.8 ± 3.5; 24.9–32.4	10.7 ± 1.6; 9.0–12.4	10.5 ± 1.0; 9.5–11.4	1.7 ± 0.3; 1.4–2.1	3.2 ± 0.4; 2.9–3.7
**Males**					
*P. atillo* sp. nov. *n* = 29	24.7 ± 2.5; 17.7–28.1	8.7 ± 0.7; 6.4–9.7	8.8 ± 0.9; 5.9–10.0	1.2 ± 0.1; 0.8–1.4	2.7 ± 0.3; 2.0–3.0
*P. atratus* (1) *n* = 19	21.7 ± 3.71; 17.4–24.0	–	–	–	–
*P. balionotus* (1) *n* = 2	20.0 ± 0.2; 21.8–22.2	–	–	–	–
*P. chomskyi* sp. nov. *n* = 3	28.0 ± 4.2; 24.0–32.4	9.7 ± 1.2; 8.8–11.1	10.6 ± 1.6; 9.4–12.4	1.3 ± 0.2; 1.1–1.5	3.3 ± 0.4; 2.9–3.6
*P. cryptomelas* (1) *n* = 4	29.2; 28.2–30.3	–	–	–	–
*P. gagliardoi* (2) *n* = 5	22.2 ± 2.0; 19.1–24.3	7.9 ± 0.9; 6.8–9.1	9.0 ± 0.9; 7.7–10.2	0.8 ± 0.0; 0.7–0.8	2.8 ± 0.2; 2.4–3.0
*P. gloria* sp. nov. *n* = 24	21.7 ± 2.3; 16.8–24.7	8.6 ± 0.7; 6.7–9.4	8.4 ± 0.8; 6.5–9.5	1.2 ± 0.1; 0.9–1.4	2.6 ± 0.2; 2.1–2.9
*P. hampatusami* (3) *n* = 28	21.0 ± 1.8; 17.0–24.9	–	–	–	–
*P. jimenezi* sp. nov. *n* = 12	25.5 ± 1.6; 21.8–27.1	8.9 ± 0.6; 7.8–9.6	9.1 ± 0.6; 7.8–9.7	1.4 ± 0.1; 1.3–1.5	3.06 ± 0.24; 2.56–3.37
*P. lutzae* sp. nov. *n* = 14	24.6 ± 1.7; 21.4–27.0	8.7 ± 0.5; 7.9–9.4	9.3 ± 0.7; 8.2–10.4	1.3 ± 0.1; 1.1–1.4	2.77 ± 0.19 2.53–3.26
*P. multicolor* sp. nov. *n* = 12	26.2 ± 3.5; 19.7–29.7	9.6 ± 1.0; 8.0–11.0	10.1 ± 1.2; 8.0–11.5	1.5 ± 1.2; 1.1–1.8	3.18 ± 0.49; 2.45–3.82
*P. nangaritza* sp. nov. *n* = 13	18.8 ± 1.1; 17.4–20.8	7.6 ± 0.6; 7.1–8.8	7.2 ± 0.5; 6.6–8.3	1.0 ± 0.1; 0.8–1.2	2.69 ± 0.17 2.49–3.08
*P. percultus* (1) *n* = 1	29.8	–	–	–	–
*P. phillipi* (5) *n* = 10	23.0 ± 0.4; 21.1–25.1	–	–	–	–
*P. phoxocephalusn* = 3	24.7 ± 3.2; 20.8–27.9	9.0 ± 1.36; 7.4–10.6	8.7 ± 1.5; 7.0–10.4	1.2 ± 0.2; 1.0–1.3	2.66 ± 0.25 2.36–2.90
*Pristimantis* UCS2 *n* = 2	20.5 ± 1.7; 19.3–21.7	7.6 ± 0.7; 7.1–8.1	7.3 ± 0.4; 7.0–7.6	1.0 ± 0.1; 0.9–1.0	2.43 ± 0.23 2.29–2.62
*P. spinosus* (1) *n* = 34	20.1 ± 1.8; 16.1–25.0	–	–	–	–
*P. teslai* sp. nov. *n* = 4	25.2 ± 1.8; 23.4–27.3	8.9 ± 0.4; 8.4–9.2	9.0 ± 0.6; 8.2–9.5	1.3 ± 0.1; 1.2–1.5	2.89 ± 0.14 2.76–3.07
*P. tinguichaca* (6) *n* = 11	23.4 ± 1.1; 21.2–24.7	8.7 ± 0.3; 8.2–9.2	8.6 ± 0.5; 7.9–9.4	1.3 ± 0.2; 1.1–1.6	2.8 ± 0.4 2.3–3.3
*P. torresi* sp. nov. *n* = 18	25.9 ± 2.1; 23.3–30.0	9.2 ± 0.6; 8.4–10.5	9.3 ± 0.7; 8.4–10.5	1.3 ± 0.1; 1.2–1.6	2.98 ± 0.23 2.64–3.47
*P. totoroi* sp. nov. *n* = 21	26.8 ± 2.0; 23.2–29.4	9.6 ± 0.6; 8.5–10.5	9.5 ± 0.6; 8.4–10.3	1.3 ± 0.1; 1.1–1.4	2.79 ± 0.25 2.27–3.25
*P. verrucolatus* sp. nov. *n* = 15	29.4 ± 2.7; 25.1–34.5	9.7 ± 0.7; 8.5–10.7	10.5 ± 1.0; 8.5–12.1	1.5 ± 0.1; 1.3–1.8	3.21 ± 0.24 2.79–3.64
*P. versicolorn* = 12	20.5 ± 1.6; 18.1–23.3	8.2 ± 0.5; 7.3–8.9	7.6 ± 0.6; 6.7–8.6	1.3 ± 0.2; 1.0–1.6	2.84 ± 0.32 2.32–3.36

### Bioacoustic analyses

**Figure 6.**
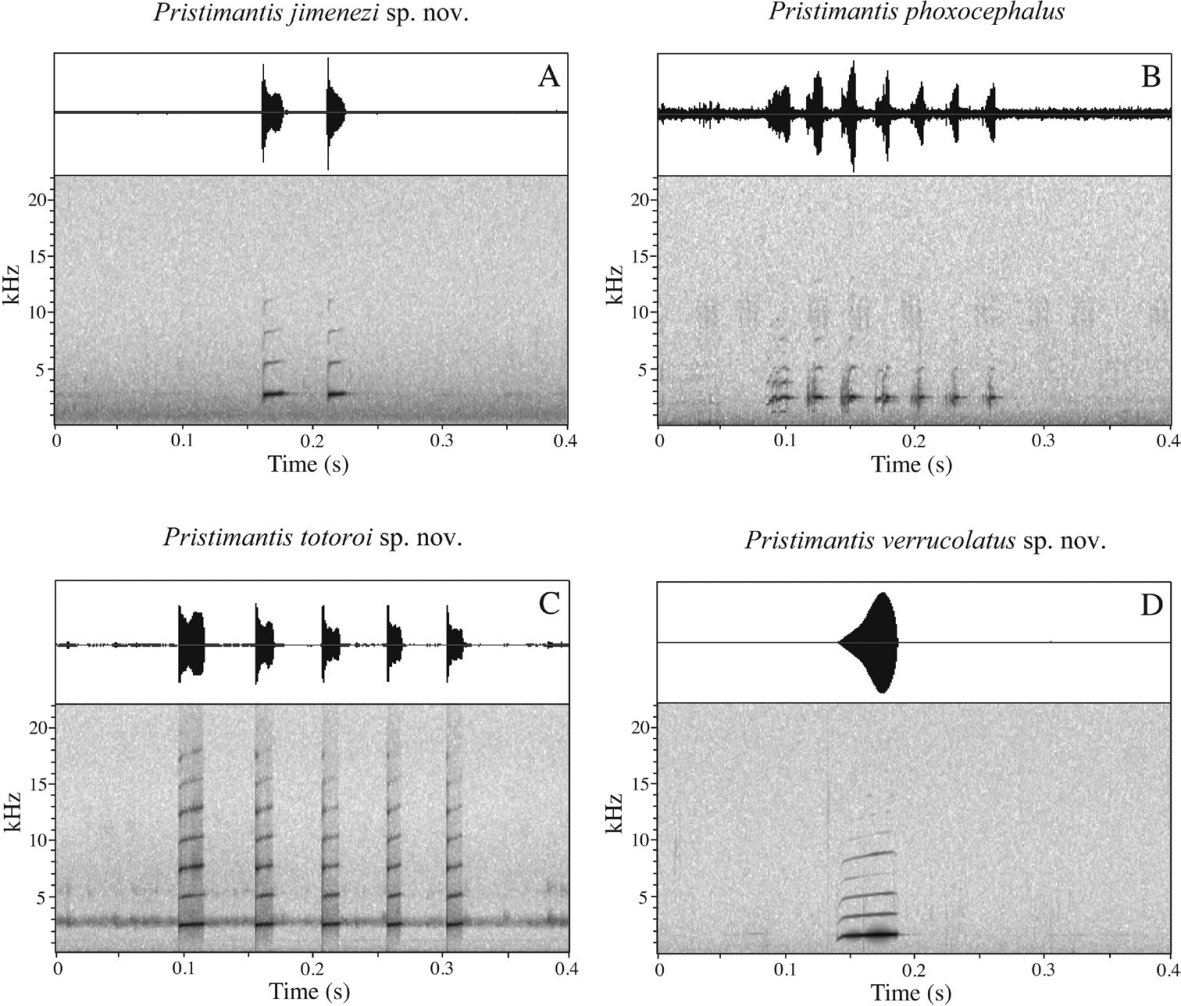
Comparison of advertisement calls of *Pristimantis jimenezi* sp. nov., *P. phoxocephalus*, *P. totoroi* sp. nov. and *P. verrucolatus* sp. nov. **A***Pristimantis jimenezi*: QCAZ 46977 **B***Pristimantis phoxocephalus*: non-collected individual from Pilaló (type locality). **C***Pristimantis totoroi*: non-collected individual from Cashca Totoras (type locality) **D***Pristimantis verrucolatus*: QCAZ 46981. Oscillograms are shown above spectrograms. X axis for time (s); Y axis for frequency (kHz) in spectrograms.

Bioacoustic data are summarized in Table 6. The available advertisement calls of *P. phoxocephalus* and three candidate species have a similar structure: one to several whistles (Fig. 6). Calls from Cashca Totoras (Bolívar Province, Ecuador) were distinct from those from Pilaló (Cotopaxi Province, Ecuador) even though both populations had historically been ascribed to *P. phoxocephalus*. The clade from high altitudes of Azuay Province shows, by far, the longest duration of the note, the lowest dominant frequency, and the highest variation between the initial and final frequency of the note. The advertisement call of the clade from lower altitudes of Azuay shows the highest frequency of the first and second harmonic. *Pristimantis phoxocephalus* shows the shortest interval between notes. All analyzed species are separated by large genetic distances (>5.6%). They also have unequivocally distinct advertisement calls (Fig. 6).

**Table 6.** Descriptive statistics for bioacoustic variables of *Pristimantis jimenezi* sp. nov., *P. phoxocephalus*, *P. totoroi* sp. nov. and *P. verrucolatus* sp. nov. Mean ± SD and range in brackets are given in each cell. The number of samples is given in brackets after species name.

	*P. jimenezi* (2)	*P. phoxocephalus* (2)	*P. totoroi* (2)	*P. verrucolatus* (2)
Notes per call	1–2	3–9	2–6	1
No. harmonics	4	4–5	3–7	4–7
Note duration (s)	0.182 ± 0.035	0.154 ± 0.030	0.125 ± 0.038	0.433 ± 0.037
	(0.157–0.206)	(0.133–0.175)	(0.098–0.152)	(0.407–0.459)
Interval between notes (s)	0.297 ± 0.088	0.135 ± 0.035	0.339 ± 0.038	NA
	(0.234–0.360)	(0.110–0.159)	(0.312–0.366)	
Peak time (s)	0.091 ± 0.018	0.077 ± 0.014	0.063 ± 0.019	0.216 ± 0.018
	(0.080–0.103)	(0.067–0.087)	(0.049–0.076)	(0.204–0.230)
Dominant frequency (Hz)	2894.58 ± 167.49	2578.12 ± 0	2569.65 ± 21.21	2114.08 ± 265.17
	(2776.15–3013.01)		(2512.22–2627.07)	(1926.58–2301.58)
Initial frequency (Hz)	2789.89 ± 232.02	2452.15 ± 87.01	2550.96 ± 148.20	1837.5 ± 212.13
	(2625–2953.13)	(2390.63–2513.67)	(2446.16–2655.75)	(1687.5–1987.5)
Final frequency (Hz)	2976.56 ± 232.02	2586.91 ± 12.43	2619.88 ± 50.73	2231.25 ± 238.65
	(2812.5–3140.63)	(2578.13–2595.70)	(2584–2655.75)	(2062.5–2400)
Frequency change (Hz)	187.5 ± 0	134.77 ± 99.44	68.92 ± 97.47	393.75 ± 26.52
		(64.45–205.08)	(0–137.84)	(375–412.5)
2^nd^ harmonic frequency (Hz)	5373.79 ± 412.25	4532.23 ± 62.15	4714.43 ± 453.59	4171.89 ± 596.64
	(5446.29–6029.3)	(4488.28–4576.17)	(4393.69–5035.16)	(3750–4593.78)

### Environmental analyses

In the PCA, the eleven environmental variables were reduced to four principal components (PCs) with an eigenvalue >1. Together they accounted for 81.58% of the variation. The highest loadings for each PC were: temperature-associated variables for PC I, vegetation-associated variables and precipitation of the coldest quarter for PC II, precipitation-associated variables for PC III, and NDVI and temperature seasonality for PC IV. Percentage of explained variance and variable loadings for each PC are shown in Table 7.

Candidate species widely overlap in environmental space (Fig. 5C). However, we found differences between clades occurring in areas with the lowest altitudes and warmest temperatures ((*P. hampatusami* = *P. prometeii*), *P. nangaritza* sp. nov., *P. spinosus*) relative to those occurring in paramo (*P. gloria* sp. nov., *P. lutzae* sp. nov., *P. multicolor* sp. nov., *P. philipi*, *P. teslai* sp. nov., *P. verrucolatus* sp. nov., *Pristimantis* sp. CCS1).

**Table 7.** Character loadings, eigenvalues and percentage of explained variance for Principal Components (PC) I–IV. The analysis was based in eleven environmental variables extracted from presence localities of 26 species of *Pristimantis*, subgenus Huicundomantis. Bold figures indicate highest loadings.

Variable	PCI	PCII	PCIII	PCIV
Annual mean temperature	**0.956**	0.014	-0.139	0.153
Mean diurnal temperature range	**0.680**	-0.365	-0.026	-0.383
Temperature seasonality	-0.257	0.016	0.465	**0.642**
Max temperature of warmest month	**0.966**	-0.046	-0.122	0.121
Min temperature of coldest month	**0.930**	0.050	-0.153	0.201
Annual precipitation	0.382	-0.395	**0.777**	-0.101
Precipitation of warmest quarter	0.219	0.156	**0.709**	0.234
Precipitation of coldest quarter	0.428	-**0.605**	0.210	-0.268
Gross primary production	0.377	**0.839**	0.128	-0.185
Leaf area index	0.469	**0.797**	0.146	-0.125
Normalized difference vegetation index	0.339	-0.229	-0.378	**0.611**
Eigenvalue	4.096	2.074	1.606	1.198
Cumulative variance (%)	37.23	56.09	70.69	81.58

### Integrative analyses

By combining evidence from the abovementioned character sets, we conclude that our study group is composed of 28 candidate species. Of them, 25 are confirmed candidate species and 3 are unconfirmed. Of the CCS, 12 have available names and the 13 remaining are new species. We describe 11 of them below. We did not describe two CCS because we did not have enough specimens to characterize species variation. Because of their similar morphology to the examined species, and after a thorough bibliographic analysis, we propose *P. balionotus* (Lynch, 1979) and *P. percultus* (Lynch, 1979) as part of our clade of study.

In brief, our ingroup is composed of 27 species divided in two species groups and *P. philipi*. The *P. phoxocephalus* species group comprises 22 species: *P. atillo* sp. nov., *P. atratus*, *P. chomskyi* sp. nov., *P. gloria* sp. nov., *P. hampatusami*, *P. jimenezi* sp. nov., *P. lutzae* sp. nov., *P. multicolor* sp. nov., *P. phoxocephalus*, *P. teslai* sp. nov., *P. tinguichaca*, *P. torresi* sp. nov., *P. totoroi* sp. nov., *P. versicolor*, *P. verrucolatus* sp. nov., *Pristimantis* sp. (CCS1), and *Pristimantis* sp. (CCS2); and three unconfirmed species: *Pristimantis* sp. (UCS1), *Pristimantis* sp. (UCS2), and *Pristimantis* sp. (UCS3). The *P. cryptomelas* species group comprises *P. cryptomelas*, *P. gagliardoi*, *P. muscosus*, *P. nangaritza* sp. nov., and *P. spinosus*. We present the evidence used to define the limits of each species in the following section.

We identified that the recently described *P. hampatusami*Yánez-Muñoz et al., 2016 and *P. prometeii*Székely et al., 2016 belong to the same species. We compared specimens of both species from the QCAZ and INABIO collections. Our comparisons showed that both species are morphologically indistinguishable. The type locality for both species is the same further suggesting that both represent a single species. With this evidence, we propose *P. prometeii* as a junior synonym for *P. hampatusami* given the principle of priority.

We found a misidentification of two Genbank sequences with accession numbers KU217863 and KU218025. GenBank identifications are *P. cryophilius* and *P. phoxocephalus*, respectively. They actually are *P. philipi* (KU217863) and *P. totoroi* sp. nov. (KU218025). Updated identifications of sequences used in this study are shown in Table 1.

In the following section, we present the descriptions of the new species and new taxa identified in this study. We also include an updated diagnosis of *P. phoxocephalus* sensu stricto, as diagnoses made in its description and later revisions (e.g., Duellman and Pramuk 1993; Lynch and Duellman 1997) included information of non-conspecific populations.

## Systematic accounts

### Huicundomantis subgenus nov.

Taxon classificationAnimaliaAnuraStrabomantidae

9097e14e-f8a4-53d6-a517-956a8045dae7

http://zoobank.org/A18720A1-FB71-4342-B0B2-8C9C8F06B5F4

#### Type species.

*Pristimantis phoxocephalus* (Lynch, 1979).

#### Definition.

**Figure 7.**
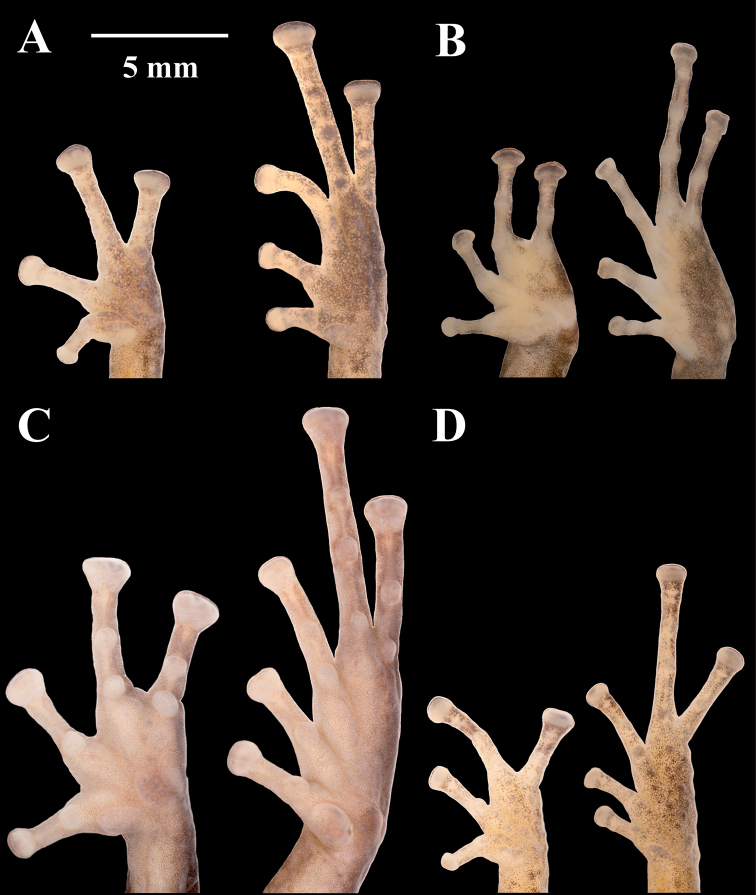
Palmar and plantar surfaces of *Pristimantis atillo* sp. nov., *Pristimantis chomskyi* sp. nov., *Pristimantis gloria* sp. nov., and *Pristimantis jimenezi* sp. nov. Photographs of left hand and foot of the holotypes **A***Pristimantis atillo* sp. nov.: QCAZ 42500, male **B***Pristimantis chomskyi* sp. nov.: QCAZ 47515, male **C***Pristimantis gloria* sp. nov.: QCAZ 57201, female **D***Pristimantis jimenezi* sp. nov.: QCAZ 45170, male. All specimens are shown at the same scale.

**Figure 8.**
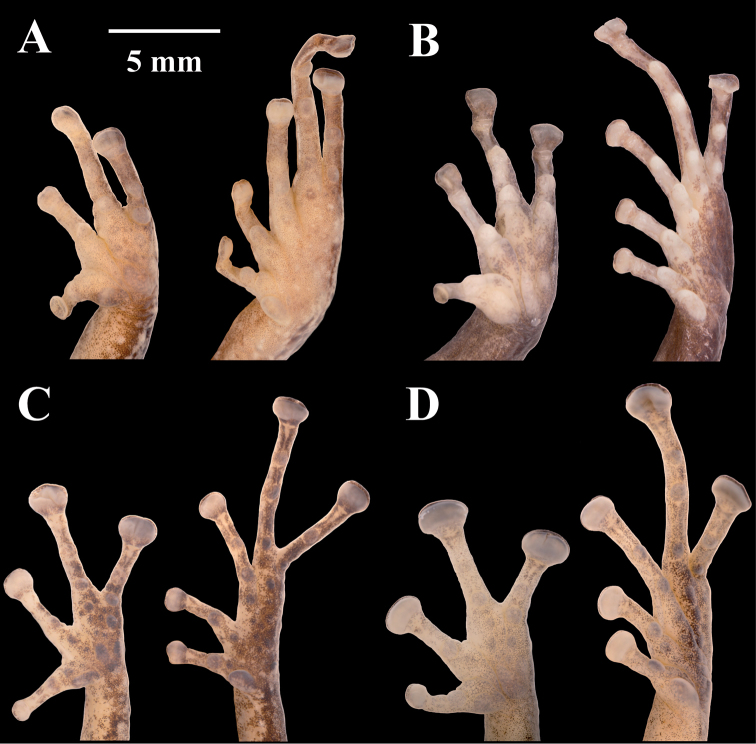
Palmar and plantar surfaces of *Pristimantis lutzae* sp. nov., *Pristimantis multicolor* sp. nov., *Pristimantis nangaritza* sp. nov., and *Pristimantis phoxocephalus*. Photographs of hand and foot of the holotypes (except for *P. phoxocephalus*) of the following species: **A***Pristimantis lutzae* sp. nov.: QCAZ 37546, female **B***Pristimantis multicolor* sp. nov.: QCAZ 47213, male **C***Pristimantis nangaritza* sp. nov.: QCAZ 41710, female **D***Pristimantis phoxocephalus*: QCAZ 58463, female from the type locality. All specimens are shown at the same scale.

**Figure 9.**
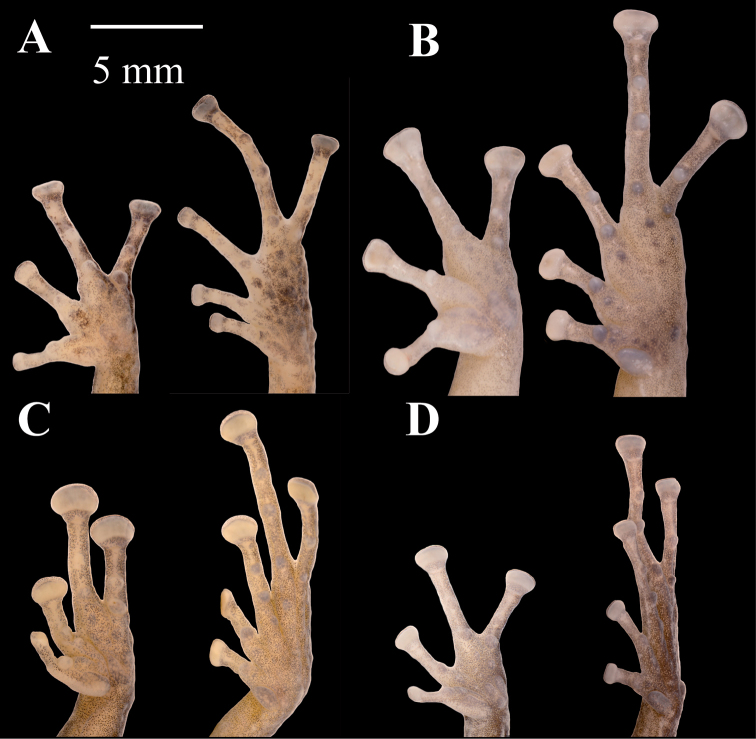
Palmar and plantar surfaces of *Pristimantis teslai* sp. nov., *Pristimantis torresi* sp. nov., *Pristimantis totoroi* sp. nov., and *Pristimantis verrucolatus* sp. nov. Photographs of hand and foot of the holotypes of the following species: **A***Pristimantis teslai* sp. nov.: QCAZ 46213, male **B***Pristimantis torresi* sp. nov.: QCAZ 47342, female **C***Pristimantis totoroi* sp. nov.: QCAZ 25105, male **D***Pristimantis verrucolatus* sp. nov.: QCAZ 46982, male. All specimens are shown at the same scale.

This clade is strongly supported by genetic evidence (Fig. 3). Morphological synapomorphies are unknown. Members of this clade are characterized by: (i) dorsolateral folds absent (except for *P. atratus*); (ii) cranial crests absent (except for *P. atratus*, *P. percultus*, and *P. spinosus*); (iii) tympanic membrane and tympanic annulus prominent (both absent in *P. philipi*); (iv) dentigerous processes of vomer present; (v) small to prominent tubercles on heel; (vi) fingers with lateral fringes; (vii) basal webbing between toes (except for *P. balionotus*, *P. hampatusami*, and *P. philipi* which lack webbing); (viii) Toe V longer or much longer than Toe III (Figs 7–9); (ix) in life, groins and concealed surfaces of thighs with distinctive coloration patterns including flash colors and light or bright colored flecks or spots on a darker background; colors, shapes and sizes of these ornaments are variable among species; (x) SVL females 24.9–46.8 mm; SVL males 16.1–34.5 mm. Species of this clade may bear a fleshy keel or a papilla at the tip of the snout, and lateral, middorsal, or postocular folds.

#### Content.

This clade comprises 23 described species (11 of them described below): *P. atillo* sp. nov., *P. atratus*, *P. balionotus*, *P. chomskyi* sp. nov., *P. cryptomelas*, *P. gagliardoi*, *P. gloria* sp. nov., *P. hampatusami*, *P. jimenezi* sp. nov., *P. lutzae* sp. nov., *P. multicolor* sp. nov., *P. muscosus*, *P. nangaritza* sp. nov., *P. percultus*, *P. philipi*, *P. phoxocephalus*, *P. spinosus*, *P. teslai* sp. nov., *P. tinguichaca*, *P. torresi* sp. nov., *P. totoroi* sp. nov., *P. versicolor*, *P. verrucolatus* sp. nov. This clade encompasses the *P. phoxocephalus* and *P. cryptomelas* species groups.

#### Distribution.

*Huicundomantis* occurs in Eastern and Western Andean slopes and Inter-Andean valleys of southern and central Ecuador, and Eastern Andean slopes of northern Peru. They inhabit the following Natural Regions: Deciduous Costa Forest, Western Foothill Forest, Western Montane Forest, Paramo, Inter-Andean Shrub, Eastern Montane Forest, and Eastern Foothill Forest, between elevations of 230 and 4200 m a.s.l.

#### Etymology.

We name this clade *Huicundomantis* because these frogs are frequently found inside bromeliad plants. Huicundo is a word in Quechua, an indigenous South American language, locally used to referring to bromeliads.

##### *Pristimantis phoxocephalus* species group new taxon

**Definition.** The *P. phoxocephalus* species group is strongly supported in our phylogeny. Members of this group share the following morphological traits: (i) dorsolateral folds absent (except for *P. atratus*); (ii) snout with a fleshy keel or papilla at the tip; (iii) cranial crests absent (except for *P. atratus* and *P. percultus*); (iv) tympanic membrane and tympanic annulus prominent; (v) dentigerous processes of vomer present; (vi) males with vocal slits (except for *P. versicolor*); (vii) fingers and toes with lateral fringes; (viii) basal webbing between toes (except for *P. balionotus* and *P. hampatusami*); (ix) Toe V longer or much longer than Toe III (Fig. 7); (x) in life, groins and concealed surfaces of thighs with distinctive coloration, including flash colors and light or bright colored flecks or spots on a darker background; colors, shapes, and sizes of ornaments variable among species; (xi) SVL females 24.9–46.8 mm; SVL males 16.8–34.5 mm.

**Content.** Currently, the *P. phoxocephalus* group comprises 17 described species (10 of them are described below): *P. atillo* sp. nov., *P. atratus*, *P. balionotus*, *P. chomskyi* sp. nov., *P. gloria* sp. nov., *P. hampatusami*, *P. jimenezi* sp. nov., *P. lutzae* sp. nov., *P. multicolor* sp. nov., *P. percultus*, *P. phoxocephalus*, *P. teslai* sp. nov., *P. tinguichaca*, *P. torresi* sp. nov., *P. totoroi* sp. nov., *P. versicolor*, *P. verrucolatus* sp. nov.

**Distribution.** Eastern and Western Andean slopes and Inter-Andean valleys of central and southern Ecuador, in ten provinces: Azuay, Bolívar, Cañar, Chimborazo, Cotopaxi, El Oro, Loja, Morona Santiago, Tungurahua, and Zamora Chinchipe. They inhabit Deciduous Costa Forest, Western Foothill Forest, Western Montane Forest, Paramo, Inter-Andean Shrub, and Eastern Montane Forest Natural Regions, between 230 and 4100 m a.s.l.

**Remarks.** The *P. phoxocephalus* species group is sister to the *P. cryptomelas* species group.

### Pristimantis atillo sp. nov.

Taxon classificationAnimaliaAnuraStrabomantidae

23E6843DA14459C5B4E6D2DAD284E7DA

http://zoobank.org/078D7203-04D4-4509-9769-8DE14FF5B886

#### Common name.

English: Atillo Rain Frog. Spanish: Cutín de Atillo.

#### Holotype.

**Figure 10.**
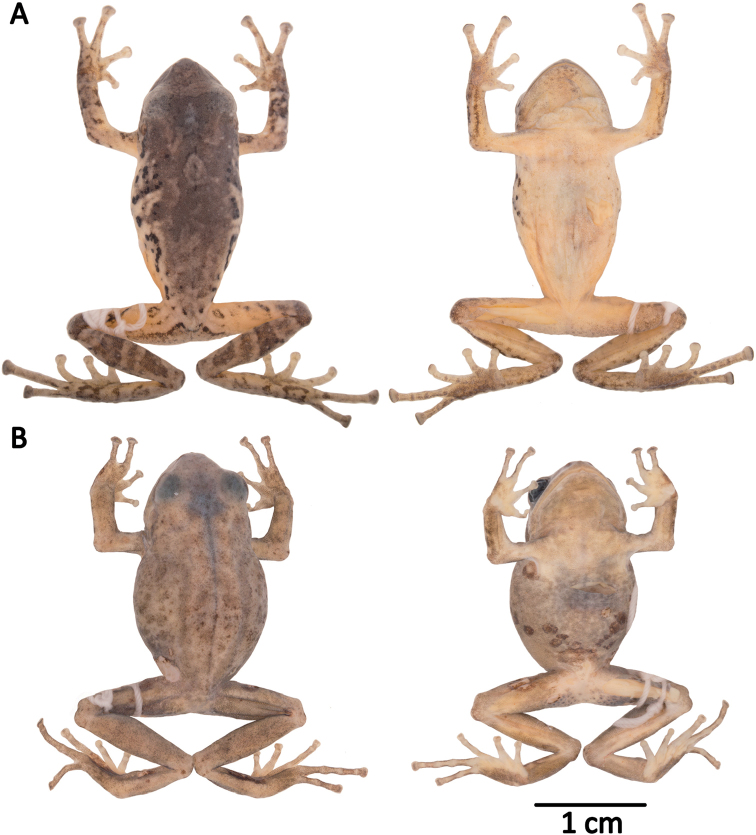
Holotypes of *Pristimantis atillo* sp. nov. and *P. chomskyi* sp. nov. Photographs of preserved holotypes of **A***P. atillo* (QCAZ 42500, male) and **B***P. chompskyi* (QCAZ 47515, male). Dorsal view on the left, ventral view on the right. Specimens are shown at the same scale.

**Figure 11.**
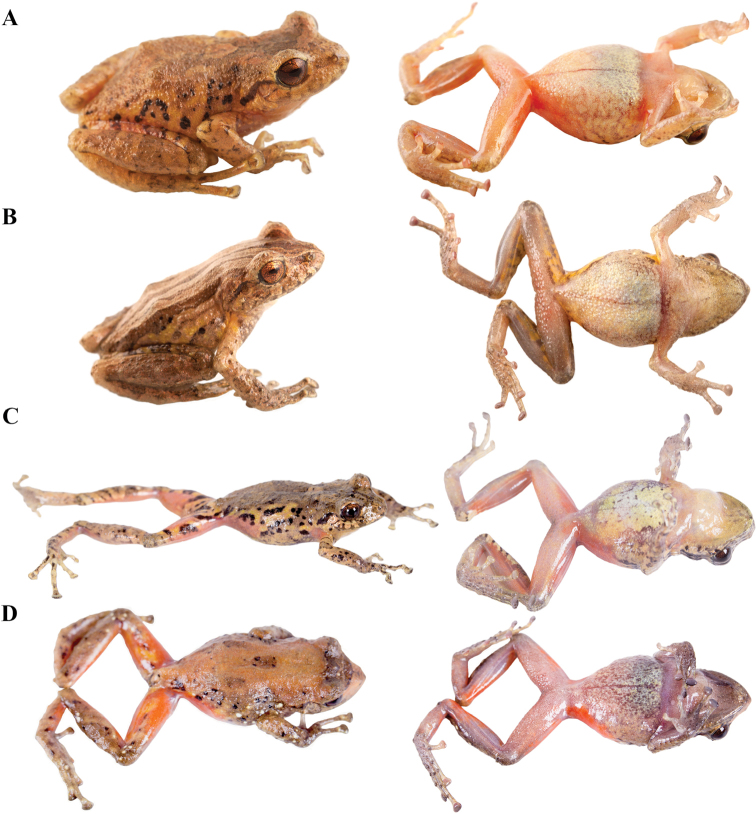
Color variation in live individuals of *Pristimantis atillo* sp. nov. **A**QCAZ 42500 (holotype, male, SVL 26.3 mm) **B**QCAZ 42502 (male, SVL 20.6 mm) **C**QCAZ 59228 (male, SVL 23.7 mm) **D**QCAZ 59261 (male, SVL 25.3 mm). Dorsolateral view on the left, ventral view on the right.

QCAZ 42500, an adult male from Sangay National Park, Ranger Station, Atillo, Morona Santiago Province, Ecuador (2.1867S, 78.4963W, 3400 m), collected by Elicio Tapia on May 19, 2009. Figures 10A, 11A.

#### Paratypes

**(44: 28 males, 5 females, 11 juveniles).** All from Sangay National Park, nearby the type locality. Ecuador: Chimborazo Province: QCAZ 2298–302, QCAZ 2304, adult males, QCAZ 2303, QCAZ 2305–306, juveniles (2.1749S, 78.5047W, 3600 m), collected by Giovanni Onore and Luis Coloma in October 1991; QCAZ 3110, adult female, QCAZ 3111–112, adult males (2.1749S, 78.5047W, 3600 m), collected by Luis Coloma in January 1991; QCAZ 40593, QCAZ 40594, adult females (2.2103S, 78.4991W, 3730 m), collected by Diego Almeida, Galo Díaz, Ítalo Tapia, and Martín Bustamante in May 2003. Morona Santiago Province: QCAZ 31946, adult male (2.1989S, 78.4824W, 3185m), collected by Charles M. Kieswetter and Ítalo Tapia in April 2006; QCAZ 40584, adult male (2.2024S, 78.4697W, 3366 m), collected by Diego Almeida, Galo Díaz, Ítalo Tapia and Martín Bustamante in May 2002; QCAZ 42501, adult female, QCAZ 40897, QCAZ 42483–486, QCAZ 42488–492, QCAZ 42499–500, QCAZ 42502–505, adult males, QCAZ 42493, QCAZ 42496, QCAZ 42498, QCAZ 42506, QCAZ 42512, QCAZ 42543, QCAZ 42548, juveniles (2.1867S, 78.4963W, 3400 m), collected by Elicio Tapia on May 18–19, 2009; QCAZ 59274, adult female, QCAZ 59214, QCAZ 59261, adult males (2.1842S, 78.4972W, 3436 m), collected by Francy Mora, David Velalcázar, Javier Pinto, Luis Tipantiza, Keyko Cruz, Daniel Rivadeneira, Freddy Almeida, Pol Pintanel, and Nadia Páez in March 2015. QCAZ 59228, adult male, QCAZ 59276, juvenile (2.1887S, 78.4823W, 3299 m), collected by Francy Mora, David Velalcázar, Javier Pinto, Luis Tipantiza, Keyko Cruz, Daniel Rivadeneira, Freddy Almeida, Pol Pintanel, and Nadia Páez in March 2015.

#### Diagnosis.

A member of the *Pristimantis phoxocephalus* group having the following combination of characters: (1) skin on dorsum shagreen with or without scattered small subconical tubercles; middorsal fold ill-defined or absent; head with a middorsal longitudinal row of two or more subconical tubercles; dorsolateral folds absent; skin on venter areolate; discoidal fold present or absent; (2) tympanic membrane and tympanic annulus prominent, its upper and posterior margin concealed by supratympanic fold; (3) snout moderately long, acuminate with a fleshy keel in dorsal view, protruding in profile; (4) upper eyelid with one or more small prominent subconical tubercles surrounded by lower tubercles; cranial crests absent; (5) dentigerous processes of vomers low to prominent, oblique, moderately separated, posteromedial to choanae; (6) males having vocal slits, external vocal sac and white nuptial pads; (7) Finger I shorter than Finger II; discs of digits expanded to broadly expanded, rounded to elliptical; (8) fingers with broad lateral fringes; (9) ulnar tubercles low and rounded, sometimes connected by an ill-defined fold; (10) heel bearing one subconical tubercle surrounded or not by smaller rounded tubercles; outer edge of tarsus bearing low subconical tubercles; inner edge with or without ill-defined tubercles, short inner tarsal fold present; (11) inner metatarsal tubercle ovoid, elevated, five times the size of round outer metatarsal tubercle; supernumerary plantar tubercles numerous; (12) toes with lateral fringes, less conspicuous than those on fingers; basal webbing on feet (Fig. 7A); Toe V longer or much longer than Toe III (disc on Toe III reaches or exceeds distal edge of the penultimate subarticular tubercle on Toe IV, disc on Toe V reaches or exceeds distal edge of the distal subarticular tubercle on Toe IV); toe discs nearly as large as those on fingers; (13) in life, dorsal coloration varies from pale to dark brown; black or brown interorbital stripe, supratympanic stripe, and labial bars present; flanks with black dots and flecks arranged in a diagonal pattern, bordering light reticulations; groins, axils, concealed surfaces of thighs, and shanks orange; venter and throat cream to white; iris copper with a faint medial horizontal darker streak and black reticulations (Fig. 11); (14) average SVL in adult females: 31.3 ± 2.3 mm (29.4–35.3 mm; *n* = 5); in adult males: 24.7 ± 2.5 mm (17.5–28.1 mm; *n* = 29).

#### Comparison with other species.

*Pristimantis atillo* is similar to other species with acuminate and protruding snouts of this group such as *P. jimenezi* sp. nov., *P. phoxocephalus*, *P. teslai* sp. nov., *P. torresi* sp. nov., *P. totoroi* sp. nov., and *P. verrucolatus* sp. nov. The bright orange coloration of its groins and posterior surfaces of thighs distinguishes it from them (brown with small light brown to yellow spots in *P. jimenezi* sp. nov.; yellow with black reticulations in *P. phoxocephalus*; dark brown with yellow irregular blotches in *P. teslai* sp. nov.; brown with or without yellow spots in *P. torresi* sp. nov.; reddish brown with small light brown to yellow spots in *P. verrucolatus* sp. nov.). *Pristimantis atillo* can be further distinguished from *P. jimenezi* sp. nov. and *P. phoxocephalus* by the presence of its characteristic black dots in the flanks (black dots absent in *P. jimenezi* sp. nov. and *P. phoxocephalus*). *Pristimantis atillo* differs from *P. teslai* sp. nov. by the dorsal skin texture (shagreen in *P. atillo*; tuberculate in *P. teslai* sp. nov.). The copper coloration of the iris differentiates *P. atillo* from *P. torresi* sp. nov. and *P. totoroi* sp. nov., whose iris is golden with a red streak. Furthermore, tubercles and folds of *P. totoroi* sp. nov. are more prominent than those of *P. atillo*, and its head, relative to the body, is longer (males Wilcoxon’s *Z* = 4.50124, *p* < 0.001, HL/SVL = 33.5–38.5% in *P. atillo*, 34.2–38.7% in *P. totoroi* sp. nov.; females *Z* = 2.43599, *p* = 0.0149, HL/SVL = 33.1–36.6% in *P. atillo*, 35.2–37.6% in *P. totoroi* sp. nov.). *Pristimantis verrucolatus* sp. nov. differs from *P. atillo* in having large tubercles and warts on its flanks. *Pristimantis atillo* is similar to *P. modipeplus* (Lynch 1981) in coloration pattern; *P. modipeplus* has a subacuminate snout in dorsal view and rounded in profile, lacks basal webbing between toes, and large females bear low cranial crests (Lynch 1981); meanwhile, *P. atillo* has an acuminate and protruding snout with a keel at the tip, presents basal webbing between toes and has no cranial crests.

#### Description of the holotype.

Adult male (QCAZ 42500, SC28232). Measurements (in mm): SVL 26.3; TL 12.0; FL 11.9; HL 8.9; HW 9.1; ED 2.6; TD 1.3; IOD 2.8; EW 2.4; IND 2.1; EN 2.7; TED 1.1. Head narrower than body, slightly wider than long; snout moderately long, acuminate with triangular fleshy keel in dorsal view, protruding in profile; nostrils slightly protuberant, directed laterally with slight dorsal inclination; canthus rostralis concave in dorsal view, rounded in cross section; loreal region slightly concave; cranial crests absent; upper eyelid bearing one small but distinct conical tubercle surrounded by few indistinct smaller tubercles; tympanic membrane and annulus prominent, its upper and posterior margins concealed by supratympanic fold; two enlarged conical postrictal tubercle surrounded by indistinct low tubercles. Choanae median, elliptical, non-concealed by palatal shelf of maxilla; dentigerous processes of vomers prominent, oblique, moderately separated, posteromedial to choanae; each vomer bearing several indistinct teeth; tongue slightly longer than wide, posteriorly notched, posterior two-thirds free; vocal slits slightly curved, located at posterior half of mouth floor in between tongue and margin of jaw; vocal sac present.

Skin on dorsum shagreen; head with a row of two middorsal tubercles; dorsolateral folds absent; flanks with slightly larger tubercles than those on dorsum; skin on throat, chest, belly and ventral surfaces of thighs areolate; discoidal fold absent. Two distinct, low and round ulnar tubercles connected by an ill-defined fold; white nuptial pads present; palmar tubercles prominent, outer palmar tubercle bifid, slightly bigger than ovoid thenar tubercle; subarticular tubercles prominent, rounded; distinct, low supernumerary tubercles at base of fingers; fingers with broad lateral fringes; Finger I shorter than Finger II; discs on fingers expanded and elliptical; ventral pads on fingers surrounded by circumferential grooves (Fig. 7A).

Hindlimbs slender; dorsal surfaces of hindlimbs with scattered low round tubercles; posterior surfaces of thighs smooth, ventral surfaces of thighs areolate; heel bearing one low conical tubercle surrounded by some lower rounded tubercles; outer edge of tarsus bearing low subconical tubercles; inner edge of tarsus with ill-defined tubercles; short inner tarsal fold present; inner metatarsal tubercle ovoid, elevated, five times the size of circular, rounded, outer metatarsal tubercle; plantar surface with numerous indistinct supernumerary tubercles; subarticular tubercles prominent, rounded; toes with broad lateral fringes; small basal webbing between toes; discs nearly as large as those on fingers; discs on toes expanded, elliptical; all toes having ventral pads and circumferential grooves; relative lengths of toes: I<II<III<V<IV; Toe V much longer than Toe III (disc on Toe III reach distal edge of penultimate subarticular tubercle on Toe IV, disc on Toe V reaches distal edge of distal subarticular tubercle on Toe IV; Fig. 7A). Coloration of the holotype in preservative and life is shown in Figures 10, 11.

#### Variation.

**Figure 12.**
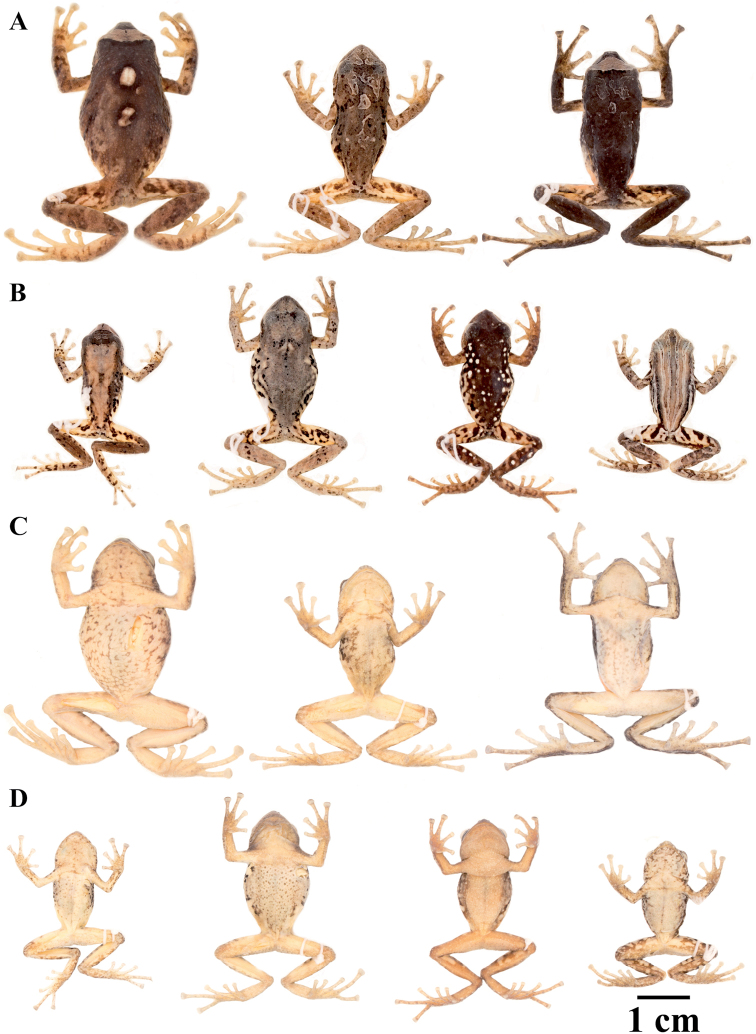
Color variation in preserved individuals of *Pristimantis atillo* sp. nov. **A** Dorsal view of (from left to right): QCAZ 40593 (female), QCAZ 42501 (female), QCAZ 59274 (female) **B** dorsal view of: QCAZ 42498 (juvenile female), QCAZ 42503 (male), QCAZ 2302 (male), QCAZ 42548 (juvenile female) **C** ventral view of specimens in (**A**) **D** ventral view of specimens in **B**. See Suppl. material 2 for locality data. All specimens are shown at the same scale.

This section is based on 45 preserved specimens of the type series and photographs available for 11 individuals. Variation in living and preserved individuals is shown in Figures 11, 12. Coloration in life is mentioned in parenthesis. Dorsum varies from pale to dark brown with or without lighter irregular marks or white spots scattered on dorsum, few individuals have several thin parallel longitudinal stripes or a middorsal band (i.e., longitudinal pattern); the interorbital band can be thin to broad, paler or darker than background and is absent in individuals with longitudinal pattern; interscapular blotch is often present. Flanks have pale oblique reticulations usually surrounded by dark stripes formed by rows of black flecks. Groins, anterior and posterior surfaces of thighs cream (orange, in most cases surrounded by yellow blotches; an individual has yellow groins (QCAZ 42502)). Dorsal surfaces of thighs with brown to black oblique stripes. Venter and throat coloration varies from cream to white (cream to white with different levels of transparency, with or without ill-defined yellow blotches) with or without black flecks. Iris is reddish to orangey copper with a faint medial horizontal darker streak and thin black reticulations; sclera varies from white to light blue.

*Coloration of holotype in preservative*. Dorsum brown with lighter markings including W-shaped scapular mark, interscapular blotch, and faint irregular reticulations; head with light brown interorbital band; face with faint canthal and labial bars; dark brown supratympanic bar; flanks light brown with pale oblique reticulations surrounded by rows of black flecks; armpits, groins, anterior and posterior surfaces of thighs cream; dorsal surfaces of hindlimbs brown with lighter transversal bands; limbs with scattered black flecks; ventral surfaces cream; plantar and palmar surfaces dirty cream (Fig. 10A).

*Coloration of holotype in life*. Based on studio photographs (Fig. 11A). Dorsum brown with cream light markings including a W-shaped scapular mark, interscapular blotch, and irregular reticulations; head with light brown interorbital band; face with faint brown canthal and labial bars; dark brown supratympanic bar; flanks light brown with yellow reticulations surrounded by rows of black flecks; armpits, groins, anterior and posterior surfaces of thighs and shanks orange; dorsal surface of hindlimbs with faint yellowish brown transverse bands and scattered black flecks; venter yellowish white; vocal sac, chest, and ventral surfaces of forelimbs surfaces transparent pinkish cream; ventral surface of thighs yellow suffused with orange; plantar and palmar surfaces dirty cream; iris copper with a faint medial horizontal darker streak and thin black reticulations; light blue sclera.

#### Distribution, natural history, and conservation status.

This species is only known from the type locality, surroundings of Lagunas de Atillo, and nearby locations at Sangay National Park, between 3185 and 3730 m a.s.l (Fig. 1). This corresponds to the Eastern Montane Forest and Paramo biogeographic regions. Most individuals collected at night were found active on low vegetation, from ground level up to 1 m above the ground; an individual was collected from a bromeliad 2.5 m above the ground. During the day, they were found beneath rocks. Most collections were made on roadsides. Calling males have been found in March.

According to available data, this species has a very restricted distribution (Extent of Occurrence 7 km^2^, Area of Occupancy 16 km^2^). However, less accessible adjacent areas in Sangay National Park are unexplored and represent potential distribution areas for this species. Therefore, we consider this species to be Data Deficient (IUCN 2017).

#### Etymology.

The specific epithet refers to the type locality of this species, the surroundings of Lagunas de Atillo, a lake complex in Sangay National Park, a UNESCO World Heritage Site.

### Pristimantis chomskyi sp. nov.

Taxon classificationAnimaliaAnuraStrabomantidae

E412B6355C165C5F8F76C11949A51BEA

http://zoobank.org/710B5B6E-FECA-46AC-A7F8-A62DF3B7747B

#### Common name.

English: Chomsky’s Rain Frog. Spanish: Cutín de Chomsky.

#### Holotype.

**Figure 13.**
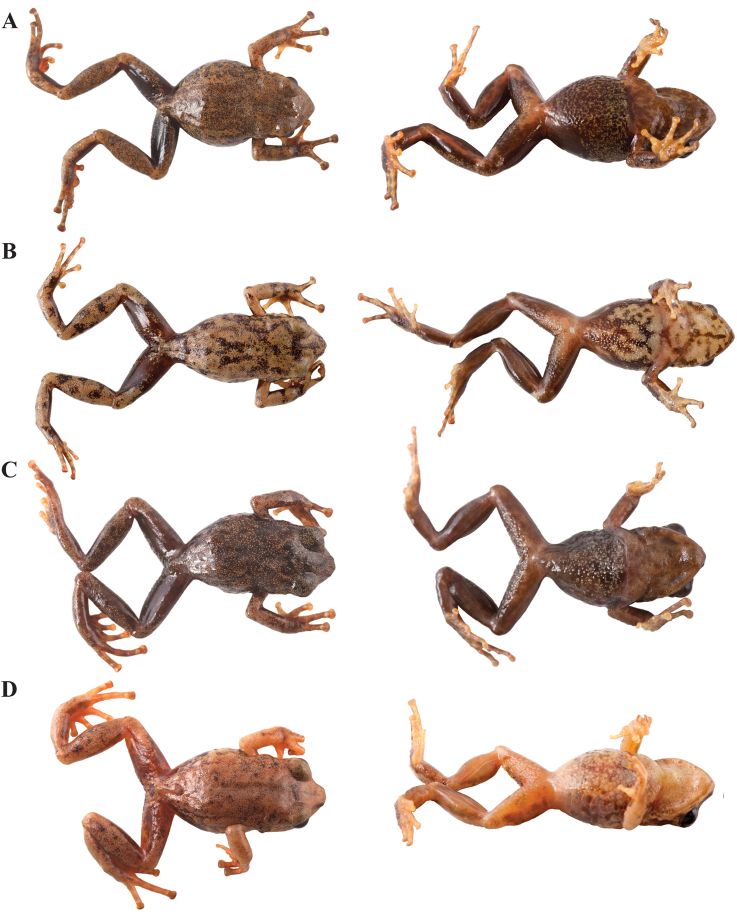
Color variation in live individuals of *Pristimantis chomskyi* sp. nov. **A**QCAZ 47515 (holotype, male, SVL 24.0 mm) **B**QCAZ 45669 (juvenile male, SVL 21.4 mm) **C**QCAZ 45665 (juvenile female, SVL 19.7 mm) **D**QCAZ 45666 (juvenile female, SVL 21.9 mm). Dorsal view on the left, ventral view on the right.

QCAZ 47515, an adult male from Tapichalaca Reserve, Zamora Chinchipe Province, Ecuador (4.4730S, 79.1930W, 3366 m), collected by Elicio E. Tapia on September 27, 2009. Figures 10B, 13A.

#### Paratypes

**(11: 2 adult males, 9 juveniles).**QCAZ 45670, QCAZ 47733, adult males, QCAZ 45664–669, QCAZ 45677, QCAZ 47730–731, juveniles, collected with the holotype.

#### Diagnosis.

**Figure 14.**
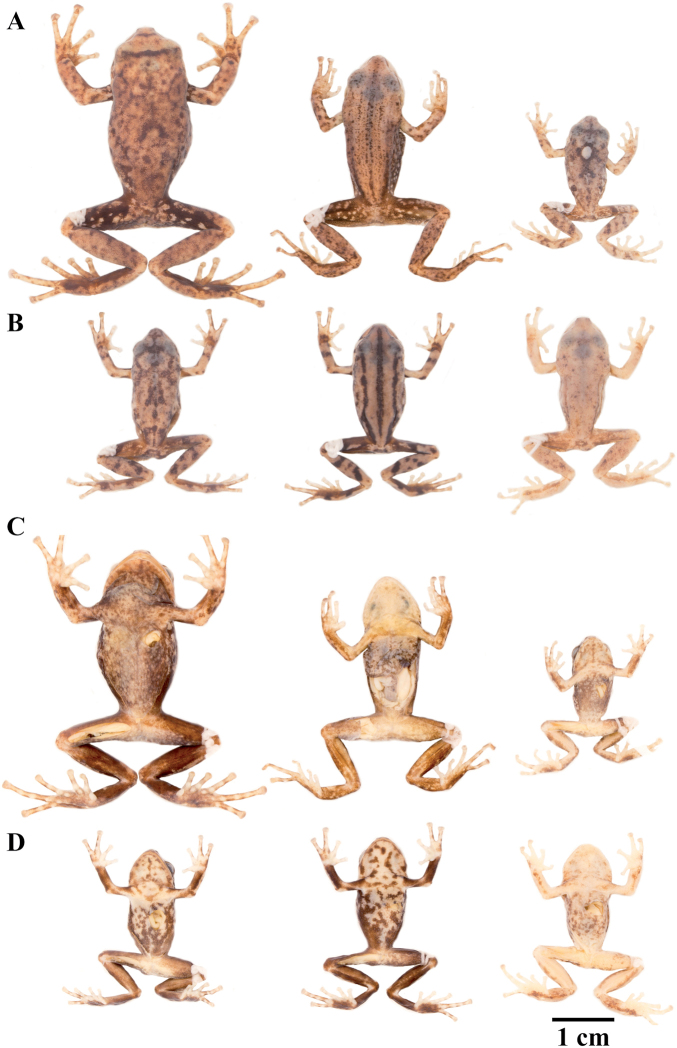
Color variation in preserved individuals of *Pristimantis chomskyi* sp. nov. **A** Dorsal view of (from left to right): QCAZ 45670 (male), QCAZ 47731 (juvenile female), QCAZ 45667 (juvenile male), **B** Dorsal view of: QCAZ 45669 (juvenile male), QCAZ 45670 (male), QCAZ 45666 (juvenile female) **C** Ventral view of specimens in (**A**) **D** Ventral view of specimens in **B** See Suppl. material 2 for locality data. All specimens are shown at the same scale.

A species of *Pristimantis* having the following combination of characters: (1) skin on dorsum shagreen, skin on flanks with large warts, skin on venter areolate to coarsely areolate; discoidal fold absent; dorsolateral folds absent; (2) tympanic membrane and tympanic annulus prominent, its upper and posterolateral margin concealed by thick supratympanic fold; (3) snout short, subacuminate in dorsal view, rounded in lateral view, with or without a small papilla at the tip; (4) upper eyelid lacking tubercles; cranial crests absent; (5) dentigerous processes of vomers low to prominent, oblique, moderately separated, posteromedial to choanae; (6) vocals slits, vocal sac and nuptial pads present in adult males; (7) Finger I shorter than Finger II; discs of digits expanded, rounded to elliptical; (8) fingers with lateral fringes; (9) ulnar tubercles low, diffuse; (10) heel bearing one small low rounded tubercle; inner edge of tarsus with or without a low tubercle or small fold; outer edge of tarsus with or without indistinct tubercles; (11) inner metatarsal tubercle elliptical, elevated, about 4 times the size of round outer metatarsal tubercle; supernumerary tubercles low; (12) toes with lateral fringes; basal webbing on feet; Toe V longer to much longer than Toe III (disc on Toe III reaches or exceeds distal edge of the penultimate subarticular tubercle on Toe IV, disc on Toe V reaches the middle to distal edge of the distal subarticular tubercle on Toe IV); toe discs smaller than those on fingers (Fig. 7B); (13) in life, dorsum dark brown to orange; head bears brown supratympanic and canthal stripes; groins, axils and concealed surfaces of hindlimbs dark chocolate brown suffused or not with orange, with or without small cream flecks; venter cream to dusty brown with or without dark markings; iris orange with a faint reddish brown medial horizontal streak and thin black reticulations (Fig. 13); (14) average SVL in adult males: 28.0 ± 4.2 mm (24.0–32.4 mm; *n* = 3); females: unknown.

#### Comparison with other species.

*Pristimantis chomskyi* is similar to *P. balionotus*, *P. gloria* sp. nov., *P. lutzae* sp. nov., and *P. multicolor* sp. nov. The most similar is its sister species *P. multicolor* sp. nov. The region above supratympanic fold is more swollen in *P. chomskyi*. In addition, *P. chomskyi* has a smaller tympanum (males *Z* = 2.38157, *p* = 0.0172, TD/SVL = 4.5–4.7% in *P. chomskyi*, 4.9–6% in *P. multicolor* sp. nov.). *Pristimantis balionotus* can be distinguished from *P. chomskyi* because it has a tuberculate dorsal skin, lacks basal webbing between toes, and its iris is bronze with a red medial streak (shagreen dorsal skin, basal webbing between toes, iris orange with a faint reddish-brown streak in *P. chomskyi*). *Pristimantis chomskyi* is easy to distinguish from *P. gloria* sp. nov. by the coloration of the iris (orange with a faint reddish brown streak and thin black reticulations in *P. chomskyi*; light silver to cream with wide black reticulations and a red streak in *P. gloria* sp. nov.) and groins (dark chocolate brown with or without small cream flecks in *P. chomskyi*; pinkish to purplish brown with irregular cream to light brown flecks or spots in *P. gloria* sp. nov.), and the texture of the dorsal skin (shagreen without a middorsal fold in *P. chomskyi*; tuberculate to warty with a middorsal fold in *P. gloria* sp. nov.). Furthermore, *P. gloria* sp. nov. is smaller (males *Z* = -2.31490, *p* = 0.0206, SVL = 24.0–32.4 mm in *P. chomskyi*, 16.7–24.7 mm in *P. gloria* sp. nov.), has larger tympanum (males *Z* = 2.66173, *p* = 0.0078, TD/SVL = 4.5–4.7% in *P. chomskyi*, 5.1–6.2% in *P. gloria* sp. nov.) and smaller eye (males *Z* = -2.50743, *p* = 0.0122, ED/SVL = 11.1–12% in *P. chomskyi*, 11–13.8% in *P. gloria* sp. nov.), relative to its body length. *Pristimantis lutzae* sp. nov. differs from *P. chomskyi* in having a golden to creamy brown iris with a reddish brown streak, a larger tympanum (males *Z* = 2.45677, *p* = 0.0140, TD/SVL = 4.5–4.7% in *P. chomskyi*, 5–5.4% in *P. lutzae* sp. nov.) and smaller eye (males *Z* = -2.45677, *p* = 0.0140, ED/SVL = 11.1–12% in *P. chomskyi*, 10.3–12.1% in *P. lutzae* sp. nov.), relative to its body length.

#### Description of the holotype.

An adult male (QCAZ 47515, SC29227). Measurements (in mm): SVL 24.0; TL 12.0; FL 11.6; HL 8.8; HW 9.4; ED 2.9; TD 1.1; IOD 3.3; EW 2.6; IND 2.4; EN 2.4; TED 1.1. Head wider than long, narrower than body; snout subacuminate in dorsal view, rounded in profile, with a small papilla at the tip; cranial crests absent; nostrils slightly protuberant, directed anterolaterally; canthus rostralis slightly concave in dorsal view, rounded in cross section; loreal region concave; upper eyelid lacking tubercles; tympanic annulus prominent, its upper and posterolateral edge concealed by thick, heavy supratympanic fold; tympanic membrane distinct; two prominent postrictal tubercle not surrounded by smaller tubercles. Choanae median, ovoid, non-concealed by palatal shelf of maxilla; dentigerous processes of vomers prominent, oblique, moderately separated, positioned posteromedial to choanae; each vomer bearing several indistinct teeth; tongue slightly longer than wide, not notched, posterior half free; vocal slits slightly curved, positioned at posterior half of mouth floor in between tongue and margin of jaw; small vocal sac.

Dorsal surfaces of body shagreen; dorsolateral folds absent; skin on flanks bearing low rounded warts; skin on chest and belly areolate, that on throat shagreen, ventral surfaces of limbs smooth, ventral surfaces of thighs coarsely areolate; discoidal fold absent. Diffuse ulnar tubercles; nuptial pads present; outer palmar tubercle bifid, as large as ovoid thenar tubercle; subarticular tubercles prominent, rounded; large supernumerary tubercles at base of fingers, distinct; fingers bearing lateral fringes; Finger I shorter than Finger II; discs on fingers expanded and rounded; pads on fingers surrounded by circumferential grooves on all fingers (Fig. 7B).

Hindlimbs slender; dorsal surfaces of hindlimbs shagreen; posterior surfaces of thighs smooth, ventral surfaces of thighs coarsely areolate; heel bearing one low and rounded tubercle; outer and inner edge of tarsus lacking tubercles; inner metatarsal tubercle elliptical, elevated, 4 times the size of oval outer metatarsal tubercle; plantar surface with small, low and rounded supernumerary tubercles; subarticular tubercles prominent, rounded; toes bearing lateral fringes; basal webbing between toes III and IV, and IV and V; discs on toes smaller than those on fingers, expanded and rounded; all toes having pads surrounded by circumferential grooves; relative lengths of toes: I < II < III < V < IV; Toe V longer than Toe III (disc on Toe III reaches the distal edge of penultimate subarticular tubercle on Toe IV, disc on Toe V reaches the distal edge of distal subarticular tubercle on Toe IV; Fig. 7B). Coloration of the holotype in life and preservative is shown in Figures 10A, 13A.

*Coloration of holotype in preservative*. Dorsal surfaces of body and flanks cream covered by minute brown spots evenly distributed; brown supratympanic and canthal stripes; groins, anterior, and posterior surfaces of thighs, shanks and tarsus brown; venter, belly and throat cream suffused with dusty brown irregular spots; belly with dark brown blotches (this pattern does not correspond with coloration in life, probably effect of preservation); ventral surfaces of limbs dusty cream; ventral surfaces of fingers and toes cream (Fig. 10B).

*Coloration of holotype in life*. Based on studio photographs. Dorsal surfaces of body and flanks orange-hued brown covered by minute chocolate brown spots evenly distributed; brown supratympanic and canthal stripes; groins, anterior and posterior surfaces of thighs, shanks and tarsus chocolate brown; venter yellowish cream covered by chocolate brown spots coinciding with areolation pattern; throat orange suffused with brown; ventral surfaces of thighs, shanks, and tarsus reddish orange suffused with brown; plantar and palmar surfaces orange; iris orange with a faint reddish brown medial horizontal streak and thin black reticulations (Fig. 13A).

#### Variation.

Based on the 12 preserved specimens of the type series and photographs from eight individuals. Variation in life and preservative is shown in Figures 13, 14. Coloration in life is given in parenthesis. Dorsum and flank coloration vary from brown to brownish cream (dark brown to orange). Dorsal markings may be absent or present as longitudinal stripes or dark blotches evenly scattered; one individual has a pale blotch on dorsum. All individuals have brown supratympanic and canthal stripes; some also have dark interorbital stripe and labial bars. Color of groins, armpits, dorsal surfaces of thighs, concealed surfaces of thighs, shanks and tarsus is dark brown (dark chocolate brown, suffused or not with orange, with or without small cream flecks), few individuals with small pale flecks; dorsal surfaces of thighs and groins can have irregular pale spots. Limbs bear scattered small dark spots arranged or not as transversal bands. Venter varies from cream to dusty brown with or without dark markings (orange, cream, or dusty brown, with or without darker flecks or mottling). The iris is orange with a faint red medial streak and thin black reticulations. Sclera varies from white to light blue.

#### Distribution, natural history, and conservation status.

This species is only known from the type locality, Tapichalaca Reserve, Zamora Chinchipe Province, Ecuador at 3366 m a.s.l (Fig. 1), which corresponds to the Paramo biogeographic region. All individuals were found at night inside bromeliads, mostly of the genus *Puya*. Tapichalaca Reserve is adjacent to Podocarpus National Park (northward) and Yacuri National Park (southward). Both areas are unexplored and represent potential habitats for this species. Therefore, we assign *P. chomskyi* to the Data Deficient Red List Category (IUCN 2017).

#### Etymology.

The specific epithet is a noun in the genitive case and is a patronym for Noam Chomsky, US born theoretical linguist and one of the most cited modern scholars. Chomsky is the founder of modern linguistics. He developed the concept of “universal grammar,” an innate cognitive capacity, shared by all humans, which allows to learn and communicate through complex speech. Chomsky was professor at the Massachusetts Institute of Technology between 1950 and 2017. He is currently professor at Arizona State University.

### Pristimantis gloria sp. nov.

Taxon classificationAnimaliaAnuraStrabomantidae

0F786388E489523FB2AA9523676E4325

http://zoobank.org/115BC459-FCEA-4950-8B80-7E5B7D6DB8C4

#### Common name.

English: Gloria’s Rain Frog. Spanish: Cutín de Gloria.

#### Holotype.

**Figure 15.**
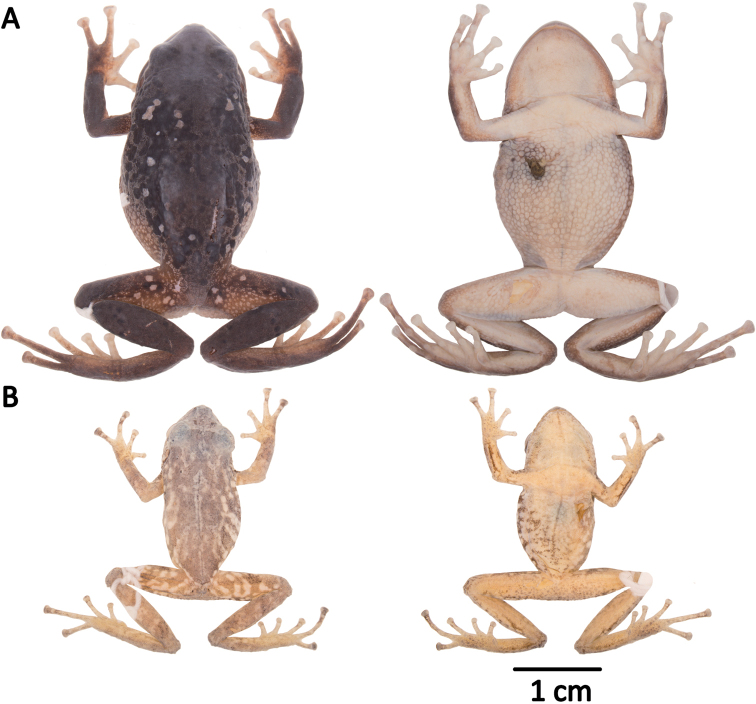
Holotypes of *Pristimantis gloria* sp. nov. and *P. jimenezi* sp. nov. Photographs of preserved holotypes of **A***P. gloria* (QCAZ 57201, female) and **B***P. jimenezi* (QCAZ 45170, male). Dorsal view on the left, ventral view on the right. Specimens are shown at the same scale.

**Figure 16.**
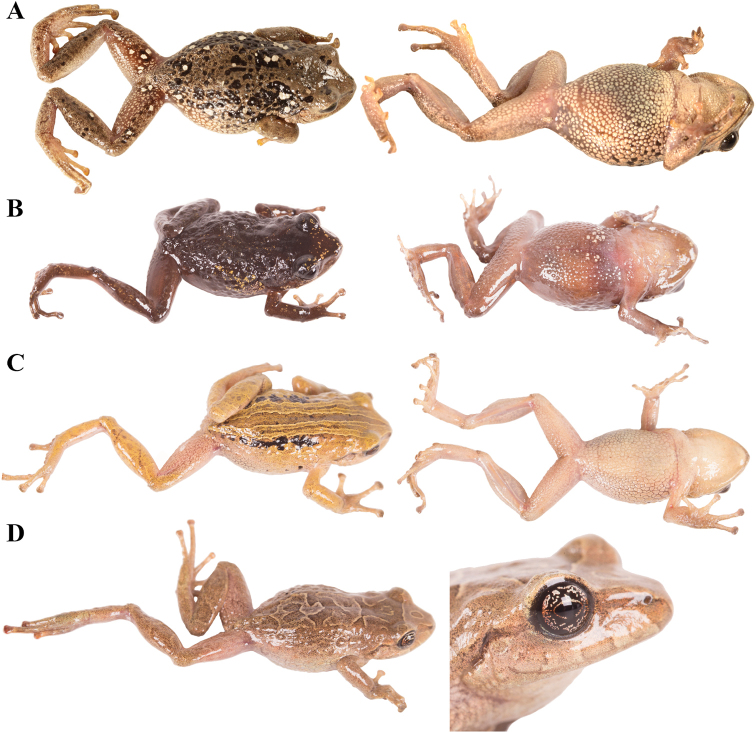
Color variation in live individuals of *Pristimantis gloria* sp. nov. **A**QCAZ 57201 (holotype, female, SVL 35.8 mm) **B**QCAZ 62778 (male, SVL 19.7 mm) **C**QCAZ 62780 (juvenile female, SVL 23.5 mm) **D**QCAZ 62789 (juvenile female, SVL 21.8 mm) For **A–C** dorsolateral view on the left, ventral view on the right. For **D** dorsolateral view on the left, close up of the eye in the right.

QCAZ 57201, a gravid female from Cochapata, Azuay Province, Ecuador (3.4284S, 79.0699W, 3100 m), collected by Edwin Carrillo on May 1, 2014. Figures 15A, 16A.

#### Paratypes

**(59: 14 females, 24 males, 21 juveniles).** Ecuador: Azuay Province: QCAZ 3051–052, QCAZ 3054, adult males, QCAZ 3053, juvenile, from 7 km E Sigsig (3.0973S, 78.7970W, 2890 m); QCAZ 3055, juvenile, from 8 km E Sigsig (3.1025S, 78.7929W, 2920 m); QCAZ 3056, juvenile, from 3 km Sigsig, Sigsig-Las Minas road (3.0750S, 78.8000W, 2460 m), collected by Luis Coloma, Oscar Delgado and Luis Eduardo López on July 25, 1989; QCAZ 3452, male, QCAZ 3451, juvenile, from 23 km SE Sigsig (3.1794S, 78.7993W, 3318 m), collected by Stella de la Torre, Luis Coloma, and Felipe Campos on November 27, 1992; QCAZ 16449, adult female, QCAZ 16452, adult male, QCAZ 16447–448, juveniles, collected by Luis Eduardo López on September 2001; QCAZ 45289, adult male, collected by Luis Eduardo López on August 28, 2009; QCAZ 56533, QCAZ 56536, adult females, QCAZ 56534, juvenile, collected by Luis Eduardo López on December 6, 2013; QCAZ 62779, QCAZ 62783, QCAZ 62788, QCAZ 62791, adult females, QCAZ 62778, QCAZ 62782, QCAZ 62787, adult male, QCAZ 62780, QCAZ 62789, juveniles, collected by Luis Eduardo López on December 13, 2015, from Patococha (3.0040S, 78.6658W, 3416 m); QCAZ 29133, juvenile, from Matanga (3.1779S, 78.8000W, 3325 m), collected by Luis Coloma on December 1, 1991; QCAZ 30737, female, from 11.1 km W Azuay-Morona Santiago border (2.9480S, 78.7120W, 3110 m), collected by Luis Coloma and John Wiens on April 20, 1990; QCAZ 30738, adult male, QCAZ 30739, juvenile, from 8.1 km W Azuay-Morona Santiago border (2.9640S, 78.7020W, 3140 m), collected by John Wiens on April 20, 1990; QCAZ 30740–745, juveniles from 10 km S Cutchil (3.1340S, 78.8130W, 2900 m), collected by Luis Coloma and John Wiens on April 29, 1990; QCAZ 56463, adult male from Chiguinda, Páramo de Matanga (3.1885S, 78.7922W, 3327 m), collected by Jorge Brito on October, 2013. Loja Province: QCAZ 31455, QCAZ 31457, adult females, from Fierro Urcu (3.7104S, 79.3049W, 3439 m), collected by Ítalo G. Tapia on March 2006. Morona Santiago Province: QCAZ 17143, juvenile female, from Gualaceo-Macas road (3.0097S, 78.6650W, 3462 m), collected by Omar Torres, Jennifer Pramuk and Lena Echelle on September 14, 2001; QCAZ 23933, adult female, QCAZ 23929–932, adult males, from 52 km N El Ideal (3.1831S, 78.7961W, 3525 m), collected by Santiago Ron and Giovanna Romero on April 10, 2003; QCAZ 26367, QCAZ 26370, adult females, QCAZ 26368–369, QCAZ 26372–373, adult males, QCAZ 26371, QCAZ 26375, juveniles, from Gualaceo-Plan de Milagro road (3.0007S, 78.6616W, 3372 m), collected by Andrés Merino, Ítalo Tapia and Erik Wild on August 10, 2003; QCAZ 27280, adult female, QCAZ 27276–279, QCAZ 27281, adult males, from Gualaceo-Plan de Milagro road (3.0029S, 78.6572W, 3096 m), collected by David A. Kizirian and Juan Carlos on November 2, 2000; QCAZ 40784, juvenile, from Sigsig-Gualaquiza road (3.1850S, 78.7962W, 3322 m), collected by Omar Torres, Ernesto Arbeláez, Amaranta Carvajal and David Salazar on June 17, 2008.

#### Referred specimens.

KU 218035 from 8.1 km W Morona Santiago border, Gualaceo-Limón road (2.9645S, 78.7016W, 3145 m) Azuay Province, Ecuador.

#### Diagnosis.

A species of *Pristimantis* having the following combination of characters: (1) skin on dorsum tuberculate to warty; tubercles and warts are more prominent in posterior dorsum; middorsal fold present; dorsolateral folds absent; skin on flanks bears large warts and more prominent tubercles than those on dorsum; skin on venter coarsely areolate; (2) tympanic membrane and tympanic annulus prominent, its upper and posterolateral margin concealed by supratympanic fold; (3) snout moderately long, subacuminate in dorsal view, rounded in profile; with or without a small papilla at the tip; (4) upper eyelid without conspicuous tubercles; cranial crests absent; (5) dentigerous processes of vomers low to prominent, oblique, moderately separated, posteromedial to choanae; (6) vocal slits, vocal sac and nuptial pads present in adult males; (7) Finger I shorter than Finger II; discs of digits expanded, truncate to elliptical; (8) fingers with broad lateral fringes; (9) indistinct ulnar tubercles; (10) heel bearing a low rounded tubercle, surrounded by several smaller; outer edge of tarsus bearing inconspicuous tubercles; inner edge of tarsus bearing a medium to long fold followed by small tubercles; (11) inner metatarsal tubercle ovoid, elevated, six times the size of round outer metatarsal tubercle; supernumerary tubercles low, numerous; (12) toes with broad lateral fringes; basal webbing present; Toe V longer to much longer than Toe III (disc on Toe III reaches or exceeds distal edge of penultimate subarticular tubercle on Toe IV, disc on Toe V reaches middle to distal edge of distal subarticular tubercle on Toe IV); discs on toes smaller than those on fingers, truncate to elliptical (Fig. 7C); (13) in life, dorsum light to dark brown with or without shades of cream, yellow or orange; groins and concealed surfaces of thighs brown, creamish brown, pinkish brown, or purplish brown with irregular cream to brown flecks or spots; background color looks like thin reticulations when flecking is dense; ventral surfaces of body cream to brownish cream; iris cream to light silver bearing wide black reticulations and a red medial streak (Fig. 16); (14) average SVL in adult females: 30.1 ± 3.0 mm (26.7–35.8 mm; *n* = 15); in adult males: 21.7 ± 2.3 mm (16.8–24.7 mm; *n* = 24).

#### Comparison with other species.

Similar species are *P. balionotus*, *P. chomskyi*, *P. lutzae* sp. nov., *P. multicolor* sp. nov., *P. percultus*, and *Pristimantis* sp. (CCS2). *Pristimantis gloria* is readily distinguished from them (except *P. percultus*) by the presence of wide black reticulations in its iris (thin black reticulations in the other species). *Pristimantis gloria* can be further distinguished from *P. balionotus* by having basal webbing between toes (absent in *P. balionotus*) and warty flanks (tuberculate in *P. balionotus*). *Pristimantis chomskyi* is different from *P. gloria* by having shagreen dorsal skin (tuberculate to warty in *P. gloria*), lacking a middorsal fold (present in *P. gloria*), having a larger body (males *Z* = -2.31490, *p* = 0.0206, SVL = 23.98–32.38 mm in *P. chomskyi*, 16.7–24.74 mm in *P. gloria*), a smaller tympanum (males *Z* = 2.66173, *p* = 0.0078, TD/SVL = 4.5–4.7% in *P. chomskyi*, 5.1–6.2% in *P. gloria*) and larger eye (males *Z* = -2.50743, *p* = 0.0122, ED/SVL = 11.1–12% in *P. chomskyi*, 11–13.8% in *P. gloria* sp. nov.), relative to body length. The dorsum and flanks of *Pristimantis lutzae* sp. nov. are predominantly covered with tubercles, while it is mostly covered with warts in *P. gloria*; the ratio between the length and width of the head is larger in *P. gloria* than *P. lutzae* sp. nov. (males *Z* = -5.00826, *p* < 0.0001, HL/HW = 96.8–114.5% in *P. gloria*, 90–97% in *P. lutzae* sp. nov.; females *Z* = -3.77517, *p* = 0.0002, HL/HW = 92–105% in *P. gloria*, 90–96% in *P. lutzae* sp. nov.). Morphometrically, *P. multicolor* sp. nov. is larger than *P. gloria* (males *Z* = 3.07054, *p* = 0.0021, SVL 16.7–24.7 mm in *P. gloria*, 19.7–29.7 mm in *P. multicolor* sp. nov.; females *Z* = 2.85671, *p* = 0.0043, SVL 26.7–35.8 mm in *P. gloria*, 29.3–40.5 mm in *P. multicolor* sp. nov.), and has a wider head (males *Z* = 2.23159, *p* = 0.0256, HW/SVL = 36.1–40.7% in *P. gloria*, 36.3–40.9% in *P. multicolor* sp. nov.; females *Z* = 4.13252, *p* < 0.0001, HW/SVL = 36.1–39% in *P. gloria*, 39.6–42.2% in *P. multicolor* sp. nov.) relative to body length; furthermore, *P. multicolor* sp. nov. lacks a middorsal fold. *Pristimantis percultus*, like *P. gloria*, has wide black reticulations on the iris, and warts and tubercles on flanks. They can be readily recognized because *P. percultus* has a keel on the snout (absent in *P. gloria*), cranial crests (absent in *P. gloria*), and is larger (Table 5). *Pristimantis* sp. (CCS2) is the closest species to *P. gloria* (Fig. 3, Table 2). CCS2 has a red dorsum, a golden iris with a red medial streak and thin reticulations, and broadly expanded discs on finger (Fig. 4M); while the dorsum of *P. gloria* is light to dark brown, its iris is cream to light silver with a red medial streak and wide black reticulations. Though there is only one female available for CCS2, it has a larger body and smaller tympanum than any individual of *P. gloria* (Table 5). *Pristimantis gloria* is also similar to the Peruvian species *Pristimantis pataikos* (Duellman & Pramuk, 1999) from which can be distinguished by the presence of nuptial pads in adult males (absent in *P. pataikos*), the presence of lateral fringes on fingers and toes (absent in *P. pataikos*), the presence of vomerine odontophores (absent in *P. pataikos*), and the presence of pale spots on the groins and concealed surfaces of thighs (uniformly brown in *P. pataikos*) (Duellman and Pramuk 1999).

#### Description of the holotype.

An adult female (QCAZ 57201). Measurements (in mm): SVL 35.8; TL 15.6; FL 16.5; HL 12.4; HW 13.5; ED 3.6; TD 1.8; IOD 3.5; EW 3.0; IND 2.5; EN 3.5; TED 1.7. Head wider than long, slightly narrower than body; snout moderately long, subacuminate in dorsal view, rounded in profile; cranial crests absent; nostrils slightly protuberant, narrow, directed anterolaterally; canthus rostralis slightly concave in dorsal view, angular in cross section; loreal region slightly concave; upper eyelid with indistinct tubercles, smaller than those on dorsum; tympanic annulus prominent, upper and posterolateral edge concealed by supratympanic fold; tympanic membrane distinct; two large, low and rounded postrictal tubercles surrounded by smaller tubercles. Choanae median, ovoid, not concealed by palatal shelf of maxillae; dentigerous processes of vomers prominent, large, oblique, moderately separated, positioned posteromedial to choanae; each vomer bearing several teeth; tongue slightly longer than wide, posteriorly notched, posterior half free.

Dorsal surfaces of body tuberculate, dorsum bearing median low rounded tubercles and scattered warts, more prominent posteriorly; skin on head shagreen; middorsal fold present; dorsolateral folds absent; skin on flanks bearing more prominent and larger tubercles and warts than dorsum; skin on chest and belly coarsely areolate, that on throat shagreen; discoidal fold present; ventral surfaces of limbs smooth, ventral surfaces of thighs coarsely areolate. Ulnar tubercles indistinct except for low antebrachial tubercle; outer palmar tubercle bifid, twice the size of ovoid thenar tubercle; subarticular tubercles prominent, rounded; median low supernumerary tubercles at base of fingers and palms; fingers bearing broad lateral fringes; Finger I shorter than Finger II; discs on fingers expanded and truncate; pads on fingers surrounded by circumferential grooves on all fingers (Fig. 7C).

Dorsal surfaces of hindlimbs shagreen with scattered tubercles; posterior surfaces of thighs smooth, ventral surfaces of thighs coarsely areolate; heel bearing a median, low and rounded tubercle surrounded by indistinct ones; outer edge of tarsus bearing indistinct tubercles; inner edge of tarsus bearing a median fold ending in a row of small tubercles; inner metatarsal tubercle elevated, ovoid, six times the size of round outer metatarsal tubercle; plantar surface with several small, indistinct supernumerary tubercles; subarticular tubercles prominent, rounded; toes bearing broad lateral fringes; basal webbing between toes IV and V present; discs on toes smaller than those on fingers, expanded, elliptical; all toes having pads surrounded by circumferential grooves; relative lengths of toes: I < II < III < V < IV; Toe V longer than Toe III (disc on Toe III reaches distal edge of penultimate subarticular tubercle on Toe IV, disc on Toe V reaches the middle of distal subarticular tubercle on Toe IV; Fig. 7C). Color of holotype in life and preservative is shown in Figures 15A, 16A, respectively.

#### Variation.

**Figure 17.**
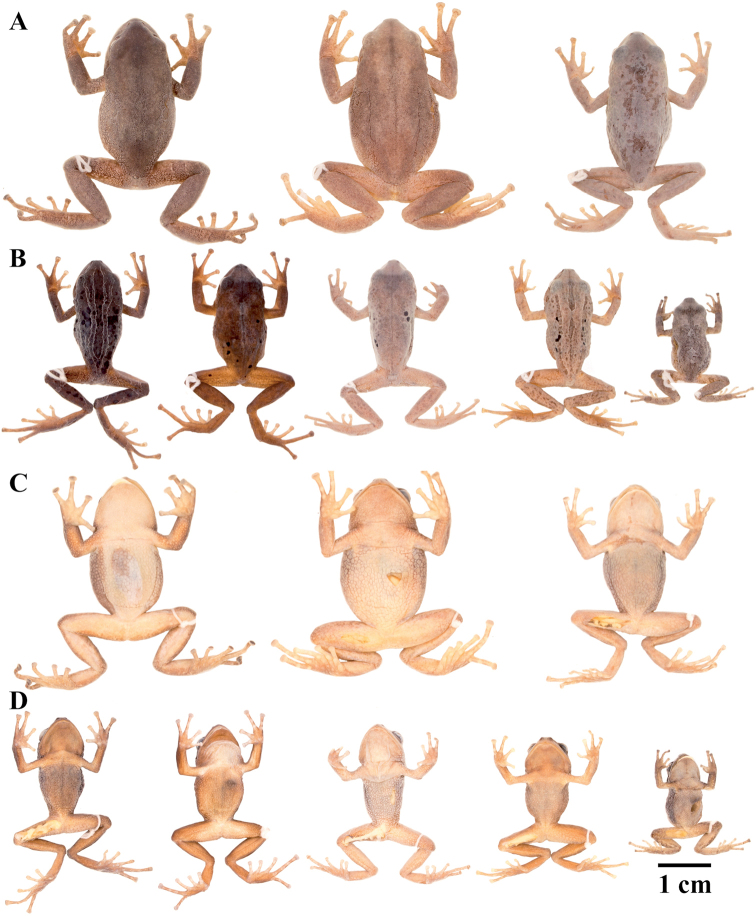
Color variation in preserved individuals of *Pristimantis gloria* sp. nov. **A** Dorsal view of (from left to right): QCAZ 31457 (female), QCAZ 31455 (female), QCAZ 16449 (female) **B** Dorsal view of: QCAZ 16448 (juvenile female), QCAZ 56463 (male), QCAZ 16452 (male), QCAZ 16447 (juvenile female), QCAZ 31461 (juvenile female) **C** Ventral view of specimens in (**A**) **D** Ventral view of specimens in (**B**). See Suppl. material 2 for locality data. All specimens are shown at the same scale.

This section is based in 60 individuals of the type series and photographs available from 15 individuals. Variation in life and preservative is shown in Figures 16, 17. In preservative, dorsal coloration varies from light to dark brown with or without shades of gray. Dorsum uniformly colored or bearing a pattern of parallel longitudinal stripes, irregular reticulations or pale blotches. Most individuals have scattered black spots on dorsum, especially on dorsolateral surfaces; three individuals (QCAZ 57201, QCAZ 26369, QCAZ30741) display scattered white spots. Black or brown canthal bars, supratympanic stripes, labial bars, and interorbital bars or stripes can be present in this species. Flanks have the same or slightly lighter hue than dorsum. Groins and posterior surfaces of thighs bear pale irregularly shaped small spots or flecks on a brown background; when flecking is very dense the background color resembles brown reticulations. One individual lacks these flecks and spots (QCAZ62778). Limbs bear or not transversal bars and black spots. Ventral coloration varies from cream to dirty cream. In life, dorsal coloration varies from light to dark brown with or without shades of cream, yellow or orange. When present, reticulations and longitudinal stripes are brighter colored than the rest of dorsum and bordered by thin black stripes. Dorsum usually bears black spots, rarely, white spots. When present, canthal and supratympanic bars or stripes are brown or black, interorbital bars or stripes are black, brown or yellow. Flanks are the same color as dorsum or lighter. Groins and posterior surfaces of thighs are brown, creamish brown, pinkish brown or purplish brown with irregular flecks or spots; when flecking is dense, the background color resembles thin reticulations (QCAZ 62778 is the only individual that do not present these spots). Venter varies from cream to brownish cream. Iris is cream or light silver with wide black reticulations that can cover almost completely the iris; a faint red to reddish brown medial streak is present (Fig. 16D); sclera varies from cream to light blue.

*Coloration of holotype in preservative*. Dorsum dark brown with abundant scattered black medium-sized spots, more concentrated on dorsolateral surfaces and a few scattered white spots; flanks slightly lighter than dorsum; black supratympanic stripes; dorsal surface of limbs with same background coloration as dorsum and scattered black spots; groins and concealed surfaces of thighs brown with cream flecks; ventral surface of body pale cream (Fig. 15A).

*Coloration of holotype in life*. Based on studio photographs (Fig. 16A). Dorsal surfaces of body grayish brown with scattered black medium-sized spots, more abundant on dorsolateral surfaces; black supratympanic stripe; few scattered white spots on dorsum and dorsal surfaces of thighs; flanks lighter than dorsum with scattered black spots; groins and concealed surfaces of thighs reddish brown with cream flecks; venter and ventral surfaces of thighs pinkish cream with cream spots coinciding with areolate pattern; throat cream; ventral surfaces of shanks, tarsus, feet and hands pinkish cream; iris silver with wide black reticulations and a reddish brown medial streak.

#### Distribution, natural history, and conservation status.

*Pristimantis gloria* is known from Inter-Andean Shrub and Paramo regions in Azuay, Loja, and Morona Santiago Provinces, between 2460 and 3525 m a.s.l. (Fig. 1). Most individuals were found underneath rocks in paramos or pastures near the roadside, during day or night. Two couples in amplexus were found underneath rocks at night in April. A calling male was found in low vegetation at 19h00 on October 16, 2013.

We consider *P. gloria* as an Endangered species following B1ab(iii) + 2ab(iii) IUCN criteria because: (i) it is only known from five localities (sensu IUCN 2017) in non-protected areas, partly affected by human settlements, agriculture and cattle raising; (ii) its Extent of Occurrence is estimated to be less than 5000 km^2^ (617 km^2^); and (iii) its Area of Occupancy < 500 km^2^ (44 km^2^).

#### Etymology.

The specific epithet is a patronym for Gloria Lorena Rosales Narváez and Gloria María Esmeralda Narváez, mother and grandmother of the leading author. This species is named after them in gratitude for all their love and support.

#### Remarks.

Because of the previous lack of molecular data, this species has been mistakenly identified as *P. riveti* (e.g., collections at the QCAZ museum). Here, we recognize it as a different species and assign it to the *P. phoxocephalus* species group. It has the lowest genetic divergence with its closest species, *Pristimantis* sp. CCS2; the average uncorrected *p* distance between them is 2%. The differences in morphology between them are so evident that we consider 2.0% as our reference value to delimit candidate species.

### Pristimantis jimenezi sp. nov.

Taxon classificationAnimaliaAnuraStrabomantidae

B6C0F4D3E44658619F71535AF5B232CB

http://zoobank.org/7968FB77-1DD1-46DE-956F-3A93E0767F34

#### Common name.

English: Jiménez de la Espada’s Rain Frog. Spanish: Cutín de Jiménez de la Espada.

#### Holotype.

**Figure 18.**
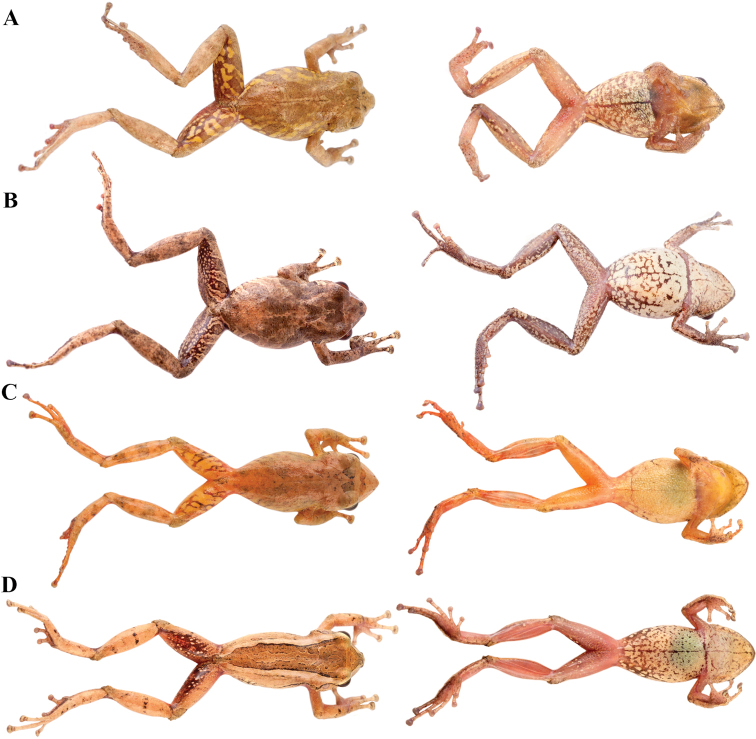
Color variation in live individuals of *Pristimantis jimenezi* sp. nov. **A**QCAZ 45170 (holotype, male, SVL 24.1 mm) **B**QCAZ 45179 (female, SVL 32.2 mm) **C**QCAZ 46977 (male, SVL 26.8 mm) **D**QCAZ 46978 (female, SVL 34.8 mm). Dorsal view on the left, ventral view on the right.

QCAZ 45170, an adult male from San Antonio, Cajas National Park border, Azuay Province, Ecuador (2.8835S, 79.4103W, 2099 m), collected by Elicio E. Tapia and Juan Carlos Morocho on August 21, 2009. Figures 15B, 18A.

#### Paratypes

**(24: 11 males, 9 females, 4 juveniles).** Ecuador: Azuay Province: QCAZ 45179, QCAZ 45186, adult females, QCAZ 45178, QCAZ 45187–189, adult males, from San Antonio, Cajas National Park border (2.8835S, 79.4103W, 2099 m), collected by Elicio E. Tapia and Juan Carlos Morocho on August 21, 2009; QCAZ 45199, adult female, QCAZ 45196, juvenile, from San Antonio, Cajas National Park border (2.8612S, 79.3787W, 2900 m), collected by Elicio E. Tapia and Juan Carlos Morocho on August 24, 2009; QCAZ 45202–203, QCAZ 45208–209, QCAZ 45212, adult females, QCAZ 45201, QCAZ 45204, QCAZ 45207, QCAZ 45211, adult males, QCAZ 45205–206, juveniles, from San Antonio, Malacatos river (2.9107S, 79.4146W, 1800 m), collected by Elicio E. Tapia and Juan Carlos Morocho on August 24, 2009; QCAZ 46955, adult male, from Chipla river (2.7457S, 79.4089W, 2500 m), collected by Elicio E. Tapia on January 16, 2010; QCAZ 46978, adult female, QCAZ 46977, QCAZ 46980, adult males, QCAZ 46979, juvenile, from Molleturo, Zadracay river (2.7353S, 79.4142W, 2674 m), collected by Elicio E. Tapia on January 20, 2010.

#### Diagnosis.

A member of the *Pristimantis phoxocephalus* group characterized by the following combination of characters: (1) skin on dorsum shagreen with or without scattered small subconical tubercles; faint middorsal fold; dorsolateral folds absent; thin lateral folds on anterior half of flanks; skin on venter areolate; discoidal fold absent; (2) tympanic membrane and tympanic annulus prominent, its upper and posterior margin concealed by supratympanic fold; (3) snout moderately long, acuminate with a fleshy keel in dorsal view, protruding in profile; (4) upper eyelid with small, distinct tubercles; cranial crests absent; (5) dentigerous processes of vomers low to prominent, oblique, moderately separated, posteromedial to choanae; (6) males bearing vocal slits and vocal sac, with white nuptial pads; (7) Finger I shorter than Finger II; discs of digits expanded to broadly expanded, elliptical to truncate; (8) fingers with broad lateral fringes; (9) ulnar tubercles low or absent; (10) heel bearing one medium subconical tubercle surrounded or not by lower tubercles; outer edge of tarsus bearing low subconical tubercles; inner edge with or without low tubercles, short inner tarsal fold present; (11) inner metatarsal tubercle ovoid, elevated, three times the size of elliptical, elevated outer metatarsal tubercle; supernumerary tubercles indistinct; (12) toes with broad lateral fringes; basal webbing present; Toe V longer than Toe III (disc on Toe III reaches or exceeds the proximal edge of penultimate subarticular tubercle on Toe IV; disc on Toe V reaches or exceeds the proximal edge of distal subarticular tubercle on Toe IV); toe discs slightly smaller than those on fingers, elliptical to truncate (Fig. 7D); (13) in life, dorsal background light to dark brown with a scapular W-shaped marking and an interorbital stripe; groins and posterior surfaces of thighs salmon, pinkish brown, purplish brown or dark brown with small light brown to yellow spots; venter cream; iris reddish copper with black reticulations with a red medial streak to completely red (Fig. 18); (14) average SVL in adult females: 35.0 ± 2.1 mm (30.9–37.4 mm; *n* = 9); in adult males: 25.5 ± 1.6 mm (21.9–27.1 mm; *n* = 12).

#### Comparison with other species.

*Pristimantis jimenezi* is similar to *P. atillo*, *P. phoxocephalus*, *P. teslai* sp. nov., *P. torresi* sp. nov., *P. totoroi* sp. nov., and *P. verrucolatus* sp. nov. It differs from *P. atillo* in the coloration of the groins and posterior surfaces of thighs (orange in *P. atillo*; pinkish to dark brown with small light brown to yellow spots in *P. jimenezi*), and flanks (black dots and flecks surrounding light reticulations in *P. atillo*; lacking black dots in *P. jimenezi*). *Pristimantis jimenezi* can be distinguished from *P. phoxocephalus* by its groin coloration (yellow with black reticulations in *P. phoxocephalus*) and advertisement call. The call of *P. phoxocephalus* has shorter inter-note intervals and a lower frequency of the second harmonic and final frequency of the note (Table 6). *Pristimantis teslai* sp. nov. has tuberculate dorsal skin (shagreen in *P. jimenezi*) and lacks lateral folds, which are present in *P. jimenezi*. The most similar species to *P. jimenezi* is *P. torresi* sp. nov., which has golden to beige iris with a red medial streak (copper to red in *P. jimenezi*). *P. totoroi* sp. nov. has more prominent folds and tubercles than *P. jimenezi*, and its head length is larger (males *Z* = 3.05006, *p* = 0.0023, HL/SVL = 33.4–37.5% in *P. jimenezi*, 34.2–38.7% in *P. totoroi* sp. nov.; females *Z* = 2.85798, *p* = 0.0043, HL/SVL = 32.8–37.3% in *P. jimenezi*, 35.2–37.6% in *P. totoroi* sp. nov.), its tympanum smaller (males *Z* = -4.24763, *p* < 0.0001, TD/SVL = 4.8–6% in *P. jimenezi*, 4.4–5.1% in *P. totoroi* sp. nov.; females *Z* = -2,96383, *p* = 0.003, TD/SVL = 5–5.6% in *P. jimenezi*, 4.8–5.3% in *P. totoroi* sp. nov.), and its eyes smaller (males *Z* = -4.06051, *p* < 0.001, ED/SVL = 11.1–13.3% in *P. jimenezi*, 9.2–11.6% in *P. totoroi* sp. nov.; females *Z* = -2.43458, *p* = 0.0149, ED/SVL = 9.5–11.8% in *P. jimenezi*, 9.5–10.6% in *P. totoroi* sp. nov.) than those of *P. jimenezi*. Furthermore, advertisement calls of *P. totoroi* sp. nov. have shorter notes and lower dominant, second harmonic, and final frequencies of the notes than those of *P. jimenezi* (Table 6). *Pristimantis verrucolatus* sp. nov., which has an adjacent distribution to *P. jimenezi* at high elevations, can be distinguished by the presence of large tubercles and warts on flanks (absent in *P. jimenezi*) and by its smaller size (males *Z* = 3.58643, *p* = 0.0003, SVL = 21.8–27.0 mm in *P. jimenezi*, 25.1–34.5 mm in *P. verrucolatus* sp. nov.; females *Z* = 2.00347, *p* = 0.0451, SVL = 31.1–37.4 mm in *P. jimenezi*, 40.4–46.8 mm in *P. verrucolatus* sp. nov.). Additionally, they can be distinguished by their advertisement calls: the call of *P. verrucolatus* sp. nov. consists of a single note, longer than the one or two notes of the call of *P. jimenezi*; the dominant frequency is higher in advertisement calls of *P. jimenezi*, and the change in frequency along the note is lower in *P. jimenezi* (Table 6).

#### Description of the holotype.

An adult male (QCAZ 45170, SC29041). Measurements (in mm): SVL 24.1; TL 12.7; FL 11.0; HL 8.2; HW 8.5; ED 2.8; TD 1.4; IOD 2.7; EW 2.5; IND 2.2; EN 2.6; TED 1.0. Body slender; head as wide as body, slightly wider than long; snout moderately long, acuminate with a vertical fleshy keel in dorsal view, protruding in profile; nostrils slightly protuberant, ovoid, directed laterally with slight dorsal inclination; canthus rostralis concave in dorsal view, angular in cross section; loreal region slightly concave, bearing small tubercles; upper eyelid bearing small distinct tubercles; cranial crests absent; tympanic membrane and annulus prominent with upper and posterior margins concealed by supratympanic fold; two medium-sized, rounded postrictal tubercles surrounded by smaller tubercles outlining supratympanic stripe. Choanae large, circular, not concealed by palatal shelf of maxilla; dentigerous processes of vomers prominent, oblique, moderately separated, posteromedial to choanae; each vomer bearing several indistinct teeth; tongue slightly wider than long, posterior border notched, posterior half free; vocal slits slightly curved, located at posterior half of mouth floor in between tongue and margin of jaw; vocal sac present.

Skin on dorsum and flanks shagreen; faint middorsal fold; row of three small middorsal tubercles on head; dorsolateral folds absent; thin lateral folds on anterior half of the flanks; skin on throat, chest, belly and ventral surfaces of thighs areolate; discoidal fold absent. One low round ulnar tubercle; white nuptial pads present; palmar tubercles prominent, outer palmar tubercle bifid, slightly bigger than ovoid thenar tubercle; subarticular tubercles distinct, rounded; low supernumerary tubercles at base of fingers; fingers with broad lateral fringes extended to outer edge of the palm; Finger I shorter than Finger II; discs on fingers expanded and elliptical; ventral pads on fingers surrounded by circumferential grooves (Fig. 7D).

Hindlimbs slender; dorsal surfaces of hindlimbs with scattered low tubercles; posterior surfaces of thighs smooth, ventral surfaces of thighs areolate; heel bearing one medium prominent subconical tubercle surrounded by few lower rounded tubercles; outer edge of tarsus bearing low subconical tubercles; short inner tarsal fold present; inner metatarsal tubercle ovoid, elevated, three times the size of elliptical, rounded outer metatarsal tubercle; plantar surface with indistinct supernumerary tubercles; subarticular tubercles distinct, rounded; toes with broad lateral fringes; basal webbing present; discs smaller than those on fingers, wider on Toe IV and V; discs on toes expanded, elliptical; all toes having ventral pads and circumferential grooves; relative lengths of toes: I < II < III < V < IV; Toe V longer than Toe III (disc on Toe III exceeds proximal edge of the distal subarticular tubercle on Toe IV, disc on Toe V exceeds proximal edge of penultimate subarticular tubercle on Toe IV; Fig. 7). Coloration of the holotype in life and preservative is shown in Figures 15B, 18A.

*Coloration of holotype in preservative*. Dorsum grayish light brown with cream markings including an irregular W-shaped scapular mark and irregular reticulations; middorsal black stripe; head with interorbital stripe; faint canthal and labial bars; dark brown supratympanic stripe; flanks with the same background color as dorsum; flanks, groins, anterior, and posterior surfaces of thighs with cream medium-sized spots and cream oblique reticulations; background color of groins, anterior, and posterior surfaces of thighs lighter than dorsum; venter cream with scattered minute brown flecks on belly and brown midventral stripe (Fig. 15B).

*Coloration of holotype in life*. Based on studio photographs. Dorsum light brown with cream markings including an irregular W-shaped scapular mark and irregular reticulations; head with interorbital stripe; faint gray canthal stripes and thin labial bars; black supratympanic stripe; flanks, groins, anterior, and posterior surfaces of thighs with yellow medium-sized spots and cream oblique reticulations; background color on groins and posterior surfaces of thighs purplish brown; ventral surfaces of limbs and chest pinkish cream; vocal sac yellowish cream; venter pinkish cream suffused with white; iris copper with thin black reticulations; light-blue sclera (Fig. 18A).

#### Variation.

**Figure 19.**
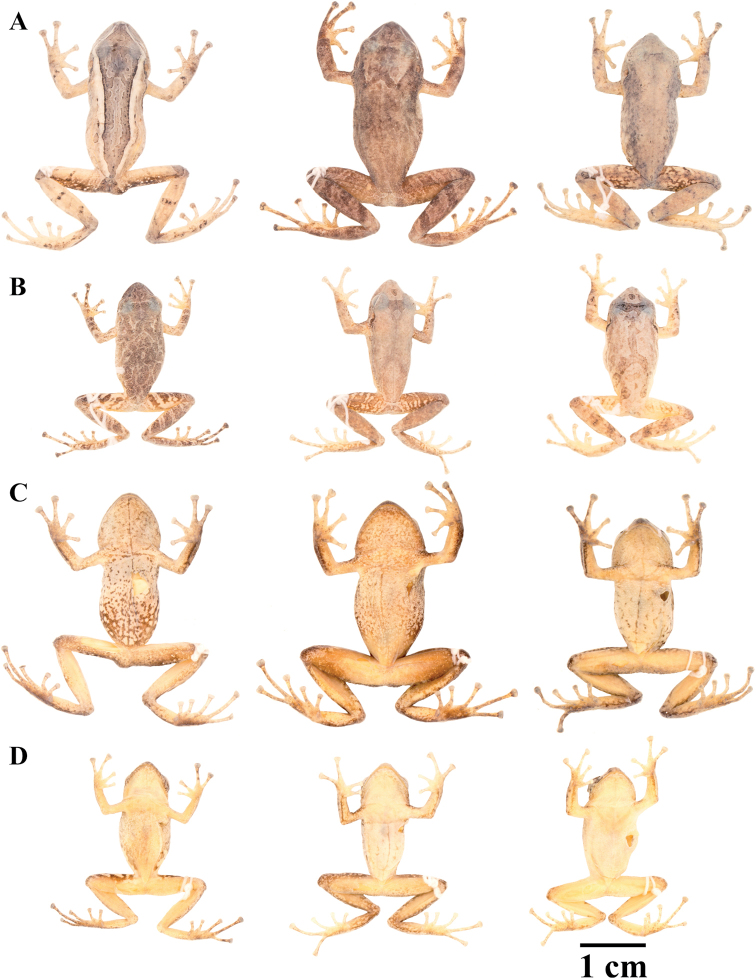
Color variation in preserved individuals of *Pristimantis jimenezi* sp. nov. **A** Dorsal view of (from left to right): QCAZ 45204 (male), QCAZ 45178 (male), QCAZ 45189 (male) **B** Dorsal view of: QCAZ 46978 (female), QCAZ 45203 (female), QCAZ 45199 (female) **C** Ventral view of specimens in (**A**) **D** Ventral view of specimens in B. Ventral view of the same specimens. See Suppl. material 2 for locality data. All specimens are shown at the same scale.

Data of preserved individuals is based on 25 specimens of the type series; information of coloration in life is based on photographs from nine individuals. Variation of preserved and live individuals is shown in Figures 18, 19. Coloration in life is provided in parenthesis. Dorsum is creamy to light and dark brown, with or without lighter irregular marks or chevrons; some individuals show a middorsal band; a pale W-shaped mark on scapula is often present; head bears an interorbital stripe or band paler or darker than dorsum; most individuals bear labial bars, and a few, canthal bars (black to brown). Flanks often bear pale (light-brown to yellow) spots or reticulations all over them or confined to the groins and axils. Groins and posterior surfaces of thighs cream with small pale spots (salmon, pinkish brown, purplish, purplish brown or dark brown with small light-brown to yellow spots). Dorsal surfaces of thighs are darker than dorsum bearing pale (yellow or light brown) reticulations. Ventral surfaces of the body are cream (yellowish to pale cream) with or without dark flecks or reticulations on the belly and throat. Iris varies from copper with a red medial streak of variable size to completely red; it bears thin black reticulations; white to light-blue sclera.

#### Advertisement call.

Based on recordings of QCAZ 45178 (January 20, 2010; 19h10; 12 °C) and QCAZ 46977 (August 23, 2009; 16h30). The advertisement call of *P. jimenezi* consists of one or two peep-like notes (Fig. 6A). The average duration of a note is 0.18 s (range 0.016–0.21 s), and the interval between them is 0.30 s (range 0.23–0.36 s). The fundamental and dominant frequencies of the notes are on average 2894 Hz (range 2776–3013 Hz). The peak time of the note occurs exactly in the middle of its duration. There is an increase in frequency along the note (2790–2977 Hz on average). Descriptive statistics for bioacoustic parameters are shown in Table 6.

#### Distribution, natural history, and conservation status.

This species is known from Western Andean slopes of Azuay Province between elevations of 1800 and 2900 m (Fig. 1); this corresponds to the Western Montane Forest region. Individuals of this species inhabit primary and secondary forest. Specimens were found at afternoon and night on low vegetation up to 1.5 m above the ground and inside bromeliads up to 8 m above the ground. Some individuals were collected from banana plants. Gravid females and calling males were collected in January and August; males were found calling at 16 and 19 h.

Following B1ab(iii) IUCN criteria, we consider *P. jimenezi* to be Critically Endangered. Available records come from two localities (sensu IUCN 2017) where suitable surrounding habitats are disturbed by human settlements, agriculture, and cattle raising. Its Extent of Occurrence is estimated to be less than 100 km^2^ (39 km^2^).

#### Etymology.

The specific epithet is a noun in the genitive case and is a patronym for Marcos Jiménez de la Espada, a Spanish naturalist who visited South America between 1862 and 1865. During his trip, he made significant collections of amphibians including new species that he described between 1870 and 1875. Among others, he named the genus *Pristimantis* and 19 species of Ecuadorian amphibians. His species descriptions are remarkably detailed in comparison with those of his contemporaries. He also made accurate and detailed descriptions of the natural history and the external and internal morphology of Neotropical anurans.

### Pristimantis lutzae sp. nov.

Taxon classificationAnimaliaAnuraStrabomantidae

F72D2FF25DC85EA194427234C92AAE1F

http://zoobank.org/F9014765-D5AC-4B9F-A04B-D1B6303059DE

#### Common name.

English: Lutz’s Rain Frog. Spanish: Cutín de Lutz.

#### Holotype.

**Figure 20.**
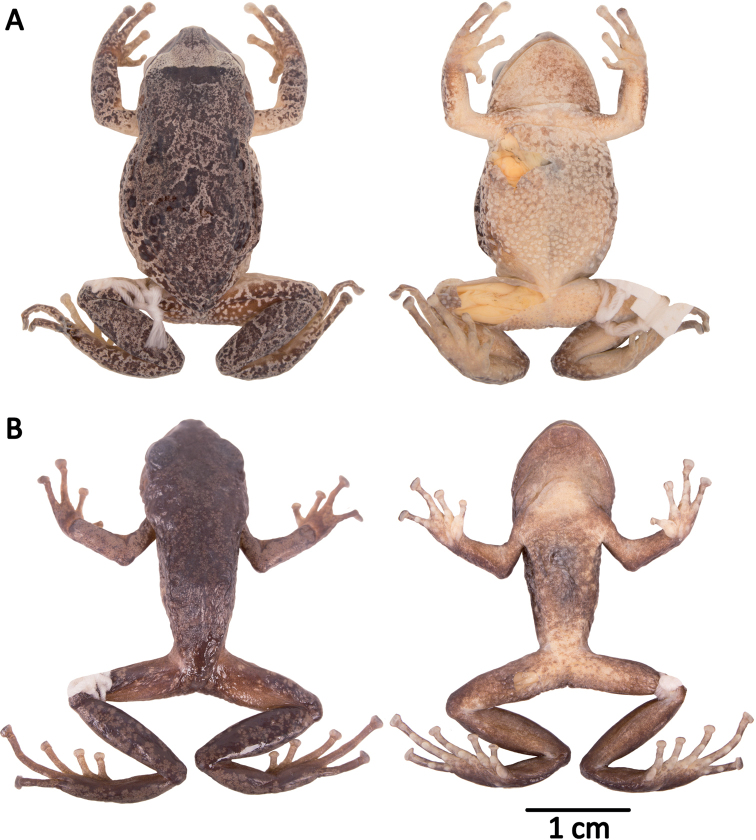
Holotypes of *Pristimantis lutzae* sp. nov. and *P multicolor* sp. nov. Photographs of preserved holotypes of **A***P. lutzae* (QCAZ 37546, female) and **B***P. multicolor* (QCAZ 47213, male). Dorsal view on the left, ventral view on the right. Specimens are shown at the same scale.

QCAZ 37546, an adult female from Mazán Reserve, Azuay Province, Ecuador (2.8689S, 79.1148W, 3047 m), collected by Luis A. Coloma, Santiago R. Ron, Ítalo G. Tapia, Ernesto Arbeláez, and Robin Moore on September 2007. Figure 20A.

#### Paratypes

**(32: 14 males, 13 females, 5 juveniles).** Ecuador: Azuay Province: QCAZ 32785, adult female, QCAZ 32786, QCAZ 32788, QCAZ 32791, adult males, QCAZ 32792, juvenile female, from Yanuncay-Irquis Protected Forest, Páramo de Quimsacocha (3.0398S, 79.2147W, 3758 m), collected by Andrés Merino-Viteri, Lorena E. Falconí, David Salazar-V, Paula Peña, Mónica Páez, Ernesto Arbeláez, and Juan Daniel Jaramillo in December 2006; QCAZ 37509, adult female, from Mazán Reserve (2.8748S, 79.1292W, 3115 m), collected by Luis A. Coloma, Santiago R. Ron, Ítalo G. Tapia, Ernesto Arbeláez and Robin Moore in September 2007; QCAZ 37545, QCAZ 37547, QCAZ 37566, adult females, QCAZ 37561, QCAZ 37564, adult males, QCAZ 37550–551, juvenile females, collected with the holotype; QCAZ 37571, adult female, QCAZ 37570, adult male, from Mazán Reserve (2.8752S, 79.1292W, 3189 m), collected by Luis A. Coloma, Santiago R. Ron, Ítalo G. Tapia, Ernesto Arbeláez and Robin Moore in September 2007; QCAZ 51736, adult female, from San Vicente (2.7953S, 78.6981W, 3044 m), collected by Omar Torres, Vanessa Aguirre, Simón Lobos, Fernando Ayala and Estefanía Boada in March 2011; QCAZ 53728, juvenile, from Cajas National Park, El Capo, Laguna Toreadora (2.7785S, 79.2453W, 4100 m), collected by Santiago R. Ron, Andrés Merino, Fernando Ayala, Teresa Camacho and Martin Cohen on July 19, 2011. Cañar Province: QCAZ 27467, QCAZ 27469, QCAZ 27534, adult females, QCAZ 27470–471, adult males, QCAZ 27472, juvenile female, from Mazar Wildlife Reserve, Rumiloma (2.5746S, 78.7455W, 3400 m), collected by Martín R. Bustamante, Joseph Mendelson and Michelle Cummer in February 2004; QCAZ 27521, adult male, from Mazar Wildlife Reserve, Rumiloma (2,5612S, 78.7336W, 3550 m), collected by Martín R. Bustamante, Joseph Mendelson and Michelle Cummer in February 2004; QCAZ 27596–597, adult females, from La Libertad (2.5466S, 78.6984W, 2895 m), collected by Martín R. Bustamante, Joseph Mendelson and Michelle Cummer in February 2004; QCAZ 47211, adult male, from Guallicanga ravine (2.4321S, 78.9022W, 3960 m), collected by Paola Mafla-Endara, Silvia Aldás-Alarcón and Freddy Velásquez-Alomoto in December 2009; QCAZ 56182, adult female, QCAZ 56183–186, adult males, from Charón Ventanas Community Tourism Center (2.6471S, 78.8905W, 3300 m), collected by Andrea Manzano, Paulina Romero, and Leonardo Negrete in July 2013.

#### Diagnosis.

**Figure 21.**
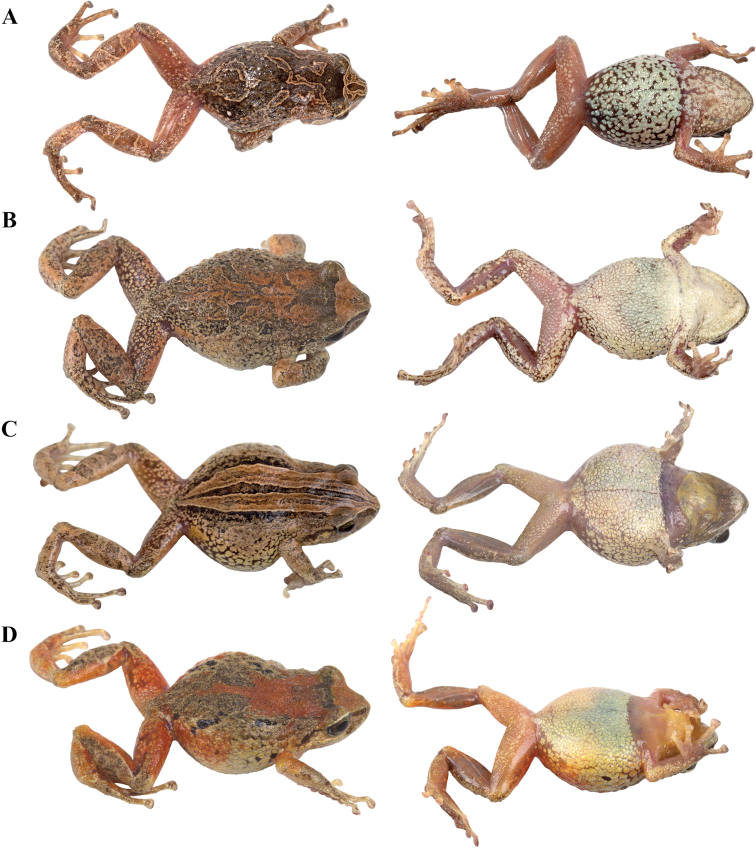
Color variation in live individuals of *Pristimantis lutzae* sp. nov. **A**QCAZ 53728 (juvenile female, SVL 26.5 mm) **B**QCAZ 56183 (male, SVL 23.3 mm) **C**QCAZ 56184 (male, SVL 24.8 mm) **D**QCAZ 56185 (male, SVL 26.1 mm). Dorsolateral view on the left, ventral view on the right.

A species of *Pristimantis* having the following combination of characters: (1) skin on dorsum shagreen to tuberculate with scattered low tubercles; thin middorsal fold present or absent; dorsolateral folds absent; flanks tuberculate, tubercles larger than those on dorsum, with or without scattered warts; lateral fold present or absent; skin on venter coarsely areolate; discoidal fold present or absent; (2) tympanic membrane and tympanic annulus prominent, its upper and posterolateral margin covered by supratympanic fold; (3) snout moderately long, round to subacuminate in dorsal, rounded in lateral view, with or without a small papilla at the tip; (4) upper eyelid bearing a small, rounded tubercle, surrounded by several lower tubercles; cranial crests absent; (5) dentigerous processes of vomers prominent, oblique, moderately separated, posteromedial to choanae; (6) vocals slits, vocal sac, and nuptial pads present in adult males; (7) Finger I shorter than Finger II; discs of digits expanded, elliptical to truncate; (8) fingers with broad lateral fringes; (9) ulnar tubercles small, distinct; (10) heel bearing a low rounded tubercle, surrounded by several smaller tubercles; inner and outer edge of tarsus bearing a row of small tubercles; short inner tarsal fold; (11) inner metatarsal tubercle elevated, ovoid, three times the size of round outer metatarsal tubercle; supernumerary tubercles indistinct; (12) toes with broad lateral fringes; basal webbing present; Toe V longer or much longer than Toe III (disc on Toe III reaches the middle of penultimate subarticular tubercle on Toe IV or slightly exceeds its distal edge, disc on Toe V reaches the middle of distal subarticular tubercle on Toe IV or slightly exceeds its distal edge); toe discs smaller than those on fingers, truncate to elliptical (Fig. 8A); (13) in life, dorsum light, orangey or dark brown; head with black or brown supratympanic, canthal, and interorbital stripes; groins, anterior and posterior surfaces of thighs pinkish, purplish or reddish brown, suffused or not with orange, with cream or light brown spots; ventral surfaces of body white to cream with or without brown reticulations; vocal sac in males yellow; iris golden to creamy brown with a reddish brown medial streak (Fig. 21); (14) average SVL in adult females: 35.3 ± 3.5 mm (29.7–33.9 mm; *n* = 15); in adult males: 24.6 ± 1.7 mm (21.4–27.0 mm; *n* = 14).

#### Comparison with other species.

*Pristimantis lutzae* is similar to *P. balionotus*, *P. chomskyi*, *P. gloria*, *P. multicolor* sp. nov., and *P. philipi*. The most similar is *P. balionotus*, which occurs at lower elevations and can be recognized by the absence of basal webbing between toes (present in *P. lutzae*) and having smaller discs on fingers. *Pristimantis lutzae* can be distinguished from *P. chomskyi* by the golden to creamy brown iris with a reddish dark brown streak (orange with a faint reddish brown streak in *P. chomskyi*), and having a bigger tympanum (males *Z* = 2.45677, *p* = 0.0140, TD/SVL = 4.5–4.7% in *P. chomskyi*, 5–5.4% in *P. lutzae*). *Pristimantis gloria* differs from *P. lutzae* in having a wartier skin, wide black reticulations on iris (thin in *P. lutzae*), and a larger ratio between the length and width of the head (males *Z* = -5.00826, *p* < 0.0001, HL/HW = 96.8–114.5% in *P. gloria*, 90–97% in *P. lutzae*; females *Z* = -3.77517, *p* = 0.0002, HL/HW = 92–105% in *P. gloria*, 90–96% in *P. lutzae*). *Pristimantis multicolor* sp. nov. has a longer head (males *Z* = 3.67756, *p* = 0.0002, HL/SVL = 33.4–37% in *P. lutzae*, 34.1–40.4% in *P. multicolor* sp. nov.; females *Z* = 3.9524, *p* < 0.0001, HL/SVL = 35–37.5% in *P. lutzae*, 36.5–40.6% in *P. multicolor* sp. nov.), larger tympanum (males *Z* = 3.57469, *p* = 0.0004, TD/SVL = 5–5.4% in *P. lutzae*, 4.9–6% in *P. multicolor* sp. nov.; females *Z* = 3.9524, *p* < 0.0001, TD/SVL = 4.9–5.6% in *P. lutzae*, 5.5–6.7% in *P. multicolor* sp. nov.) and larger eyes (males *Z* = 2.75174, *p* = 0.0059, ED/SVL = 10.3–12.1% in *P. lutzae*, 10.3–13.2% in *P. multicolor* sp. nov.; females *Z* = 3.3083, *p* = 0.0009, ED/SVL = 9.9–12.1% in *P. lutzae*, 10.7–12.4% in *P. multicolor* sp. nov.), relative to the body length, than *P. lutzae*. *Pristimantis lutzae* is readily distinguished from *P. philipi* because it has a visible tympanic membrane and annulus, and its males have vocal slits (absent traits in *P. philipi*).

#### Description of the holotype.

An adult female (QCAZ 37546, SC 21029). Measurements (in mm): SVL 31.7; TL 13.7; FL 14.7; HL 11.2; HW 12.1; ED 3.7; TD 1.7; IOD 3.8; EW 2.8; IND 2.3; EN 3.5; TED 1.3. Head wider than long, slightly narrower than body; snout moderately long, rounded with a small papilla in dorsal and lateral view; cranial crests absent; nostrils slightly protuberant, narrow, directed laterally with slight dorsal inclination; canthus rostralis slightly concave in dorsal view, rounded in cross section; loreal region slightly concave; upper eyelid bearing a small, low and rounded tubercle surrounded by inconspicuous tubercles; tympanic annulus prominent, its upper and posterior margins concealed by supratympanic fold; tympanic membrane distinct; two low and rounded postrictal tubercles surrounded by lower tubercles. Choanae large, round, not concealed by palatal shelf of maxillae; dentigerous processes of vomers prominent, oblique, moderately separated, positioned posteromedial to choanae; each vomer bearing several teeth; tongue longer than wide, posterior border notched, posterior half free.

Dorsum shagreen with scattered low tubercles, larger posteriorly; dorsolateral folds absent; skin on flanks bearing low rounded tubercles, larger than those on dorsum; skin on chest and belly coarsely areolate, that on throat shagreen, ventral surfaces of limbs smooth, ventral surfaces of thighs coarsely areolate; discoidal fold present. Ulnar tubercles rounded and low; outer palmar tubercle bifid, three times the size of ovoid thenar tubercle; subarticular tubercles prominent, rounded; low supernumerary tubercles; fingers bearing broad lateral fringes; Finger I shorter than Finger II; discs on fingers expanded and truncate; pads on fingers surrounded by circumferential grooves on all fingers (Fig. 8A).

Dorsal surfaces of hindlimbs shagreen with scattered small tubercles; posterior surfaces of thighs smooth, ventral surfaces of thighs coarsely areolate; heel bearing a median, low, rounded tubercle surrounded by smaller ones; outer and inner edge of tarsus bearing a row of small tubercles; small inner tarsal fold present; inner metatarsal tubercle ovoid, elevated, three times the size of round outer metatarsal tubercle; plantar surface with small, indistinct supernumerary tubercles; subarticular tubercles prominent, rounded; toes bearing broad lateral fringes; basal webbing between Toes IV and V present; discs on toes smaller than those on fingers, slightly expanded, truncate; all toes having pads surrounded by circumferential grooves; relative lengths of toes: I < II < III < V < IV; Toe V much longer than Toe III (disc on Toe III reaches the distal edge of penultimate subarticular tubercle on Toe IV, disc on Toe V reaches the distal edge of distal subarticular tubercle on Toe IV; Fig. 8A). Coloration of the holotype in preservative is shown in Figure 20A; coloration in life, unknown.

*Coloration of holotype in preservative*. Dorsum grayish dark brown with lighter irregular reticulations and scattered black spots; dark brown supratympanic stripe, canthal, and interorbital bands; dorsal surfaces of limbs with the same background as dorsum and darker irregular transversal bands and scattered dark brown spots; groins, anterior, and posterior surfaces of thighs reddish brown with cream small spots; ventral surfaces of body cream; soles and palms dusty cream (Fig. 20A).

*Coloration of holotype in life*. Unknown.

#### Variation.

**Figure 22.**
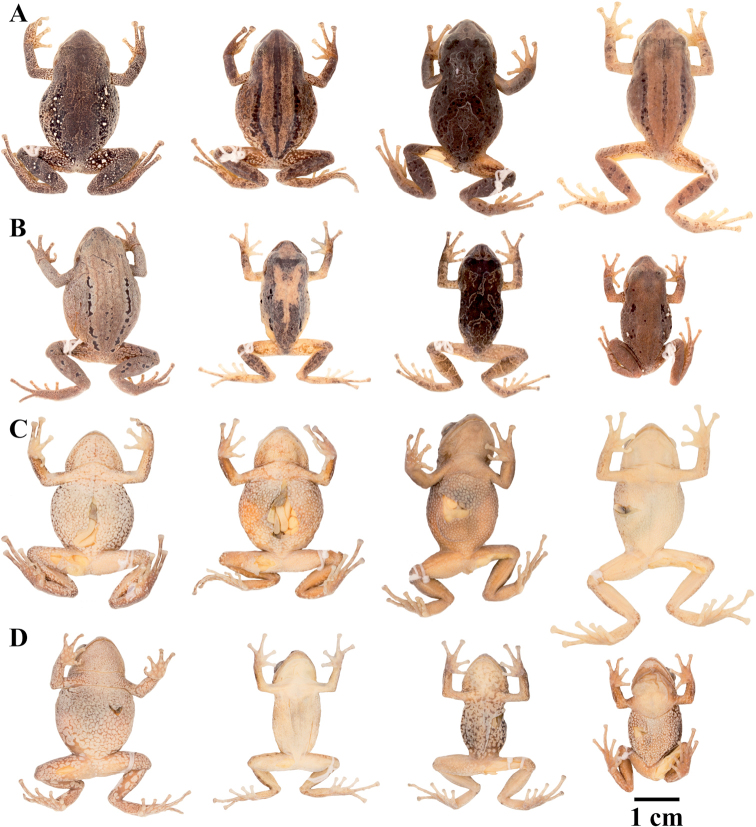
Color variation in preserved individuals of *Pristimantis lutzae* sp. nov. **A** Dorsal view of (from left to right): QCAZ 37545 (female), QCAZ 37547 (female), QCAZ 27534 (female), QCAZ 27597 (female) **B** Dorsal view of: QCAZ 32785 (female), QCAZ 56185 (male), QCAZ 53728 (juvenile female), QCAZ 27471 (male) **C** Ventral view of specimens in (**A**) **D** Ventral view of specimens in B. See Suppl. material 2 for locality data. All specimens are shown at the same scale.

Variation in preservative is based on 40 individuals of the type series and photographs from eight individuals. Variation in life and preservative is shown in Figures 21, 22. Coloration in life is provided in parenthesis. Dorsal coloration varies from light to dark gray or brown (light, orangey or dark brown); markings on dorsum (light brown, yellow or orange) are present or absent, most individuals have irregular reticulations, some have a series of parallel longitudinal stripes; dorsum and flanks may bear scattered black or white spots. All individuals bear supratympanic and canthal stripes (black, brown); an interorbital stripe or band is present except for individuals with the longitudinal pattern on dorsum. Flanks have the same background color as dorsum. Groins, posterior and anterior surfaces of thighs are cream to brown with small paler spots (pinkish, purplish or reddish brown, suffused or not with orange, with cream or light brown spots). Limbs bear dark transversal bands or scattered small dark spots. Ventral coloration varies from cream to dusty cream (white to cream); venter with or without brown reticulations. The iris is golden to creamy brown with a reddish dark brown medial horizontal streak. White to light-blue sclera.

#### Distribution, natural history, and conservation status.

*Pristimantis lutzae* is known from Paramo, Inter-Andean Shrub, Western and Eastern Montane Forest in the Andes of Azuay and Cañar Provinces in Ecuador, between 2895–4100 m a.s.l (Fig. 1). Individuals collected at night were found in bunch grasses, pastures, and low vegetation up to 80 cm above the ground. Individuals collected at day were found in pastures or underneath rocks. Calling males have been found on bunch grasses or low vegetation during February, July, and December at night.

Despite the relatively small distribution range of this species (Extent of Occurrence = 2338 km^2^) we assign it to the Least Concern Red List category because its distribution overlaps with four protected areas, Cajas National Park, Mazán Reserve, Mazar Wildlife Reserve, and Yanuncay Irquis Protected Forest, and it is a common species in these places.

#### Etymology.

The specific epithet is a noun in the genitive case and is a patronym for Bertha Lutz, who was a Brazilian herpetologist. We name this species after her in recognition of her scientific career and her activism in the fight for gender equality.

#### Remarks.

**Figure 23.**
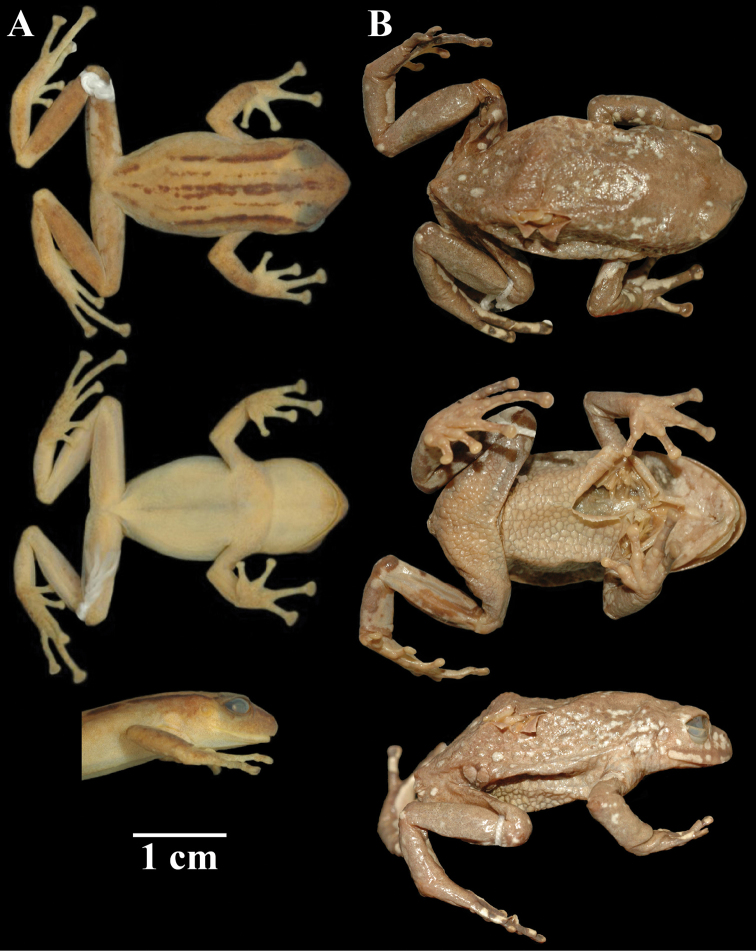
Preserved holotypes of *Pristimantis phoxocephalus* and *P. riveti*. Dorsal, ventral and lateral views of: **A***Pristimantis phoxocephalus* (KU 142075) **B***Pristimantis riveti* (MNHNP 1902.357). Shown at the same scale.

Specimens of this species were previously referred as *Pristimantis riveti* (Despax 2011) based on Lynch (1979) characterization of the species (e.g., Almendáriz and Orcés 2006; Heinicke et al. 2007; Padial et al. 2014). Photographs of the holotype of *P. riveti* (Fig. 23B) as well as the location of its type locality indicate that *P. lutzae* is a different species. See Taxonomic status of *P. riveti* section for details. Through morphological and molecular evidence, we recognize *P. lutzae* as a different species and assign it to the *P. phoxocephalus* species group.

### Pristimantis multicolor sp. nov.

Taxon classificationAnimaliaAnuraStrabomantidae

B59FBA030F035F5B83BC88BD3351F454

http://zoobank.org/8F325BAA-C366-4F73-B767-A19551E5A7F8

#### Common name.

English: Multicolored Rain Frog. Spanish: Cutín multicolor.

#### Holotype.

QCAZ 47213, an adult male from Yacuri National Park, Laguna Negra, Loja Province, Ecuador (4.7081S, 79.4322W, 3400 m), collected by Carlos Antonio Rodríguez on December 10, 2009. Figure 20B.

#### Paratypes

**(59: 35 males, 23 females, 1 juvenile).** All individuals from Yacuri National Park, nearby the type locality. Ecuador: Loja Province: QCAZ 47214, adult female, collected with the holotype; QCAZ 47215, adult male, from Laguna Negra (4.7095S, 79.4356W, 3348 m), collected by Carlos Antonio Rodríguez on December 10, 2009; QCAZ 61279, QCAZ 61390–391, QCAZ 61404, adult females, QCAZ 61392, QCAZ 61394, QCAZ 61396, adult males, from Jimbura-San Andrés road (4.7325S, 79.4352W, 3300 m); QCAZ 61283, QCAZ 61287, adult females, QCAZ 61284, QCAZ 61286, QCAZ 61297, adult males, from Laguna Negra (4.7121S, 79.4308W, 3186 m); QCAZ 61293, adult male, from Loja-Zamora border (4.7436S, 79.4234W, 3515 m); QCAZ 61299–300, QCAZ 61302–304, adult females, QCAZ 61301, QCAZ 61306, adult males, from entrance to Lagunas Coloradas, Jimbura-San Andrés road (4.7349S, 79.4283W, 3390 m); QCAZ 61310, adult female, QCAZ 61314, QCAZ 61316–317, adult males, from Laguna de los Patos (4.7204S, 79.4286W, 3247 m); QCAZ 61344, QCAZ 61346, QCAZ 61348, adult females, QCAZ 61343, adult male, from Loja-Zamora border (4.7433S, 79.4244W, 3506 m); QCAZ 61349, QCAZ 61352, QCAZ 61361, adult females, QCAZ 61362, QCAZ 61367–368, adult males, QCAZ 61351, juvenile, from Jimbura-San Andrés road (4.7277S, 79.4333W, 3411 m); QCAZ 61358, adult male, from surroundings of refuge (4.7126S, 79.4399W, 3245 m); QCAZ 61359, QCAZ 61363, QCAZ 61369–370, adult males, from Los Picachus (4.7121S, 79.4308W, 3186 m); QCAZ 61373, adult female, from Laguna Negra (4.7154S, 79.4212W, 3242 m); QCAZ 61377, QCAZ 61379–380, adult females, from Jimbura-Zumba road (4.7252S, 79.4353W, 3333 m); QCAZ 61383–384, adult females, QCAZ 61381, adult male, from Jimbura-San Andrés road (4.7309S, 79.4340W, 3332 m), collected by Francy Mora, Keyko Cruz, David Velalcázar, Andrés Calahorrano, Darwin Núñez, Juan Sánchez, Javier Pinto and Daniel Rivadeneira between April 21 and May 2, 2015. Zamora Chinchipe Province: QCAZ 61407, QCAZ 61424–425, adult females, QCAZ 61399, QCAZ 61410, QCAZ 61413, QCAZ 61427, QCAZ 61432, QCAZ 61435–436, adult males, from Laguna Colorada (4.7379S, 79.3958W, 3318 m), collected by Francy Mora, Keyko Cruz, David Velalcázar, Andrés Calahorrano, Darwin Núñez, Juan Sánchez, Javier Pinto and Daniel Rivadeneira between April 21 and May 2, 2015.

#### Diagnosis.

**Figure 24.**
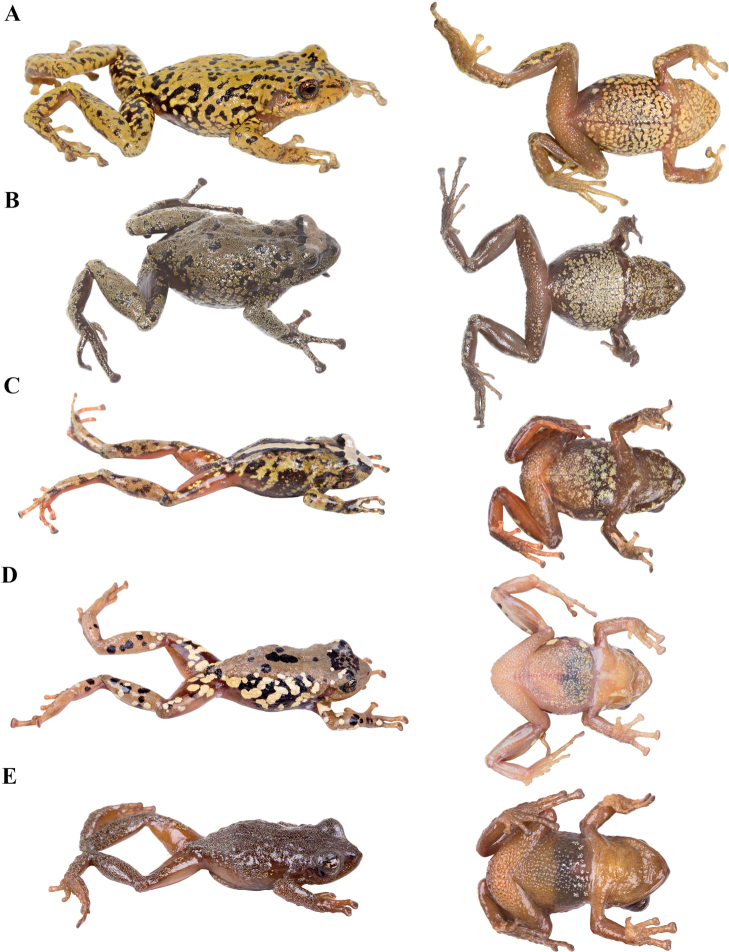
Color variation in live individuals of *Pristimantis multicolor* sp. nov. **A**QCAZ 61283 (female, SVL 32 mm) **B**QCAZ 61304 (female, SVL 34.6 mm) **C**QCAZ 61373 (male, SVL 21.8 mm) **D**QCAZ 61381 (male, SVL 29.2 mm) **E**QCAZ 61418 (male, SVL 24.5 mm). Dorsolateral view on the left, ventral view on the right.

A species of *Pristimantis* having the following combination of characters: (1) skin on dorsum shagreen to warty; dorsolateral folds absent; skin on flanks bearing low warts, bigger than those of dorsum; skin on venter coarsely areolate; discoidal fold absent; (2) tympanic membrane and tympanic annulus prominent, its upper and posterior margin concealed by thick supratympanic fold; (3) snout moderately long, subacuminate in dorsal view, rounded in profile, with or without a papilla at the tip; (4) upper eyelid lacking tubercles; cranial crests absent; (5) dentigerous processes of vomers low to prominent, oblique, narrowly to moderately separated, posteromedial to choanae; (6) vocals slits, vocal sac, and nuptial pads present in adult males; (7) Finger I shorter than Finger II; discs of digits expanded to broadly expanded, elliptical to truncate; (8) fingers with lateral fringes; (9) ulnar tubercles present; (10) heel bearing one rounded tubercle, surrounded or not by several smaller tubercles; inner and outer edge of tarsus bearing small and low tubercles; (11) inner metatarsal tubercle ovoid, elevated six times the size of elliptical, elevated outer metatarsal tubercle; supernumerary tubercles numerous; (12) toes with lateral fringes; basal webbing present; Toe V much longer than Toe III (disc on Toe III reaches or exceeds distal edge of penultimate subarticular tubercle on Toe IV, disc on Toe V reaches or exceeds distal edge of distal subarticular tubercle on Toe IV); toe discs smaller than those on fingers, elliptical (Fig. 8B); (13) in life, dorsal surfaces vary from yellow to dark brown with or without olive or orange tones; black or brown canthal and supratympanic stripe usually present; groins and posterior surfaces of thighs cream, orange, brown, or black with or without cream to yellow spots; venter varies from cream to dark brown with a few to many small beige to yellow spots; iris copper to creamy yellow with or without a dark brown to red medial horizontal streak, with black reticulations (Fig. 24); (14) average SVL in adult females: 35.3 ± 3.5 mm (29.4–40.5 mm; *n* = 27); in adult males: 26.2 ± 3.5 mm (19.7–29.7 mm; *n* = 32).

#### Comparison with other species.

It is most similar to *P. balionotus*, *P. chomskyi*, *P. gloria*, *P. lutzae*, and *P. percultus*. *Pristimantis multicolor* is different from *P. balionotus* by having basal webbing between toes (absent in *P. balionotus*), larger discs on fingers and toes, and larger tubercles and warts on flanks. Morphometrically, *P. chomskyi* has a smaller tympanum (males *Z* = 2.38157, *p* = 0.0172, TD/SVL = 4.5–4.7% in *P. chomskyi*, 4.9–6.0% in *P. multicolor*). *Pristimantis multicolor* differs from *P. gloria* by its larger size (males *Z* = 3.07054, *p* = 0.0021, SVL 16.7–24.7 mm in *P. gloria*, 19.7–29.7 mm in *P. multicolor*; females *Z* = 2.85671, *p* = 0.0043, SVL 26.7–35.8 mm in *P. gloria*, 29.3–40.5 mm in *P. multicolor*), by lacking a middorsal longitudinal fold (present in *P. gloria*), and having thinner black reticulations on the iris; additionally, *P. multicolor* has a proportionally wider head (males *Z* = 2.23159, *p* = 0.0256, HW/SVL = 36.1–40.7% in *P. gloria*, 36.3–40.9% in *P. multicolor*; females *Z* = 4.13252, *p* < 0.0001, HW/SVL = 36.1–39% in *P. gloria*, 39.6–42.2% in *P. multicolor*). Compared to *P. lutzae*, *Pristimantis multicolor* sp. nov. has a longer head (males *Z* = 3.67756, *p* = 0.0002, HL/SVL = 33.4–37% in *P. lutzae*, 34.1–40.4% in *P. multicolor*; females *Z* 3.9524, *p* < 0.0001, HL/SVL = 35–37.5% in *P. lutzae*, 36.5–40.6% in *P. multicolor*), larger tympanum (males *Z* = 3.57469, *p* = 0.0004, TD/SVL = 5–5.4% in *P. lutzae*, 4.9–6% in *P. multicolor*; females *Z* = 3.9524, *p* < 0.0001, TD/SVL = 4.9–5.6% in *P. lutzae*, 5.5–6.7% in *P. multicolor*) and larger eyes (males *Z* = 2.75174, *p* = 0.0059, ED/SVL = 10.3–12.1% in *P. lutzae*, 10.3–13.2% in *P. multicolor*; females *Z* = 3.3083, *p* = 0.0009, ED/SVL = 9.9–12.1% in *P. lutzae*, 10.7–12.4% in *P. multicolor*), relative to body size. *Pristimantis percultus* is easily distinguished from *P. multicolor* by having low cranial crests (absent in *P. multicolor*) and a red stripe on the upper lip.

#### Description of the holotype.

An adult male (QCAZ 47213, SC29908). Measurements (in mm): SVL 29.3; TL 14.8; FL 14.5; HL 11.0; HW 11.5; ED 3.5; TD 1.8; IOD 3.7; EW 2.8; IND 2.5; EN 3.1; TED 1.9. Head wider than long, wider than body; snout moderately long, subacuminate in dorsal view, rounded in profile; cranial crests absent; nostrils slightly protuberant, directed anterolaterally; canthus rostralis concave in dorsal view, prominent and rounded in cross section; loreal region concave; upper eyelid with indistinct tubercles; tympanic membrane distinct; tympanic annulus prominent, upper and posterior edge concealed by thick, heavy supratympanic fold; three large rounded postrictal tubercles. Choanae median ovoid, not concealed by palatal shelf of maxillae; dentigerous processes of vomers prominent, oblique, moderately separated, positioned posteromedial to choanae; each vomer bearing several teeth; tongue slightly longer than wide, posterior border notched, posterior three fifths not adherent to floor of mouth; vocal slits slightly curved, located at posterior half of mouth floor in between tongue and margin of jaw; vocal sac present.

Dorsal surfaces of body shagreen with scattered low warts; dorsolateral folds absent; skin on flanks bearing rounded warts, larger than those on dorsum; skin on venter coarsely areolate, ventral surfaces of limbs smooth, ventral surfaces of thighs coarsely areolate; discoidal fold absent. Low, round ulnar tubercles, antebrachial tubercle prominent; white nuptial pads present; outer palmar tubercle bifid, almost twice the size of ovoid thenar tubercle; subarticular tubercles prominent, rounded; ill-defined supernumerary tubercles; fingers bearing lateral fringes; Finger I shorter than Finger II; discs on fingers broadly expanded, truncate; pads on fingers surrounded by circumferential grooves on all fingers (Fig. 8B).

Hindlimbs slender; dorsal surfaces of hindlimbs shagreen; posterior surfaces of thighs smooth, ventral surfaces of thighs coarsely areolate; heel bearing a medium sized, low, rounded tubercle surrounded by several smaller tubercles; outer and inner edge of tarsus bearing low rounded tubercles; inner metatarsal tubercle ovoid, elevated, six times the size of elliptical, elevated outer metatarsal tubercle; plantar surface bearing numerous ill-defined supernumerary tubercles; subarticular tubercles prominent, subacuminate; toes bearing lateral fringes; basal webbing between toes IV and V present; discs on toes smaller than those on fingers, expanded and elliptical; all toes having pads surrounded by circumferential grooves; relative lengths of toes: I < II < III < V < IV; Toe V much longer than Toe III (disc on Toe III reaches distal edge of penultimate subarticular tubercle on Toe IV, disc on Toe V reaches the distal edge of distal subarticular tubercle on Toe IV; Fig. 8B). Coloration of the holotype in preservative is shown in Figure 20B; coloration in life, unknown.

*Coloration of holotype in preservative*. Dorsum dark brown covered with minute light brown irregular spots; dorsal surfaces of forelimbs light brown with scattered minute dark brown flecking; dorsal surfaces of hindlimbs dark brown with light brown spots becoming larger and more abundant posteriorly; groins and concealed surfaces of thighs, shanks and tarsus brown; ventral surfaces of body dusty brown; chest with cream irregular blotches; vocal sac and ventral surfaces of thighs cream; ventral surfaces of fingers and toes pale cream (Fig. 20B).

*Coloration of holotype in life*. Unknown.

#### Variation.

**Figure 25.**
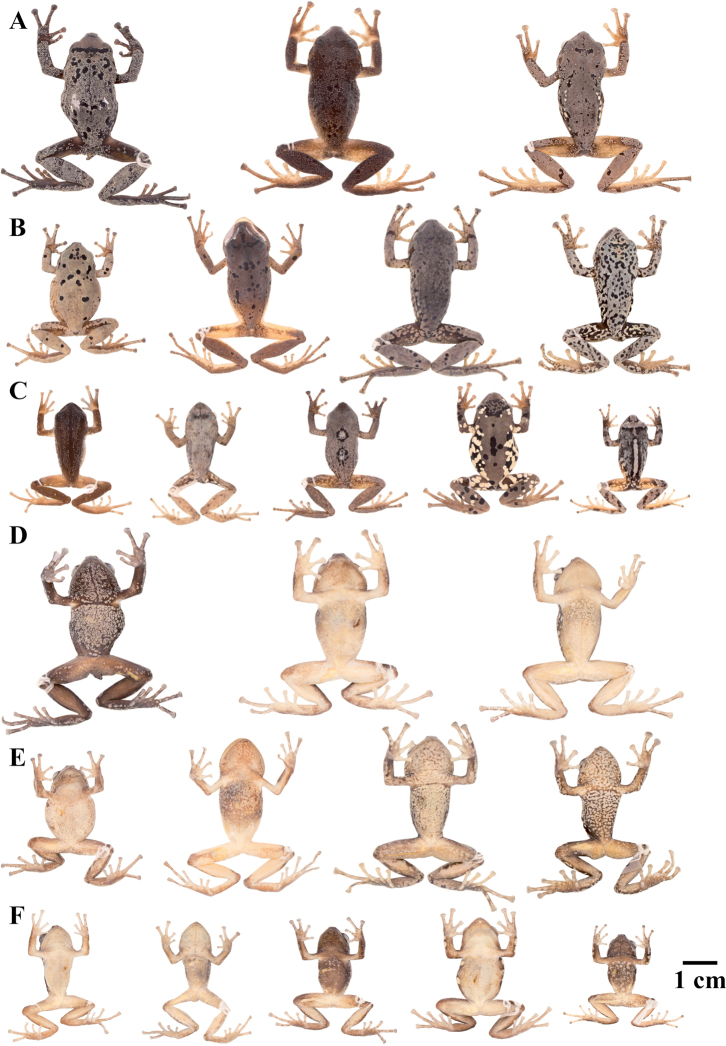
Color variation in preserved individuals of *Pristimantis multicolor* sp. nov. **A** Dorsal view of (from left to right): QCAZ 61304 (female), QCAZ 61361 (female), QCAZ 61383 (female) **B** Dorsal view of: QCAZ 61394 (male), QCAZ 47214 (female), QCAZ 61310 (female), QCAZ 61283 (female) **C** Dorsal view of: QCAZ 61413 (male), QCAZ 61297 (male), QCAZ 61369 (male), QCAZ 61381 (male), QCAZ 61373 (male) **D** Ventral view of specimens in (**A**) **E** Ventral view of specimens in (**B**) **F** Ventral view of specimens in (**C**). See Suppl. material 2 for locality data. All specimens are shown at the same scale.

Based on 60 preserved specimens and photographs from 57 individuals. Variation of live and preserved individuals is shown in Figures 24, 25. Texture of skin varies among individuals. Dorsum varies from shagreen to warty, with very low warts (not tuberculate); flanks vary from tuberculate to warty, warts on flanks are larger than those of dorsum; tubercles on ulna, shanks, and tarsus can be distinct or inconspicuous. Coloration in life is highly variable (Fig. 24). Dorsal surfaces can be yellow, cream, orange, or pale to dark brown with or without olive or orange tones. They can have few to abundant black spots on dorsum, flanks, and dorsal surfaces of limbs. Dorsum occasionally bears pale scapular or sacral blotches, a middorsal stripe, or white spots. Head usually bears black or brown canthal and supratympanic stripes, sometimes an interorbital bar. Groins and posterior surfaces of thighs are cream, orange, reddish brown, orangey brown, brown, dark brown, or black with or without cream to yellow spots. The background color of the venter varies from cream to dark brown with different levels of transparency; small beige to yellow blotches can be present on the belly, chest, and throat, but in some individuals are restricted to the chest. Iris is copper, golden, creamy yellow or creamy brown with or without a dark brown to red medial horizontal streak; black reticulations are present; sclera can be light brown, cream, white, or light blue.

#### Distribution, natural history, and conservation status.

*Pristimantis multicolor* is known from Parque Nacional Yacuri, near the border with Peru, at Loja and Zamora Chinchipe Provinces (Fig. 1). It inhabits Paramo and Eastern Montane Forest between 3186 and 3515 m. During the late afternoon and night, individuals were commonly found beneath or over moss, mat-forming plants, tussock grasses and rocks, perched on branches and leaves of shrubs up to 1.75 m above ground, or inside bromeliads, terrestrial or epiphytes, up to 1 m above the ground. During the day they were found inside bromeliads. Calling males and gravid females were found in December, April, and May; males were calling on low branches of shrubs.

According to available data, *P. multicolor* has a very restricted distribution (Extent of Occurrence = 10 km^2^) but is locally abundant. However, we propose assigning it to the Data Deficient Red List Category (IUCN 2017) because extensive areas in Yacuri National Park remain unexplored and represent potential distribution for this species.

#### Etymology.

The specific epithet comes from the Latin words *multus* meaning many, and *color* meaning color. It refers to the wide range of color variation in this species (Fig. 24).

#### Remarks.

This species was collected for the first time in 2015 by personnel from the QCAZ museum during expeditions to Yacuri National Park. It was identified as Pristimantis aff. riveti. Here, we recognize it as a different species and assign it to the *P. phoxocephalus* species group.

### Pristimantis phoxocephalus

Taxon classificationAnimaliaAnuraStrabomantidae

(Lynch, 1979)

B9F89C8F7B745416A627F30BCFF4654B

Eleutherodactylus phoxocephalus Lynch 1979. Holotype KU 142075. Figure 23A.
Pristimantis phoxocephalus Heinicke et al. 2007

#### Common name.

English: Cotopaxi Rain Frog. Spanish: Cutín silbador.

#### Diagnosis.

**Figure 26.**
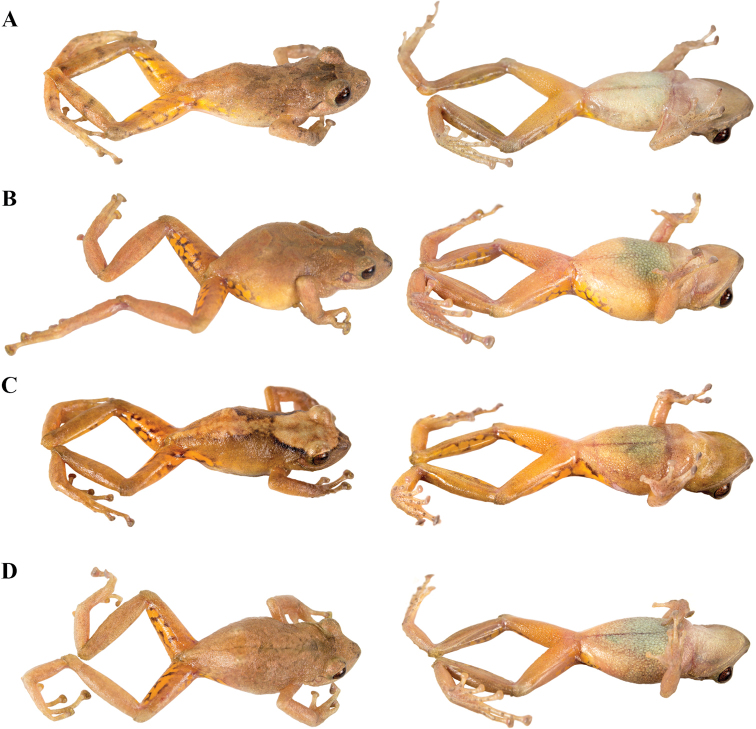
Color variation in live individuals of *Pristimantis phoxocephalus*. **A**QCAZ 58461 (male, SVL 29.9 mm) **B**QCAZ 58463 (female, SVL 36.1 mm) **C**QCAZ 58470 (male, SVL 26.7 mm) **D**QCAZ 58472 (female, SVL 34.2 mm). Dorsolateral view on the left, ventral view on the right.

This and the following sections are based on specimens of *P. phoxocephalus* listed in Suppl. material 2. A species of the *Pristimantis phoxocephalus* group having the following combination of characters: (1) dorsum shagreen with scattered small tubercles; faint middorsal fold; head with a middorsal row of two or more small tubercles; dorsolateral folds absent; anterior half of flanks with or without inconspicuous longitudinal lateral folds; skin on venter areolate to coarsely areolate; discoidal fold present or absent; (2) tympanic membrane and tympanic annulus prominent, its upper and posterolateral margin covered by supratympanic fold; (3) snout moderately long, acuminate with a fleshy keel in dorsal view, protruding in profile; (4) upper eyelid with small distinct rounded tubercles; cranial crests absent; (5) dentigerous processes of vomers low to prominent, oblique, moderately separated, posteromedial to choanae; (6) vocals slits, vocal sac and large nuptial pads present in adult males; (7) Finger I shorter than Finger II; discs of digits broadly expanded, rounded to elliptical; (8) fingers with lateral fringes; (9) small distinct ulnar tubercles (Fig. 23A); (10) heel bearing a small, subconical tubercle surrounded by smaller tubercles; outer edge of tarsus bearing a row of low subconical tubercles; inner edge of tarsus bearing a small inconspicuous fold followed by small subconical tubercles; (11) inner metatarsal ovoid, elevated, 4–6 times the size of round outer metatarsal tubercle; supernumerary tubercles low, numerous; (12) toes with broad lateral fringes; basal webbing between toes present; Toe V longer or much longer than Toe III (disc on Toe III reaches the middle to distal edge of penultimate subarticular tubercle on Toe IV, disc on Toe V reaches the middle to distal edge of distal subarticular tubercle on Toe IV); toe discs smaller than those on fingers, rounded to elliptical (Fig. 8D); (13) in life, dorsal coloration is cream, yellow, or brown with or without reddish or olive tones; dorsum with or without a longitudinal middorsal band, scapular W-shaped marking, chevrons, white or black flecking or pale botches; head with or without black or brown interorbital band, canthal stripe, supratympanic stripes, and labial bars; flanks with the same color or lighter than dorsum; groins, anterior and posterior surfaces of thighs, and concealed surfaces of tarsus and shanks yellow with black reticulations; ventral surfaces of thighs yellow; venter white with brown midline and reticulations; iris copper with or without a faint medial horizontal brown to reddish brown streak, with thin black reticulations (Fig. 26); (14) average SVL in adult females: 36.9 ± 2.2 mm (34.2–39.9 mm; *n* = 5); in adult males: 23.7 ± 2.9 mm (20.9–27.9 mm; *n* = 4).

#### Comparison with other species.

*Pristimantis phoxocephalus* is most similar to *P. atillo*, *P. jimenezi*, *P. teslai* sp. nov., *P. totoroi* sp. nov., and *P. verrucolatus* sp. nov. However, the coloration of the groins and concealed surfaces of thighs (yellow coloration with dark brown to black reticulations) distinguishes it from the species above (orange in *P. atillo*; different shades of brown with light brown to yellow flecks, spots or blotches in the other species). It can be further distinguished from *P. jimenezi* by having an advertisement call with shorter inter-note interval and a lower frequency of the second harmonic and final frequency of the note (Table 6). *Pristimantis teslai* sp. nov. has a tuberculate dorsum while in *P. phoxocephalus* is shagreen. *Pristimantis torresi* sp. nov. differs from *P. phoxocephalus* in having a golden to beige iris with a red to reddish-brown medial streak (copper with or without a faint red streak in *P. phoxocephalus*), and a wider head relative to its body (males *Z* = -2.56285, *p* = 0.0104, HW/SVL = 33.4–34.4% in *P. phoxocephalus*, 34.2–37.8% in *P. torresi* sp. nov.; females *Z* = -2.08893, *p* = 0.0367, HW/SVL = 33.1–37.3% in *P. phoxocephalus*, 36.7–38.5% in *P. torresi* sp. nov.). *Pristimantis totoroi* sp. nov. is the most similar and geographically closest species; both species differ by the coloration of the groins and iris (copper with or without a brown streak in *P. phoxocephalus*; golden with a red streak in *P. totoroi* sp. nov.) and by the presence of more distinctive tubercles and dermal folds in *P. totoroi* sp. nov. The advertisement call of *P. totoroi* sp. nov. has shorter inter-note intervals than *P. phoxocephalus* (Table 6). *Pristimantis verrucolatus* sp. nov. differs from *P. phoxocephalus* by having large tubercles and warts on flanks and by its advertisement call. Calls of *P. verrucolatus* sp. nov. have one note (3–9 in *P. phoxocephalus*) that lasts more than the notes of the calls of *P. phoxocephalus*, and the dominant frequency, second harmonic frequency, and final and initial frequency of the notes are lower (Table 6; Fig. 6).

#### Variation.

**Figure 27.**
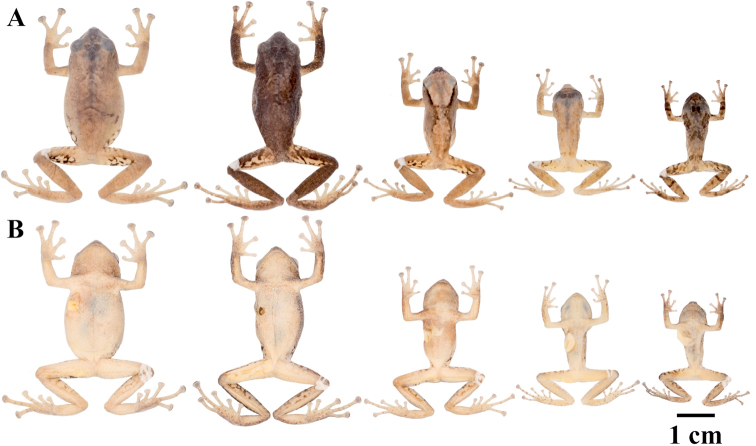
Color variation in preserved individuals of *Pristimantis phoxocephalus*. **A** Dorsal view of (from left to right): QCAZ 58463 (female), QCAZ 58472 (female), QCAZ 58470 (male), QCAZ 58468 (male), QCAZ 58461 (male). **B** Ventral view of the same specimens. See Suppl. material 2 for locality data. All specimens are shown at the same scale.

Variation in live and preserved individuals is shown in Figures 26, 27. This section is based on 23 individuals of the QCAZ collection collected at the type locality and its surroundings. Photographs are available from 12 individuals. Coloration in life is mentioned in parenthesis. Skin on dorsum is shagreen with scattered prominent tubercles; dorsum bears a faint middorsal fold; a middorsal row of two or more tubercles is present on the head; lateral folds are inconspicuous or absent. Folds and tubercles in juveniles are more prominent and larger than those in adults. In preservative, middorsal fold, lateral folds, and tubercles on dorsum can be lost. Dorsal coloration varies from cream, light gray and light brown to dark brown (cream, pale yellow or light to dark brown with or without reddish or olive tones). Dorsum can have a scapular W-shaped marking, chevrons, longitudinal middorsal band, pale blotches, and black or white flecking. Dorsal markings in juveniles are more evident than those in adults. Groins, anterior and posterior surfaces of thighs, and concealed surfaces of tarsus and shanks are uniformly white (yellow) in juveniles. Adults bear dark brown to black reticulations outlining well-defined pale (yellow) spots. Dorsal surfaces of thighs bear dark oblique bars, well defined on juveniles, ill-defined or absent on adults. Ventral surfaces of thighs are white or cream (yellow). Venter is white with a light brown midline (reddish cream) extending to the throat; throat is white (cream, sometimes yellow in males). Iris is copper with black reticulations in adults; sclera varies from cream to light blue. Juveniles have a faint medial horizontal red streak.

#### Advertisement call.

Based on recordings of two non-collected individuals at the type locality of *P. phoxocephalus* (November 17, 2014; 19h26; <10 °C). Advertisement calls of *P. phoxocephalus* consist of a series of 3–9 sharp notes (Fig. 6B). Notes last on average 0.154 s (range 0.133–0.175 s), silences between them, 0.135 s (range 0.110–0.159 s). The exact middle of the note presents the highest energy. The dominant and fundamental frequency of the notes is 2578 Hz. The note increases its frequency from beginning to end (2587–2452 Hz on average). Descriptive statistics for bioacoustic parameters are shown in Table 6.

#### Distribution, natural history, and conservation status.

*Pristimantis phoxocephalus* is known from its type locality, Pilaló, Cotopaxi Province, Ecuador, at 2340–2820 m a.s.l. (Fig. 2), which corresponds to Western Montane Forest. Individuals collected for this study were found by day inside bromeliads at 2–4 m above the ground. These bromeliads were on scattered trees within pastures. Lynch (1979) provides more ecological data for specimens collected at the type locality.

Currently, *P. phoxocephalus* is considered to be a Least Concern species (Rodríguez et al. 2004) under the assumptions: (i) Extent of Occurrence < 20000 km^2^, (ii) common and adaptable species with a presumed large population, (iii) unlikely to be declining fast enough to qualify for a more threatened category. After assessing the identity of this species, herein we assign it to the Critically Endangered Red List category following the B1ab(iii)+2ab(iii) IUCN criteria because: (i) it is known from one locality whose habitat is constantly degrading due to human settlements and cattle raising, (ii) Extent of Occurrence < 100 km^2^, (iii) Area of Occupancy < 10 km^2^.

#### Remarks.

Until now, *P. phoxocephalus* has been considered a single highly polymorphic species (e.g., Lynch 1979; Lynch and Duellman 1997; Duellman and Lehr 2009). We show that most populations previously ascribed to *P. phoxocephalus* represent other species (e.g., *Pristimantis atillo*, *P. jimenezi*, *P. teslai* sp. nov., *P. torresi* sp. nov., *P. totoroi* sp. nov., *P. verrucolatus* sp. nov., *Pristimantis* sp. (CCS2), *Pristimantis* sp. (UCS2), *Pristimantis* sp. (UCS3)). To determine the true identity of *P. phoxocephalus*, we made collections at the type locality (Pilaló, Cotopaxi Province, see Suppl. material 2 for the list of specimens). Surprisingly, sequences from Pilaló fell in two distinct clades, indicating the presence of two species. One juvenile (QCAZ 58425), collected at 2258 m, is actually *P. totoroi* sp. nov. All other specimens (e.g., QCAZ 58463) are *P. phoxocephalussensu stricto.* Examination of photographs of the holotype KU 142075 (Fig. 23A) showed unequivocally that it is conspecific with the specimens from the highland population. We included in our analysis sequences of “*P. phoxocephalus*” from two localities of Peru (Kañaris in Ferrenafe Province, Warmicocha-Cochapamba road in Chachapoyas Province); they do not belong to *Huicundomantis* (Suppl. material 3) and likely represent an undescribed species. Hence, *P. phoxocephalus* is endemic to Ecuador and has a restricted distribution.

### Pristimantis teslai sp. nov.

Taxon classificationAnimaliaAnuraStrabomantidae

5DB35A0E59D1556DA87A57D10898CF71

http://zoobank.org/1A73A8C1-771E-464A-86DA-F64E7FD30197

#### Common name.

English: Tesla’s Rain Frog. Spanish: Cutín de Tesla.

#### Holotype.

**Figure 28.**
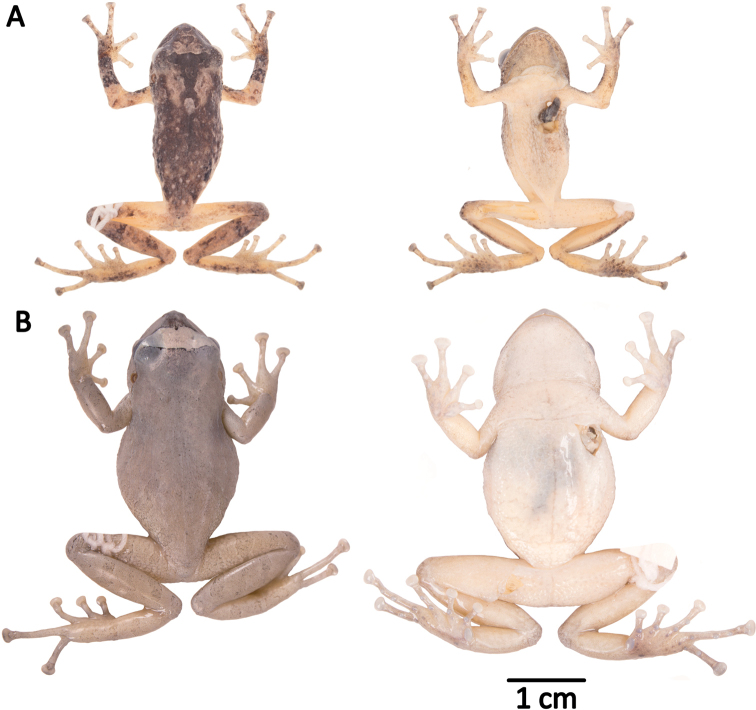
Holotypes of *Pristimantis teslai* sp. nov. and *P. torresi* sp. nov. Photographs of preserved holotypes of **A***P. teslai* (QCAZ 46213, male) and **B***P. torresi* (QCAZ 47342, female). Dorsal view on the left, ventral view on the right. Specimens are shown at the same scale.

QCAZ 46213, an adult male from Llanganatillo, Llanganates National Park border, Tungurahua Province, Ecuador (1.2658S, 78.4459W, 3600 m), collected by Rodrigo Toscano and Silvia Aldás-Alarcón on November 11, 2009. Figure 28A.

#### Paratopotypes

**(4: 3 males, 1 juvenile).**QCAZ 46108, QCAZ 46208, QCAZ 46211, adult males, QCAZ 46209, juvenile, collected by Elicio E. Tapia, Silvia Aldás-Alarcón, and Rodrigo Toscano in November 2009.

#### Diagnosis.

**Figure 29.**
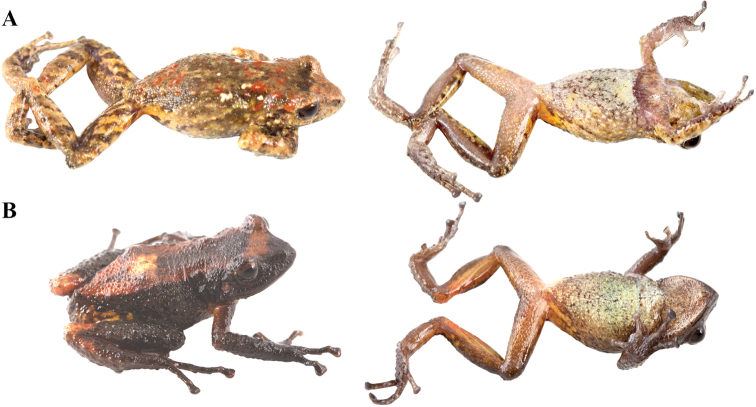
Color variation in live individuals of *Pristimantis teslai* sp. nov. **A**QCAZ 46208 (male, SVL 23.4 mm) **B**QCAZ 46209 (juvenile female, SVL 29.4 mm). Dorsolateral view on the left, ventral view on the right.

A species of the *Pristimantis phoxocephalus* group having the following combination of characters: (1) dorsal surfaces tuberculate, tubercles on anterior dorsum prominent, small and rounded, those on posterior dorsum larger; middorsal, dorsolateral, and lateral folds absent; head with two small middorsal tubercles; skin on flanks as or more tuberculate than dorsum, bearing scattered warts; skin on venter coarsely areolate; discoidal fold present; (2) tympanic membrane and tympanic annulus prominent, its upper and posterior margin concealed by thick supratympanic fold; (3) snout moderately long, acuminate with a fleshy keel in dorsal view, protruding in profile; (4) upper eyelid with distinct rounded tubercles surrounded by smaller tubercles; cranial crests absent; (5) dentigerous processes of vomers low to prominent, oblique, moderately separated, posteromedial to choanae; (6) vocals slits, vocal sac, and nuptial pads present in adult males; (7) Finger I shorter than Finger II; discs of digits expanded to broadly expanded, elliptical to truncate; (8) fingers with broad lateral fringes; (9) distinct, rounded ulnar tubercles; (10) heel bearing a prominent round tubercle surrounded by smaller tubercles; inner and outer edge of tarsus bearing a row of prominent, rounded tubercles; (11) inner metatarsal tubercle elliptical, elevated four times the size of round, elevated outer metatarsal tubercle; supernumerary tubercles distinct, as large as outer metatarsal tubercle; (12) toes with lateral fringes; basal webbing present; Toe V longer or much longer than Toe III (disc on Toe III reaches distal edge of penultimate subarticular tubercle on Toe IV, disc on Toe V reaches the proximal to distal edge of distal subarticular tubercle on Toe IV); toe discs smaller than those on fingers, elliptical to truncate (Fig. 9A); (13) in life, dorsum olive to reddish brown with irregular red or cream markings; groins and anterior surfaces of thighs dark brown with yellow blotches irregularly bordered; dorsal surfaces of thighs with irregular oblique yellow stripes, posterior surfaces of thighs brown with yellow flecks; venter dusty white with scattered brown flecks, with or without faint yellow blotches; iris copper with thin black reticulations (Fig. 29); (14) average SVL in adult males: 25.2 ± 1.8 mm (23.4–27.3 mm; *n* = 4); females: unknown.

#### Comparison with other species.

*Pristimantis teslai* is most similar to *P. atillo*, *P. jimenezi*, *P. percultus*, *P. phoxocephalus*, *P. torresi* sp. nov., *P. totoroi* sp. nov., and *P. verrucolatus* sp. nov. They share an acuminate and protruding snout with a keel at the tip. However, (except for *P. percultus*) *P. teslai* is unique among them by having the dorsum covered by prominent rounded tubercles. Additionally, lack of lateral folds distinguishes *P. teslai* from *P. jimenezi*, *P. totoroi* sp. nov., *P. torresi* sp. nov., and *P. verrucolatus* sp. nov. Groins of *P. teslai* are dark brown with yellow blotches irregularly bordered, different from those of *P. atillo* (groins orange surrounded or not by yellow blotches), *P. jimenezi* (pinkish, purplish or dark brown with small light brown to yellow spots), *P. phoxocephalus* (yellow with dark brown to black reticulations), and *P. verrucolatus* sp. nov. (reddish brown with light brown to yellow spots). In males, the tympanum diameter is significantly larger than that of *P. totoroi* sp. nov. (males *Z* = -2.63144, *p* = 0.0085, TD/SVL = 5.0–5.5% in *P. teslai*, 4.4–5.1% in *P. totoroi* sp. nov). *Pristimantis teslai* is smaller than *P. verrucolatus* sp. nov. (males *Z* = 2.35, *p* = 0.0188, SVL = 23.4–27.3 mm in *P. teslai*, 25.1–34.5 mm in *P. verrucolatus* sp. nov). It can be distinguished from *P. percultus* by the absence of cranial crests (low in *P. percultus*), the coloration of the iris (copper with thin reticulations in *P. teslai*; golden with wide black reticulations in *P. percultus*), and lacking the red labial stripe, characteristic of *P. percultus*.

#### Description of the holotype.

Adult male (QCAZ 46213, SC29676). Measurements (in mm): SVL 27.3; TL 12.8; FL 12.9; HL 9.2; HW 9.5; ED 2.9; TD 1.4; IOD 3.1; EW 2.7; IND 2.3; EN 2.7; TED 1.0. Head wider than long, as wide as body; snout moderately long, acuminate in dorsal view, protruding in profile, bearing a fleshy keel; cranial crests absent; nostrils slightly protuberant, ovoid, directed laterally with slight dorsal inclination; canthus rostralis slightly concave in dorsal view, rounded in cross section; loreal region slightly concave; upper eyelid with prominent, rounded, medium sized tubercles surrounded by smaller tubercles; tympanic membrane distinct; tympanic annulus prominent, upper and posterior edge concealed by supratympanic fold; two prominent rounded postrictal tubercles. Choanae large, ovoid, not concealed by palatal shelf of maxillae; dentigerous processes of vomers small, prominent, oblique, narrowly separated, positioned posteromedial to choanae; each vomer bearing several teeth; tongue longer than wide, slightly notched, posterior three fifths not adherent to floor of mouth; vocal slits slightly curved, located at posterior half of mouth floor in between tongue and margin of jaw; vocal sac present.

Dorsal surfaces of body tuberculate, head and anterior dorsum with small prominent rounded tubercles, posterior dorsum with medium sized tubercles; middorsal, dorsolateral and lateral folds absent; head bears two prominent middorsal tubercles; flanks with the same texture as dorsum, bearing scattered warts; skin on venter coarsely areolate, ventral surfaces of limbs smooth, ventral surfaces of thighs coarsely areolate; discoidal fold absent. Low and round ulnar tubercles; white nuptial pads; outer palmar tubercle bifid, almost twice the size of ovoid thenar tubercle; subarticular tubercles prominent, rounded; low supernumerary tubercles at the base of fingers; fingers bearing broad lateral fringes; Finger I shorter than Finger II; discs on fingers expanded, truncate; pads on fingers surrounded by circumferential grooves on all fingers (Fig. 9A).

Hindlimbs slender; dorsal surfaces of hindlimbs tuberculate; posterior surfaces of thighs smooth, ventral surfaces of thighs coarsely areolate; heel bearing a medium sized, prominent and rounded tubercle surrounded by several slightly smaller tubercles; outer and inner edge of tarsus bearing distinct, rounded tubercles; inner metatarsal tubercle elliptical, elevated 4 × the size of round, elevated outer metatarsal tubercle; supernumerary tubercles as large as outer metatarsal tubercle; subarticular tubercles prominent, rounded; toes bearing lateral fringes; basal webbing between toes IV and V present; discs on toes smaller than those on fingers, expanded and elliptical; toes having pads surrounded by circumferential grooves; relative lengths of toes: I < II < III < V < IV; Toe V much longer than Toe III (disc on Toe III reaches distal edge of penultimate subarticular tubercle on Toe IV, disc on Toe V reaches distal edge of distal subarticular tubercle on Toe IV; Fig. 9A). Coloration of the holotype in preservative is shown in Figure 28A.

*Coloration of holotype in preservative*. Dorsum brown with a light brown W-shaped scapular mark and interscapular blotch; head with dark brown supratympanic stripe, canthal and interorbital bands; dorsal surfaces of forelimbs, shanks and tarsus light brown with dark brown transversal bands; armpits, groins, anterior, and posterior surfaces of thighs cream; posterior surfaces of thighs brown with yellow flecks; venter cream with scattered brown flecks; throat, soles, and palms dusty cream (Fig. 28A).

*Coloration of holotype in life*. Unknown.

#### Variation.

**Figure 30.**
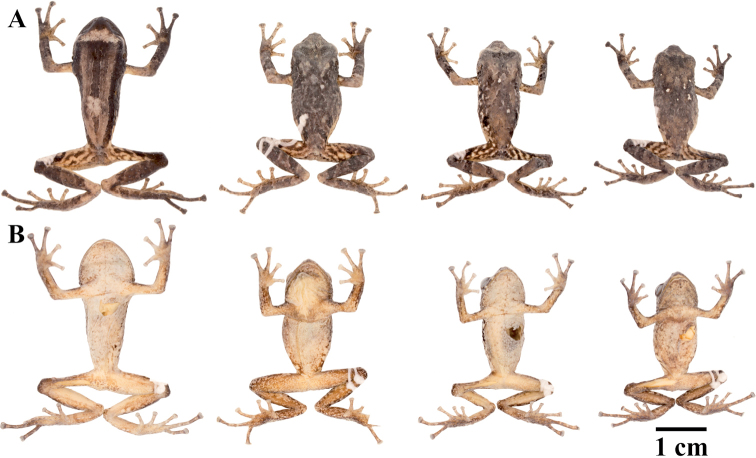
Color variation in preserved individuals of *Pristimantis teslai* sp. nov. **A** Dorsal view of (from left to right): QCAZ 46209 (juvenile female), QCAZ 46108 (male), QCAZ 46211 (male), QCAZ 46208 (male) **B** Ventral view of specimens in A. See Suppl. material 2 for locality data. All specimens are shown at the same scale.

Variation in preservative is shown in Figure 30, where all the type series is included. Coloration in life is known for two individuals; their photographs are shown in Figure 29. Coloration in life is in parenthesis. Background coloration of dorsal surfaces vary from gray to brown (olive to reddish brown); dorsum might bear dark brown W-shaped scapular marking, irregular chevrons, or longitudinal stripes (dorsum with red or cream irregular spots or blotches); head bears or not dark brown supratympanic stripe, canthal stripe, interorbital band and labial bars; flanks with or without black or white spots. Groins and anterior surfaces of thighs dark brown with pale (yellow) blotches irregularly bordered; posterior surfaces of thighs brown with pale (yellow) flecks; dorsal surfaces of thighs with irregular oblique pale (yellow) stripes, venter white to dusty white with scattered brown flecks (dusty white with or without faint yellow blotches). Iris is copper with thin black reticulations; light-blue sclera. Tubercles of the individual with the striped color pattern on dorsum are less prominent than those of the other individuals.

#### Distribution, natural history, and conservation status.

*Pristimantis teslai* is known from two Paramo localities in the eastern Andean slopes in Tungurahua Province (Fig. 2). Individuals were found beneath rocks, among moss or bunch grasses at day, or active on low vegetation up to 80 cm above ground at night. Parque Nacional Llanganates is, to a large extent, unexplored. It could have additional populations for this species. Given the scant available information, we assign *P. teslai* to the Data Deficient Red List Category (IUCN 2017).

#### Etymology.

The specific epithet is a noun in the genitive case and is a patronym for Nikola Tesla, a revolutionary inventor of the late 19^th^ and early 20^th^ century. It is named after him in recognition of his contributions to physics and his dedication to the ideal of providing free wireless electric power.

#### Remarks.

*Pristimantis teslai* has been mistakenly identified as *P. phoxocephalus* (e.g., collections at the QCAZ museum). Here, we recognize it as a different species and assign it to the *P. phoxocephalus* species group. *Pristimantis teslai* is most similar species to UCS1. They are sister species and have a genetic distance of 2.5%. *Pristimantis teslai* differs from UCS1 by having more prominent tubercles and smaller and fuzzier yellow blotches on the groins and posterior surfaces of thighs. However, these small differences may represent intraspecific variation. Hence, the status of UCS1 will remain tentative until additional specimens and populations of both species are examined.

### Pristimantis torresi sp. nov.

Taxon classificationAnimaliaAnuraStrabomantidae

4FFD270148D35548960B3C83B72FEC13

http://zoobank.org/1E92D590-2072-4530-88C6-2019A0A6D8F9

#### Common name.

English: Torres’ Rain Frog. Spanish: Cutín de Torres.

#### Holotype.

QCAZ 47342, adult female from Guanchanamá, military antennas, Loja Province, Ecuador (4.0511S, 79.8693W, 2729 m), collected by Elicio E. Tapia and Margarita Baquero on February 21, 2010. Figure 28B.

#### Paratypes

**(46: 16 males, 6 females, 24 juveniles).** Ecuador: Loja Province: QCAZ 47343, QCAZ 47354, adult females, QCAZ 47344, adult male, QCAZ 47340–341, QCAZ 47345–351, juveniles, collected with the holotype; QCAZ 47397, QCAZ 47405, QCAZ 47409, QCAZ 47414, QCAZ 47416–418, QCAZ 47503, adult males, from Celica-Alamor road (4.0990S, 79.9799W, 2101 m), collected by Elicio E. Tapia and Freddy Velásquez on February 27, 2010; QCAZ 64524, adult male, QCAZ 64525, QCAZ 64527–529, QCAZ 64555, juveniles, from Guanchanamá, military antennas (4.0511S, 79.8693W, 2729 m), collected by Nadia Páez, Fernando Ayala, Pablo Sandoval and Ricardo Gavilanes in August 2016; QCAZ 64533, QCAZ 64538, juveniles, from Celica-Guachanamá road (4.0907S, 79.9509W, 2348 m), collected by Nadia Páez, Fernando Ayala, Pablo Sandoval and Ricardo Gavilanes in August 2016; QCAZ 64544, juvenile from Guachanamá-La Tolera road (4.0321S, 79.8836W, 2736 m), collected by Nadia Páez, Fernando Ayala, Pablo Sandoval and Ricardo Gavilanes in August 2016; QCAZ 64549, adult male, QCAZ 64551, QCAZ 64556, juveniles, from Celica–Alamor road (4.0974S, 79.9796W, 2117 m), collected by Nadia Páez, Fernando Ayala, Pablo Sandoval and Ricardo Gavilanes in August 2016. Nearby Guachanamá, collected by Diego Almeida, Darwin Núñez, Eloy Nusirquia, Santiago Guamán and Guadalupe Calle in November 2016: QCAZ 65762, adult male, (4.0441S, 79.8814W, 2664 m); QCAZ 65766–767, adult females (4.0410S, 79.8827W, 2770); QCAZ 65769, adult male (4.0408S, 79.8829W, 2787 m); QCAZ 65771, adult male (4.0431S, 79.8817W, 2738 m); QCAZ 65774, QCAZ 65776, adult males (4.0441S, 79.8814W, 2646 m). Nearby La Tolera, collected by Diego Almeida, Darwin Núñez, Eloy Nusirquia, Santiago Guamán and Guadalupe Calle in November 2016: QCAZ 65779, adult female (4.0317S, 79.8824W, 2737 m); QCAZ 65782, adult female, QCAZ 65780, juvenile (4.0326S, 79.8836W, 2752 m); QCAZ 65783–786, juveniles (4.0395S, 79.8848W, 2833 m).

#### Diagnosis.

**Figure 31.**
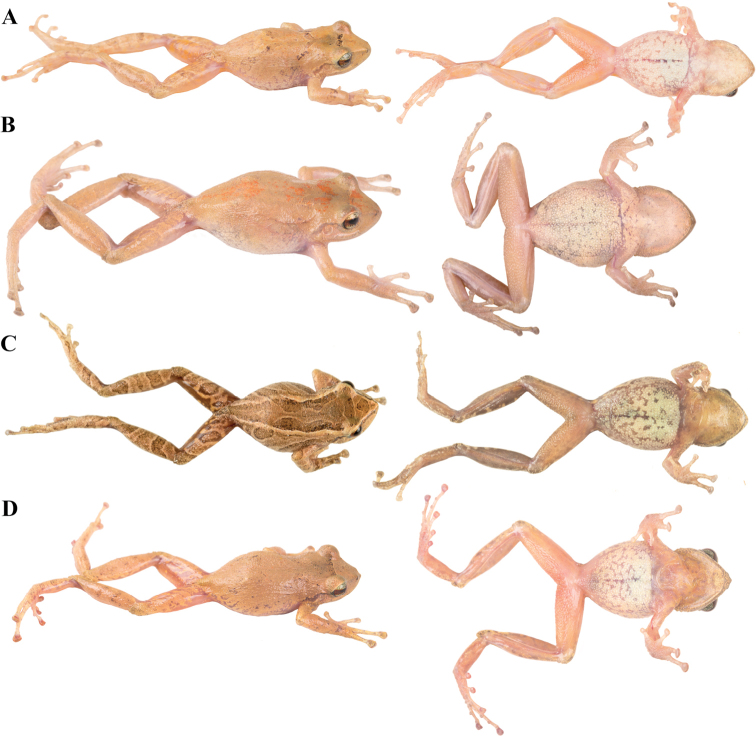
Color variation in live individuals of *Pristimantis torresi* sp. nov. **A**QCAZ 64524 (male, SVL 26.7 mm) **B**QCAZ 65767 (female, SVL 35.8 mm) **C**QCAZ 65771 (male, SVL 28.7 mm) **D**QCAZ 65779 (male, SVL 26.4 mm). Dorsolateral view on the left, ventral view on the right.

A member of the *Pristimantis phoxocephalus* group characterized by the following combination of characters: (1) skin on dorsum shagreen; thin middorsal fold; head with a middorsal small tubercle or row of tubercles; dorsolateral folds absent; flanks with longitudinal lateral folds on anterior half; skin on venter areolate to weakly areolate; discoidal fold present or absent; (2) tympanic membrane and tympanic annulus prominent, its upper and posterior margin concealed by supratympanic fold; (3) snout moderately long, acuminate with a fleshy keel in dorsal view, protruding in profile; (4) upper eyelid with a small and rounded tubercle, surrounded by several lower tubercles; cranial crests absent; (5) dentigerous processes of vomers prominent, oblique, moderately to broadly separated, posteromedial to choanae; (6) vocals slits, vocal sac and nuptial pads present; (7) Finger I shorter than Finger II; discs of digits broadly expanded, elliptical to truncate; (8) fingers with lateral fringes; (9) ulnar tubercles ill-defined; (10) heel bearing a small, rounded tubercle surrounded or not by smaller tubercles; outer and inner tarsal tubercles absent or ill-defined; inner tarsal fold present; (11) inner metatarsal tubercle ovoid, rounded, elevated, six times the size of round outer metatarsal tubercle; supernumerary tubercles numerous; (12) toes with broad lateral fringes; basal webbing present; Toe V longer or much longer than Toe III (disc on Toe III reaches the middle or exceeds distal edge of penultimate subarticular tubercle on Toe IV; disc on Toe V reaches the middle or exceeds distal edge of distal tubercle on Toe IV); toe discs as large or slightly smaller than those on fingers (Fig. 9B); (13) in life, dorsum cream, brown or orangey brown; head bearing a dark supratympanic stripe and interorbital band or stripe; coloration of groins and concealed surfaces of thighs have sexual dimorphism: in females (and juveniles), ground color varies from light purplish brown to medium brown with or without minute cream flecks on; males have the same ground color with yellow, cream or orangey-yellow small to big spots; venter white to dusty cream; iris golden to beige with black reticulations and a red or reddish brown medial streak (Fig. 31); (14) average SVL in adult females: 34.7 ± 3.7 mm (30.1–39.5 mm; *n* = 5); in adult males: 25.9 ± 2.1 mm (23.3–30.0 mm; *n* = 18).

#### Comparison with other species.

*Pristimantis torresi* is similar to *P. atillo*, *P. jimenezi*, *P. phoxocephalus*, *P. teslai*, *P. totoroi* sp. nov., and *P. verrucolatus* sp. nov., which also have an acuminate snout with a fleshy keel. *Pristimantis atillo* usually has orange groins and black dots on the flanks, instead, *P. torresi* has brown groins with or without yellow spots, and its flanks lack black dots. The most similar species to *P. torresi* is *P. jimenezi*, whose iris varies from copper to red (golden to beige with a red medial streak in *P. torresi*). *Pristimantis torresi* differs from *P. phoxocephalus* in having a golden to beige iris (copper in *P. phoxocephalus*), brown groins with or without light brown to yellow spots (yellow with black reticulations in *P. phoxocephalus*), and a wider head relative to its body (males *Z* = -2.56285, *p* = 0.0104, HW/SVL = 33.4–34.4% in *P. phoxocephalus*, 34.2–37.8% in *P. torresi*; females *Z* = -2.08893, *p* = 0.0367, HW/SVL = 33.1–37.3% in *P. phoxocephalus*, 36.7–38.5% in *P. torresi*). *Pristimantis torresi* is easy to distinguish from *P. teslai* by having shagreen dorsal skin, lateral folds, and golden to beige iris with a red to reddish-brown medial streak (tuberculate dorsal skin, lateral folds absent and copper iris in *P. teslai*). *Pristimantis totoroi* sp. nov. has more prominent tubercles and folds than those of *P. torresi*, and its head is longer (males *Z* = 3.84623, *p* = 0.0001, HL/HW = 95.1–102.3% in *P. torresi*, 99.7–104% in *P. totoroi* sp. nov; females *Z* = -2.76079, *p* = 0.0058, HL/HW = 90.6–94.7% in *P. torresi*, 95.6–103.2% in *P. totoroi* sp. nov), relative to head width. *Pristimantis torresi* differs from *Pristimantis verrucolatus* sp. nov. in lacking large tubercles and warts on the flanks (present in *P. verrucolatus*) and having a golden to beige iris (coppery brown in *P. verrucolatus*).

#### Description of the holotype.

Adult female (QCAZ 47342, SC32171). Measurements (in mm): SVL 35.8; TL 17.5; FL 16.4; HL 12.4; HW 13.2; ED 3.7; TD 1.8; IOD 3.8; EW 3.1; IND 2.8; EN 3.5; TED 0.8. Head wider than long, narrower than body; snout moderately long, acuminate with a fleshy keel in dorsal view, protruding in profile; nostrils slightly protuberant, directed anterolaterally; canthus rostralis distinct, curved in dorsal view, rounded in cross-section; loreal region concave; upper eyelid bearing a small rounded tubercle surrounded by several lower tubercles; tympanic membrane and tympanic annulus distinct, its upper and posterior margin covered by supratympanic fold; one enlarged subconical postrictal tubercle surrounded by several smaller tubercles. Choanae median, semicircular, not concealed by palatal shelf of maxilla; dentigerous processes of vomers prominent, oblique, moderately separated, posteromedial to choanae; each vomer bearing several indistinct teeth; tongue as wide as long, posteriorly notched, free on posterior half of its length.

Skin on dorsum shagreen; faint middorsal fold; head with a middorsal row of two small tubercles; dorsolateral folds absent; flanks areolate, with thin lateral folds on anterior half; skin on belly and chest areolate, skin on throat, chest and ventral surfaces of limbs smooth; discoidal fold present. Ulnar tubercles present, indistinct; palmar tubercles prominent, outer palmar tubercle bifid, almost twice size of ovoid thenar tubercle; subarticular tubercles prominent, rounded; supernumerary tubercles at base of fingers distinct; fingers with broad lateral fringes; Finger I shorter than Finger II; discs broadly expanded and rounded; pads on fingers surrounded by circumferential grooves on all fingers (Fig. 9B).

Hindlimbs slender; dorsal surfaces of hindlimbs smooth; posterior surfaces of thighs smooth, ventral surfaces of thighs areolate; heel bearing a low rounded tubercle surrounded by some smaller tubercles; outer tarsal tubercles absent; inner tarsal fold extend to half of length of tarsus; inner metatarsal tubercle ovoid, rounded, elevated, six times the size of rounded, ill-defined outer metatarsal tubercle; plantar surface with supernumerary tubercles, those on the base of toes low but distinct; subarticular tubercles prominent, rounded; toes with broad lateral fringes; basal webbing present; discs nearly as large as those on fingers, elliptical; all toes having pads surrounded by circumferential grooves; relative lengths of toes: I < II < III < V < IV; Toe V much longer than Toe III (disc on Toe III reaches the middle of the penultimate subarticular tubercle on Toe IV, disc on Toe V exceeds distal edge of distal subarticular tubercle on Toe IV; Fig. 9B). Coloration of the holotype in preservative is shown in Figure 28B; coloration in life is unknown.

*Coloration of holotype in preservative*. Dorsal surface of body light gray; head with cream interorbital band outlined with black, dark brown supratympanic stripes, and faint gray labial bars; limbs with transversal bands slightly darker than background; dorsolateral surfaces with oblique reticulations extending to the flanks, slightly darker than background; groins and concealed surfaces of thighs gray with small cream spots; ventral surfaces of body white, venter with faint gray flecks and midventral longitudinal line; plants and palms dusty cream (Fig. 28B).

*Coloration of holotype in life*. Unknown.

#### Variation.

**Figure 32.**
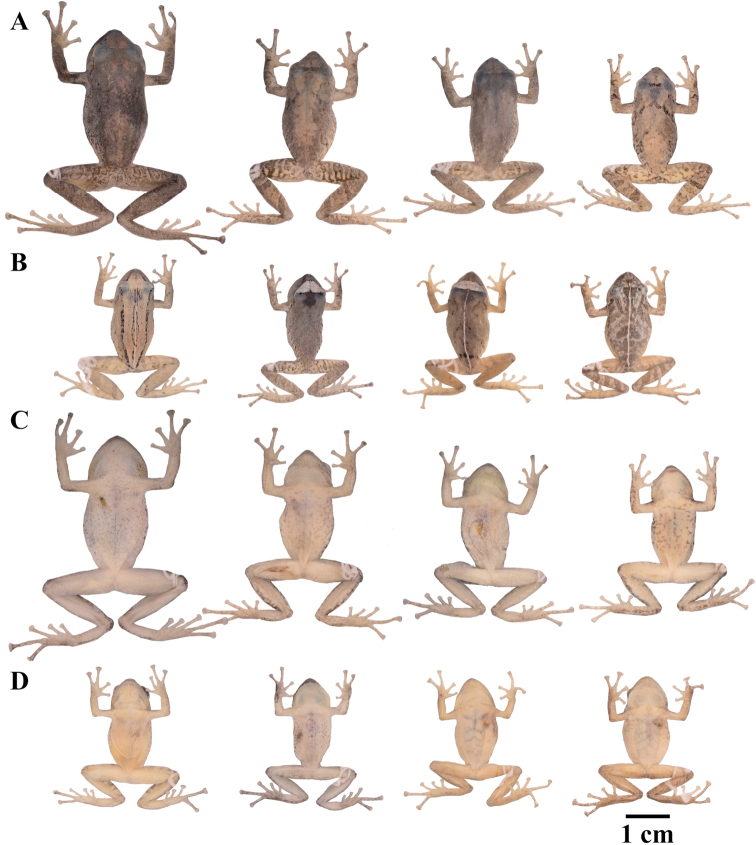
Color variation in preserved individuals of *Pristimantis torresi* sp. nov. **A** Dorsal view of (from left to right): QCAZ 65767 (female), QCAZ 65762 (male), QCAZ 65769 (male), QCAZ 64524 (male) **B** Dorsal view of: QCAZ 64549 (male), QCAZ 65782 (male), QCAZ 47397 (male), QCAZ 47416 (male) **C** Ventral view of specimens in (**A**) **D** Ventral view of specimens in B. See Suppl. material 2 for locality data. All specimens are shown at the same scale.

Based on the 47 preserved specimens of the type series and photographs for 24 individuals. Variation in living and preserved individuals is shown in Figures 31, 32. Coloration in life is given in parenthesis. Dorsum coloration varies between cream, light gray and brown (cream, brown or orangey-brown). Some individuals present dark brown reticulations, chevrons, flecks, a middorsal band, an hourglass-shaped band or a thin white middorsal stripe on dorsum. Head always bears brown supratympanic stripes and interorbital stripes or bands; sometimes, it bears brown canthal stripes or labial bars. Limbs present faint or well-defined transversal bands. Flanks may have diagonal bars. Coloration of groins and concealed surfaces of hindlimbs has sexual dimorphism: both sexes have cream, gray or brown backgrounds (light purplish brown to medium brown); females have none or little pale (cream) flecks; males have small or big pale (yellow, cream or orangey yellow) spots, usually more conspicuous in larger males. Venter varies from white to dusty cream, usually with gray or light brown markings and a midline (in males, throat cream, light or bright yellow). Iris is golden to beige with black reticulations and a red or reddish-brown medial streak; sclera varies from cream to light blue. Coloration in life of each individual may change; the ground color of the hidden surfaces of thighs and other markings as the supratympanic stripe can vary from light purplish brown to medium brown in the same individual.

#### Distribution, natural history, and conservation status.

This species is known from the surroundings of Celica, Guachanamá and La Tolera, towns in the western Andean slopes of Loja Province in Ecuador (Fig. 2). It inhabits Inter-Andean Shrub region between 2101 and 2833 m a.s.l. Most individuals were found at night inside terrestrial or arboreal bromeliads up to 3 m above the ground, inside patches of native vegetation, or on low vegetation up to 60 cm above ground. During the day they were only found inside bromeliads. Calling males have been found on low vegetation, 30 cm above the ground in February.

We propose assigning *P. torresi* to the Critically Endangered Red List category following the B1ab(iii) IUCN criteria. Available records come from two localities (sensu IUCN 2017) whose habitat is being degraded by human settlements, cattle raising and agriculture; the Extent of Occurrence of the species < 100 km^2^ (21 km^2^).

#### Etymology.

The specific epithet is a noun in the genitive case and is a patronym for Omar Torres-Carvajal, curator of reptiles of Museo de Zoología at Pontificia Universidad Católica del Ecuador. The species name is in recognition of his significant contributions to herpetological research in Ecuador and the development of collections at the QCAZ museum.

### Pristimantis totoroi sp. nov.

Taxon classificationAnimaliaAnuraStrabomantidae

F22567F5929355799C460E404CB4608F

http://zoobank.org/C1E75C61-45D3-41E3-84E8-99721B713E76

#### Common name.

English: Totoras Rain Frog. Spanish: Cutín de Totoras.

#### Holotype.

**Figure 33.**
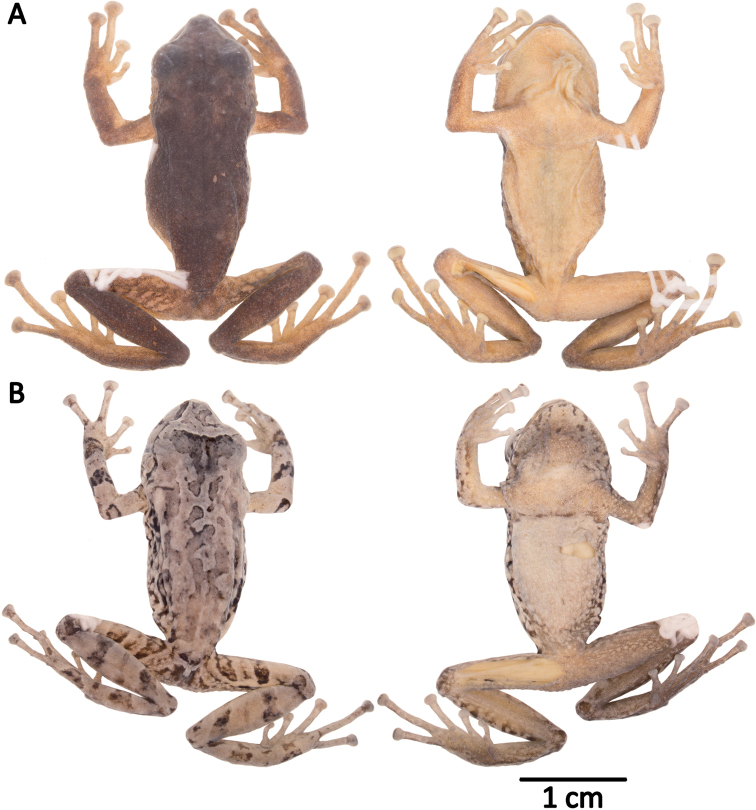
Holotypes of *Pristimantis totoroi* sp. nov. and *P. verrucolatus* sp. nov. Photographs of preserved holotypes of **A***P. totoroi* (QCAZ 25105, male) and **B***P. verrucolatus* (QCAZ 46982, male). Dorsal view on the left, ventral view on the right. Specimens are shown at the same scale.

QCAZ 25105, an adult male from Cashca Totoras Protected Forest, Bolívar Province, Ecuador (1.7188S, 78.9749W, 3200 m), collected by Rachel Kosoff and Ítalo G. Tapia on December 14, 2001. Figure 33A.

#### Paratypes

**(41: 22 males, 5 females, 14 juveniles).** Ecuador: Bolívar Province: Cashca Totoras Protected Forest: QCAZ 1021, QCAZ 1024, adult females, QCAZ 1020, QCAZ 1022–1023, juveniles, collected by Giovanni Onore in December 1987; QCAZ 14704–705, adult males, collected by Chris Funk in December 2000; QCAZ 16834–836, adult males, collected by Santiago Ron in January 2001; QCAZ 16916–922, adult males (1.7115S, 78.9781W, 3000 m), collected by Luis Coloma and Martín Bustamante in October 2000; QCAZ 12580, adult female (1.7200S, 78.9590W, 3200 m), collected by Felipe Campos and Stella de la Torre in December 1987; QCAZ 16718, QCAZ 16720–721, QCAZ 16731, adult males, QCAZ 16719, QCAZ 16725, QCAZ 16728, juveniles (1.7235S, 78.9802W, 2923 m), collected by Santiago Ron, Martín Bustamante, Ítalo Tapia and Mónica Guerra in August 2001; QCAZ 25122, QCAZ 25128, QCAZ 25135, adult males, QCAZ 25124, QCAZ 25127, QCAZ 25134, juveniles (1.7167S, 78.9667W, 2960 m), collected by Rachel Kosoff and Ítalo Tapia in December 2001; QCAZ 31505, adult female, QCAZ 31502, adult male, QCAZ 31501, QCAZ 31503–504, juveniles (1.7255S, 78.9806W, 2867 m), collected by Martín Bustamante and Galo Díaz in July 2002; QCAZ 32039, adult female, QCAZ 32040, adult male (1.7255S, 78.9806W, 2847 m), collected by Martín Bustamante in November 2002. Cotopaxi Province: QCAZ 58425, juvenile, from Pilaló surroundings (0.9713S, 78.9987W, 2258 m), collected by Santiago Ron, Pablo Venegas, Nadia Páez and Pamela Baldeón in November 2014. Bolívar Province: QCAZ 49511, adult male, QCAZ 49509, juvenile, from San Vicente (1.7120S, 79.0170W, 2408 m), collected by Luis Coloma and Ernesto Martínez in December 1984.

**Referred specimens (1)**: KU 218025 from 70 km W Riobamba, Riobamba-Pallatanga road (1.9460S, 78.9468W, 2430 m), Chimborazo Province, Ecuador.

#### Diagnosis.

**Figure 34.**
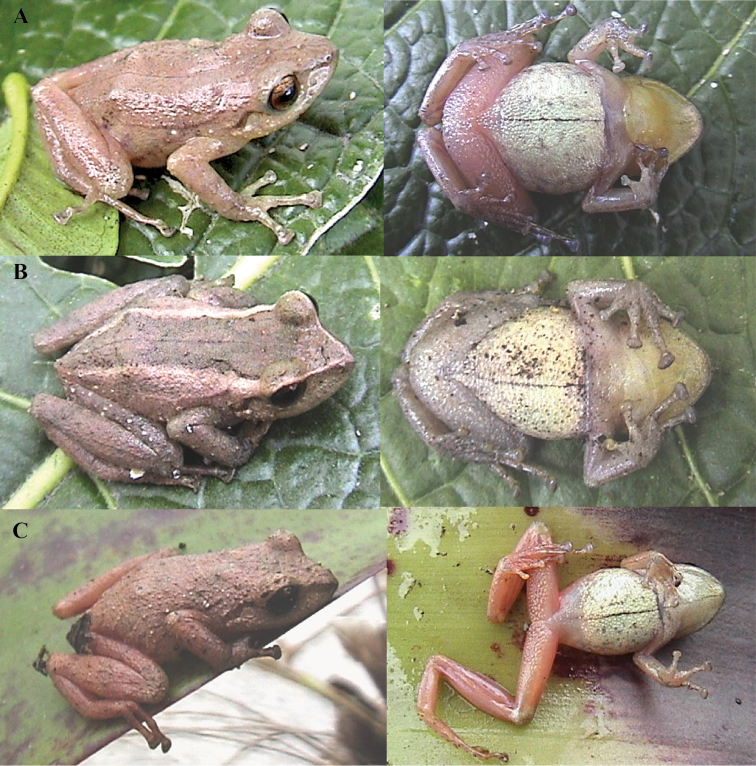
Color variation in live individuals of *Pristimantis totoroi* sp. nov. **A**QCAZ 16718 (male, SVL 24.7 mm) **B**QCAZ 16721 (male, SVL 24.1 mm) **C**QCAZ 16725 (juvenile female, SVL 23.7 mm). Dorsolateral view on the left, ventral view on the right.

A species of the *Pristimantis phoxocephalus* group having the following combination of characters: (1) skin on dorsum shagreen with or without scattered small tubercles; middorsal fold; head with a middorsal row of two or more tubercles; dorsolateral folds absent; lateral folds on anterior half of dorsum; skin on venter coarsely areolate; discoidal fold evident; (2) tympanic membrane and tympanic annulus prominent, its upper and posterior margin covered by supratympanic fold; (3) snout moderately long, acuminate with a fleshy keel in dorsal view, protruding in profile; (4) upper eyelid with small tubercles; cranial crests absent; (5) dentigerous processes of vomers low to prominent, oblique, moderately separated, posteromedial to choanae; (6) vocals slits, vocal sac, and prominent nuptial pads present in adult males; (7) Finger I shorter than Finger II; discs of digits broadly expanded, rounded to elliptical; (8) fingers with broad lateral fringes; (9) small distinct ulnar tubercles; (10) heel bearing a subconical tubercle surrounded by smaller tubercles; outer edge of tarsus bearing subconical tubercles; inner edge of tarsus with a fold, followed or not by a row of small tubercles; (11) inner metatarsal tubercle ovoid, elevated, 5 times the size of round outer metatarsal tubercle; supernumerary tubercles numerous, elevated; (12) toes with broad lateral fringes; basal webbing present; Toe V much longer than Toe III (disc on Toe III reaches the middle of penultimate subarticular tubercle on Toe IV or slightly exceeds its distal edge, disc on Toe V reaches the middle of distal subarticular tubercle on Toe IV or slightly exceeds its distal edge); toe discs smaller than those on fingers, rounded to elliptical (Fig. 9C); (13) in life, dorsal coloration varies from cream to dark brown; flanks with dark diagonal bars; groins and posterior surfaces of thighs cream to brown with or without pale spots; venter white, bearing a midline extended or not to the throat; ventral surfaces of thighs light to dark gray, orange or cream; iris golden with a medial horizontal red streak and fine black reticulations (Fig. 34); (14) average SVL in adult females: 33.0 ± 0.9 mm (31.9–34.3 mm; *n* = 7); in adult males: 26.8 ± 2.0 mm (23.2–29.4 mm; *n* = 21).

#### Comparison with other species.

*Pristimantis totoroi* is similar to *P. atillo*, *P. jimenezi*, *P. phoxocephalus*, *P. teslai*, *P. torresi* and *P. verrucolatus* sp. nov., which also have an acuminate snout with a fleshy keel. The texture of its skin is different from the other species. *Pristimantis totoroi* has a shagreen dorsal skin, which is tuberculate in *P. atillo*; it lacks the large tubercles and warts on flanks present in *P. verrucolatus* sp. nov.; its tubercles and lateral folds are more prominent than those of *P. atillo*, *P. jimenezi*, *P. phoxocephalus*, and *P. torresi*. The coloration of the iris, golden with a red medial streak, helps distinguish *P. totoroi* from *P. atillo* (copper), *P. jimenezi* (copper to red), *P. phoxocephalus* (copper), *P. teslai* (copper), and *P. verrucolatus* sp. nov. (coppery brown). Additionally, the advertisement call of *P. totoroi* is different from the available calls of other species of the group. Notes of call of *P. totoroi* are shorter than those of *P. jimenezi* and *P. verrucolatus* sp. nov., inter-note intervals are longer than those of *P. phoxocephalus*, dominant frequency and frequency of the second harmonic are lower than those of *P. jimenezi* and higher than *P. verrucolatus* sp. nov. (Table 6).

#### Description of the holotype.

An adult male (QCAZ 25105, SC7093). Measurements (in mm): SVL 29.4; TL 13.9; FL 13.8; HL 10.2; HW 9.8; ED 3.6; TD 1.3; IOD 3.0; EW 3.0; IND 2.2; EN 2.9; TED 1.3. Head longer than wide, as wide as body; snout moderately long with a fleshy keel at the tip, acuminate in dorsal view, protruding in profile; cranial crests absent; nostrils slightly protuberant, narrow, directed anterolaterally; canthus rostralis concave in dorsal view, rounded in cross section; loreal region slightly concave; upper eyelid with several rounded tubercles, larger posteriorly; tympanic annulus prominent, upper and posterolateral edge concealed by supratympanic fold; tympanic membrane distinct; two prominent, subconical postrictal tubercles surrounded by smaller ones. Choanae median, ovoid, not concealed by palatal shelf of maxillae; dentigerous processes of vomers small, prominent, oblique, moderately separated, positioned posteromedial to choanae; each vomer bearing several indistinct teeth; tongue longer than wide, posteriorly notched, posterior half free; vocal slits slightly curved, located at posterior half of mouth floor in between tongue and margin of jaw; vocal sac present.

Dorsal surfaces of body shagreen with scattered small tubercles; thin middorsal fold; one tubercle between nostrils and eyes, one interorbital, two postocular and two scapular tubercles forming an inverted “V”; skin on head and loreal region bearing small rounded tubercles; dorsolateral folds absent; evident lateral folds on anterior half of flanks; skin on flanks having more prominent and larger tubercles than on dorsum; skin on chest and belly coarsely areolate, that on throat shagreen, ventral surfaces of limbs smooth, ventral surfaces of thighs coarsely areolate; discoidal fold present. Low and rounded ulnar tubercles; outer palmar tubercle bifid, slightly bigger than ovoid thenar tubercle; subarticular and supernumerary tubercles at the base of fingers prominent, rounded; fingers bearing broad lateral fringes; Finger I shorter than Finger II; discs on Fingers broadly expanded, rounded; pads on fingers surrounded by circumferential grooves on all fingers (Fig. 9C).

Hindlimbs slender; dorsal surfaces of hindlimbs shagreen with scattered tubercles; posterior surfaces of thighs smooth, ventral surfaces of thighs coarsely areolate; heel bearing a median, prominent, subconical tubercle surrounded by indistinct tubercles; outer edge of tarsus bearing distinct median subconical tubercles; inner tarsal fold present; inner metatarsal tubercle ovoid, elevated, five times the size of round outer metatarsal tubercle; plantar surface with small but distinct supernumerary tubercles; subarticular tubercles prominent, rounded; toes bearing broad lateral fringes; basal webbing between toes IV and V present; discs on toes smaller than those on fingers, expanded, rounded; all toes having pads surrounded by circumferential grooves; relative lengths of toes: I < II < V < III < IV; Toe V much longer than Toe III (disc on Toe III reaches distal edge of penultimate subarticular tubercle on Toe IV, disc on Toe V reaches distal edge of distal subarticular tubercle on Toe IV; Fig. 9C). Coloration of the holotype in preservative is shown in Figure 33A; coloration in life unknown.

*Coloration of holotype in preservative*. Dorsal surfaces of body dark brown, dorsum with faint middorsal longitudinal line slightly darker than background coloration; brown supratympanic stripe; dorsal surfaces of thighs with cream transversal reticulations; groins, anterior and posterior surfaces of thighs brown with cream spots; ventral surfaces of body dusty cream, venter with a faint brown longitudinal midline and faint brown flecking (Fig. 33A).

*Coloration of holotype in life*. Unknown.

#### Variation.

**Figure 35.**
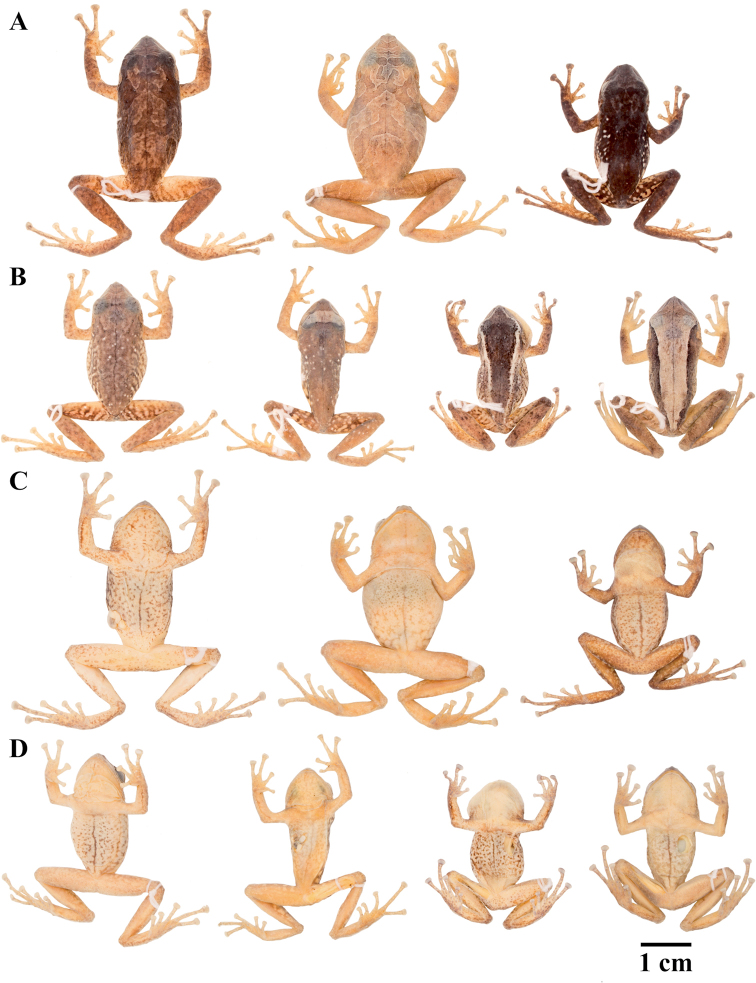
Color variation in preserved individuals of *Pristimantis totoroi* sp. nov. **A** Dorsal view of (from left to right): QCAZ 32039 (female), QCAZ 16918 (female), QCAZ 16834 (male) **B** Dorsal view of: QCAZ 16920 (male), QCAZ 25124 (juvenile female), QCAZ 25135 (male), QCAZ 31502 (male) **C** Ventral view of specimens in (**A**) **D** Ventral view of specimens in (**B**) See Suppl. material 2 for locality data. All specimens are shown at the same scale.

Based on the 43 specimens of the type series and photographs of five individuals. Variation of live and preserved individuals is shown in Figures 34, 35. Coloration in life is given in parenthesis. Dorsal coloration varies from light to dark brown (tan to dark brown); middorsal fold is dark brown to black. Dorsum may bear irregular chevrons, a broad middorsal band or parallel longitudinal stripes; scattered black flecks or white spots may be present on dorsum; head bears a black or brown supratympanic stripe, sometimes an interorbital band or stripe, and labial bars; flanks usually bear dark diagonal bars. Dorsal surfaces of thighs bear pale transversal bands, sometimes formed by rows of pale spots. Groins and hidden surfaces of thighs are cream to brown (reddish cream to brown) with or without small pale flecks or spots. Venter is cream to dusty cream with a brown midline extending from the belly to the throat; brown or gray flecks on belly, chest, and throat may be present (in males, throat varies from white to yellow). Iris is golden with a medial horizontal red streak and fine black reticulations; sclera varies from white to cream. Skin ornamentation, as middorsal fold, dorsolateral folds, and dorsal tubercles, may fade in preservative.

#### Advertisement call.

Based on recordings of QCAZ 25105 (December 14, 2001; 20h00), and a non-collected individual from the type locality (December 14, 2001; 21h40; 10.2 °C). The advertisement call consists of a series of 2–6 sharp peep-like notes (Fig. 6C); each of these notes lasts about 0.13 s (range 0.10–0.15 s) and is separated of the following note by 0.34 s (range 0.31–0.37 s). The peak time occurs exactly in the middle of the note. Fundamental frequency is the same as dominant frequency, on average 2895 Hz (range 2776–3013 Hz). Frequency increases along the note (2551–2620 Hz on average). Descriptive statistics for bioacoustic parameters are shown in Table 6.

#### Distribution, natural history, and conservation status.

*Pristimantis totoroi* is known from Western Montane Forest of Bolívar, Chimborazo and Cotopaxi Provinces, between 2258–3200 m (Fig. 2). This species occurs in primary and secondary forests and pastures. Individuals found at night were on low vegetation up to 1.6 m above the ground or inside bromeliads up to 3 m above the ground. During the day they were found only inside bromeliads. Calling males have been found in October, December, and January on low vegetation during the night.

Following the B1ab(iii) + 2ab(iii) IUCN criteria, we consider *P. totoroi* to be Endangered because: (i) it is only known from three localities (sensu IUCN 2017); (ii) its habitat is gradually degraded by human settlements, cattle raising, and agriculture; (iii) its Extent of Occurrence < 5000 km^2^ (346 km^2^); and (iv) its Area of Occupancy <500 km^2^ (28 km^2^). Its distribution overlaps with one small protected area, Cashca Totoras Protected Forest. Unfortunately, the protection of the forest is not enforced and, as a result, it is being destroyed by logging.

#### Etymology.

The specific epithet is a noun in the genitive case that refers to the type locality of this species, the Cashca Totoras Protected Forest. This small reserve contains Western Montane Forest and Paramo natural regions. It is one of few protected areas in the western slopes of the Andes of central Ecuador, which are part of a biodiversity hotspot. Therefore, its effective protection is urgent to preserve unique assemblages of Andean biodiversity.

#### Remarks.

*Pristimantis totoroi* has been mistakenly referred to as *P. phoxocephalus* (e.g., Hedges et al. 2008). Here, we recognize it as a different species and assign it to the *P. phoxocephalus* species group.

### Pristimantis verrucolatus sp. nov.

Taxon classificationAnimaliaAnuraStrabomantidae

40B938747BBF51959F1831816CD27B11

http://zoobank.org/AEA32DD5-E2E5-4B83-B0DD-098447EA3FFC

#### Common name.

English: Warty Flank Rain Frog Spanish: Cutín de flancos verrugosos

#### Holotype.

**Figure 36.**
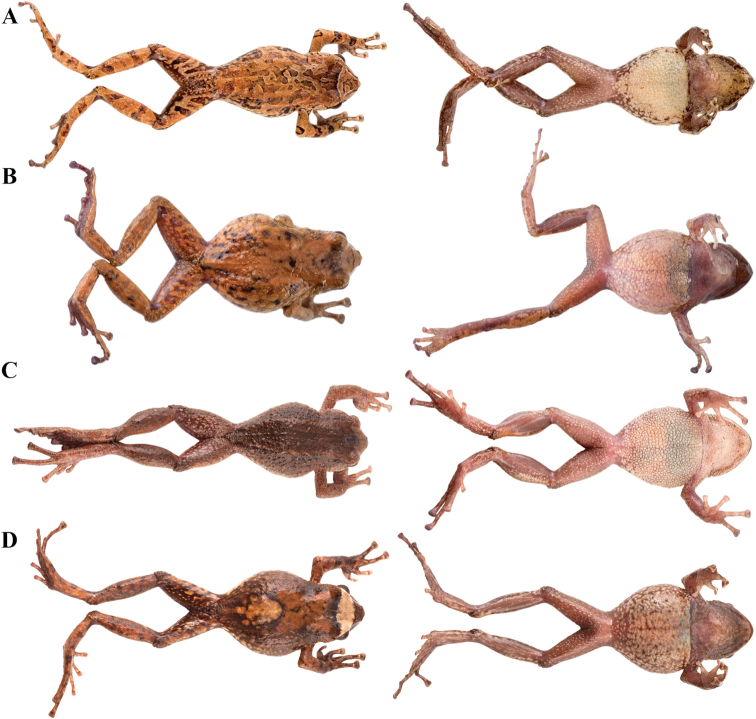
Color variation in live individuals of *Pristimantis verrucolatus* sp. nov. **A**QCAZ 46982 (holotype, male, SVL 28.4 mm) **B**QCAZ 45195 (male, SVL 34.5) **C**QCAZ 46993 (female, SVL 46.8 mm) **D**QCAZ 46999 (male, SVL 28.4 mm). Dorsal view on the left, ventral view on the right.

QCAZ 46982, an adult male from Yumate, Shoupshe, Azuay Province, Ecuador (2.7696S, 79.4254W, 3504 m), collected by Elicio E. Tapia on January 21, 2010. Figures 33B, 36A.

#### Paratypes

**(17: 15 males, 1 female, 1 juvenile).** Ecuador: Azuay Province: QCAZ 45174, adult female, QCAZ 45175, QCAZ 45195, adult males, from San Antonio, Cajas National Park border (2.8612S, 79.3787W, 2943 m), collected by Elicio E. Tapia and Juan Carlos Morocho in August 2009; QCAZ 46981, QCAZ 46984–989, adult males, collected with the holotype; QCAZ 46993–995, adult males, QCAZ 46992, juvenile, from Cochapamba (2.8020S, 79.4139W, 3662 m), collected by Silvia Aldás and Freddy Velásquez in January 2010; QCAZ 46999–7001, adult males, from Cochapamba (2.7971S, 79.4156W, 3548 m), collected by Elicio E. Tapia in January 2010.

#### Diagnosis.

A member of the *Pristimantis phoxocephalus* group characterized by the following combination of characters: (1) skin on dorsum shagreen with or without scattered tubercles, larger on posterior dorsum; with or without a faint middorsal fold; head with or without a middorsal row of two inconspicuous tubercles; flanks with warts and larger tubercles than those on dorsum; dorsolateral folds absent; thick, continuous or fragmented lateral folds on anterior flanks; skin on venter coarsely areolate; discoidal fold present or absent; (2) tympanic membrane and tympanic annulus prominent, its upper and posterior margin concealed by supratympanic fold; (3) snout moderately long, acuminate with a fleshy keel in dorsal view, protruding in profile; (4) upper eyelid with several low rounded tubercles; cranial crests absent; (5) dentigerous processes of vomers prominent, oblique, moderately separated, posteromedial to choanae; (6) males bearing vocal slits, vocal sac, and white nuptial pads; (7) Finger I shorter than Finger II; discs of digits broadly expanded, elliptical to truncate; (8) fingers with lateral fringes; (9) ulnar tubercles low and round sometimes connected by a weak fold; (10) heel bearing one or more small, prominent, subconical tubercles surrounded or not by some lower tubercles; outer and inner edge of tarsus bearing a row of low, rounded tubercles, inner tarsal fold present; (11) inner metatarsal tubercle ovoid, elevated, four times the size of round outer metatarsal tubercle; supernumerary tubercles numerous; (12) toes with broad lateral fringes; basal webbing present; Toe V longer or much longer than Toe III (disc on Toe III reaches the proximal to distal edge of penultimate subarticular tubercle on Toe IV, disc on Toe V reaches the proximal to distal edge of distal subarticular tubercle on Toe IV); toe discs smaller than those on fingers, elliptical to truncate (Fig. 9D); (13) in life, dorsum light to dark brown; head with an interorbital stripe or band and a dark supratympanic stripe; venter cream to white with or without brown reticulations; groins and posterior surfaces of the thighs reddish brown with small light brown, orangey brown or yellow spots; iris coppery brown (Fig. 36); (14) average SVL in adult females: 44.0 ± 4.5 mm (40.4–47.5 mm; *n* = 2); in adult males: 29.4 ± 2.7 mm (25.1–34.5 mm; *n* = 15).

#### Comparison with other species.

*Pristimantis verrucolatus* is most similar to *P. atillo*, *P. jimenezi*, *P. phoxocephalus*, *P. teslai*, *P. torresi*, and *P. totoroi*. It differs from all of them by having thick lateral folds. Except for *P. teslai*, it is the only having large tubercles or warts on flanks; though tubercles on flanks are also present in *P. teslai*, they are not as large as the ones of *P. verrucolatus*; furthermore, dorsum of *P. teslai* is tuberculate, while the dorsum *P. verrucolatus* is shagreen, with or without scattered tubercles. *Pristimantis verrucolatus* is further distinguished from *P. atillo* by the coloration of its groins (orange in *P. atillo*; reddish brown with light brown to yellow spots in *P. verrucolatus*), and its larger size (males *Z* = 2.35, *p* = 0.0188, SVL = 23.4–27.3 mm in *P. teslai*, 25.1–34.5 mm in *P. verrucolatus*). *Pristimantis jimenezi*, which has an adjacent distribution at lower elevations, is smaller than *P. verrucolatus* (males *Z* = 3.58643, *p* = 0.0003, SVL = 21.8–27.0 mm in *P. jimenezi*, 25.1–34.5 mm in *P. verrucolatus*; females *Z* = 2.00347, *p* = 0.0451, SVL = 31.1–37.4 mm in *P. jimenezi*, 40.4–46.8 mm in *P. verrucolatus*). Additionally, the advertisement call of *P. verrucolatus* is very distinctive, distinguishing it from all species with available calls: *P. jimenezi*, *P. phoxocephalus*, and *P. totoroi*. The call of *P. verrucolatus* has a single note, longer than those of the calls of the other species. It also has the lowest dominant frequency and the highest variation in frequency from the beginning to the end of the note (Table 6). *Pristimantis* sp. (CCS1) is the closest species to *P. verrucolatus* (average *p* genetic distance = 3.2%), they are not morphologically alike. CCS1 has a chubbier body, lacks a keel at the tip of the snout (present in *P. verrucolatus*), has slightly expanded discs on fingers and toes (broadly expanded in *P. verrucolatus*), and lacks lateral folds (present in *P. verrucolatus*).

#### Description of the holotype.

QCAZ 46982, SC29952 adult male. Measurements in mm: SVL 28.4; TL 13.3; FL 12.9; HL 8.8; HW 9.8; ED 3.3; TD 1.4; IOD 2.7; EW 2.5; IND 2.2; EN 2.6; TED 1.0. Head wider than body, wider than long; snout moderately long, acuminate with a fleshy keel in dorsal view, protruding in profile; nostrils slightly protuberant, ovoid, directed dorsolaterally; canthus rostralis weakly concave in dorsal view, angular in cross section; loreal region concave; cranial crests absent; upper eyelid bearing several low rounded tubercles; tympanic membrane and annulus prominent, its upper and posterior margins concealed by supratympanic fold; two enlarged, prominent and rounded postrictal tubercles surrounded by smaller tubercles. Choanae large, rounded, not concealed by palatal shelf of maxillae; dentigerous processes of vomers prominent, oblique, moderately separated, posteromedial to choanae; each vomer bearing several teeth; tongue slightly longer than wide, posterior border notched; posterior three fifths free; vocal slits slightly curved, located at posterior half of mouth floor in between tongue and margin of jaw; vocal sac present.

Skin on dorsum shagreen with scattered low tubercles; thin middorsal fold; thick lateral folds; flanks with large warts; skin on venter coarsely areolate. Chest, throat, and ventral surfaces of thighs areolate; discoidal fold absent. Low and round ulnar tubercles connected by an ill-defined fold; white nuptial pads present; palmar tubercles prominent, outer palmar tubercle bifid, slightly bigger than ovoid thenar tubercle; subarticular tubercles prominent, rounded; supernumerary tubercles at base of fingers prominent, smaller than subarticular tubercles; fingers with lateral fringes; Finger I shorter than Finger II; discs on fingers broadly expanded, elliptical; ventral pads on fingers surrounded by circumferential grooves (Fig. 9D).

Hindlimbs slender; dorsal surfaces of hindlimbs with scattered low tubercles; posterior surfaces of thighs smooth, ventral surfaces of thighs areolate; heel bearing a small subconical tubercle surrounded by some lower rounded tubercles; outer edge of tarsus bearing large low subconical tubercles; inner tarsal fold present followed by small distinct round tubercles on inner edge of tarsus; inner metatarsal tubercle ovoid, elevated, 4 × the size of round outer metatarsal tubercle; distinct, round supernumerary tubercles at the base of toes, indistinct ones on the rest of plantar surface; subarticular tubercles prominent, rounded; toes with broad lateral fringes; basal webbing present; discs on toes expanded, elliptical, smaller than those on fingers; all toes having ventral pads surrounded by circumferential grooves; relative lengths of toes: I < II < III < V < IV; Toe V much longer than Toe III (disc on Toe III reaches distal edge of the penultimate subarticular tubercle on Toe IV, disc on Toe V reaches distal edge of distal subarticular tubercle on Toe IV; Fig. 9D). Coloration in life and preservative is shown in Figures 33B and 36A.

*Coloration of holotype in preservative*. Dorsum gray with light gray reticulations bordered by black lines; black interorbital and supratympanic stripes, and black canthal and labial bars; dorsal surfaces of limbs light gray with dark transversal bands bearing scattered black flecks; flanks with oblique light gray reticulations bordered by black spots; groins and posterior surfaces of thighs dark brown with cream spots; venter white; ventral surfaces of limbs dusty cream; throat dusty cream with dark brown mottling near the lips (Fig. 33B).

*Coloration of holotype in life*. Based on studio photographs. Dorsum brown with light brown reticulations bordered by black lines; black interorbital and supratympanic stripes, and black canthal and labial bars; dorsal surfaces of limbs light brown bearing dark brown transverse bands with scattered black flecks; flanks with oblique light brown reticulations bordered by black spots; groins and posterior surfaces of thighs dark brown with small cream spots; venter white; throat yellowish cream, vocal sac and ventral surfaces of limbs dusty pinkish cream; iris coppery brown with thin black reticulations; white sclera (Fig. 36A).

#### Variation.

**Figure 37.**
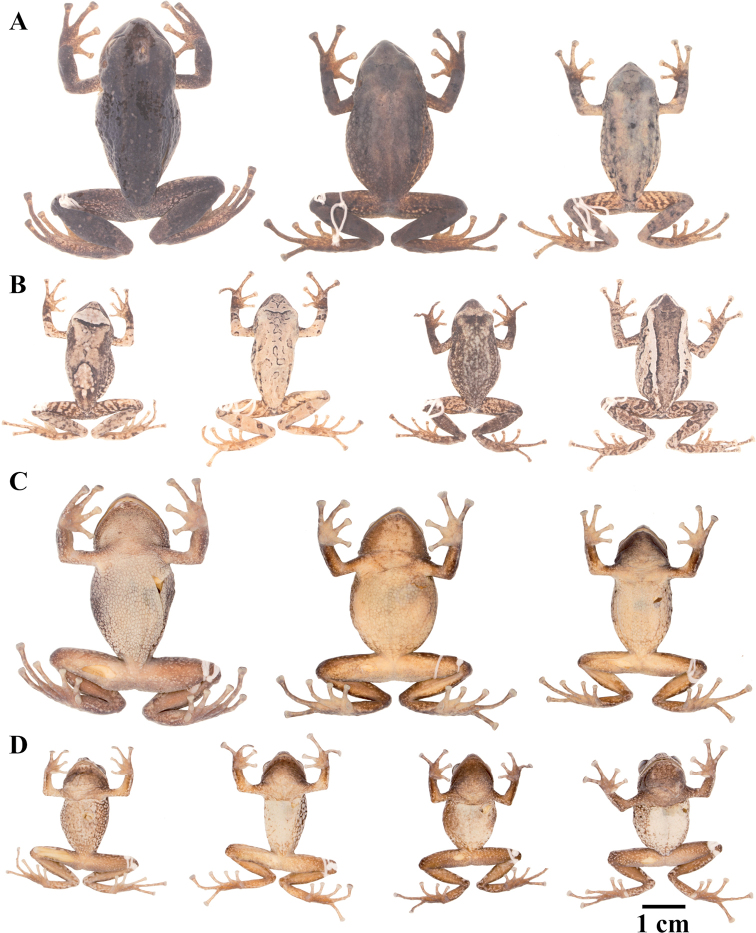
Color variation in preserved individuals of *Pristimantis verrucolatus* sp. nov. **A** Dorsal view of (from left to right): QCAZ 46993 (female), QCAZ 45174 (female), QCAZ 45195 (male) **B** Dorsal view of: QCAZ 46999 (male), QCAZ 46987 (male), QCAZ 46984 (male), QCAZ 46981 (male) **C** Ventral view of specimens in (**A**) **D** Ventral view of specimens in (**B**) See Suppl. material 2 for locality data. All specimens are shown at the same scale.

Data are based on 18 preserved specimens and photographs from six living individuals. Variation in life and preservative is shown in Figures 36, 37. Coloration in life is given in parenthesis. Dorsal coloration varies from light gray to dark gray or brown (light to dark brown). Dorsal pattern can be uniformly colored, with irregular reticulations or with a middorsal band. Light colored individuals bear a pale stripe on lips. Head usually bears a dark canthal stripe that continues along the upper eyelid and above the tympanum, and an interorbital stripe. Most specimens have labial bars. Flanks bear or not pale reticulations or black flecks. Groins and posterior surfaces of thighs are brown with cream dots (reddish brown with small light brown or orangey brown spots). Ventral surfaces of limbs are dusty cream to brown; venter is cream or white with or without brown reticulations; throat is cream to dusty brown with or without dark mottling. Iris is coppery brown with thin black reticulations; white sclera. Flanks have medium sized to large tubercles and warts, arranged or not in rows; lateral folds can be thick and continuous or be present as a row of tubercles.

#### Advertisement call.

Based on recordings of QCAZ 46981 (September 21, 2010; 19h00; 8.8 °C) and QCAZ 46984 (January 20, 2010; 21h10). Advertisement calls consist of a single sharp whistle (Fig. 6D); each lasting on average 0.43 s (range 0.41–0.46 s). The peak time of each note occurs exactly in the middle of its duration. The dominant and fundamental frequencies are the same, on average 2144 Hz (range 1927–2302 Hz). The note increases its frequency from beginning to end (2231–1838 Hz on average). Descriptive statistics for bioacoustic parameters are shown in Table 6.

#### Distribution, natural history, and conservation status.

This species is known from Western Andean slopes of Azuay Province, between 2943–3662 m a.s.l (Fig. 2). It inhabits Paramo and Western Montane Forest regions. Individuals collected during the day were found beneath rocks or inside arboreal bromeliads up to 2 m above ground; at night they were found active on branches of low shrubs, up to 80 cm above the ground, or inside bromeliads. Gravid females were found in August and January; calling males in January.

*Pristimantis verrucolatus* is known from only two localities (sensu IUCN) and has a very restricted distribution. These places are adjacent to Parque Nacional Cajas, a protected area with unexplored regions that represent potential distribution for the species. Thus, following the IUCN (2017) guidelines, we assign this species to the Data Deficient Red List category.

#### Etymology.

The specific epithet is a noun in apposition with masculine gender. It is derived from the Latin words *verruca* meaning wart, and *latus* meaning flanks. The name refers to the large and low tubercles or warts on flanks that characterize this species.

##### *Pristimantis cryptomelas* species group new taxon

**Definition.** Monophyly of the *P. cryptomelas* species group is strongly supported in our phylogeny. Members of this group are characterized by: (i) postocular folds present; (ii) dorsolateral folds absent; (iii) cranial crests absent (except for low crests in *P. spinosus*); (iv) tympanic membrane and tympanic annulus prominent; (v) eyes bearing prominent tubercles; (vi) dentigerous processes of vomer present; (vii) prominent tubercles on heel and tarsus; (viii) fingers and toes with lateral fringes; (ix) broadly expanded discs on fingers and toes; (x) basal webbing between toes present; (xi) in life, groins and concealed surfaces of thighs have distinctive coloration patterns, including flash colors, and light or bright colored flecks or spots on a darker background; colors, shapes, and sizes of these ornaments are variable among species; (xii) SVL females 25.9–46.1 mm; SVL males 16.1–30.3 mm.

**Content.** The *P. cryptomelas* species group comprises four described species, *P. cryptomelas*, *P. gagliardoi*, *P. muscosus*, and *P. spinosus*, and the newly described *P. nangaritza* sp. nov. We suspect that the species content of the group will increase with collections and genetic studies of populations from northern Peru.

**Distribution.** Members of the *P. cryptomelas* group occur in the eastern Andean slopes of southern Ecuador and northern Peru. In Ecuador, they inhabit the Eastern Foothill and Montane Forest of Cañar, Loja, Morona Santiago, and Zamora Chinchipe Provinces, between elevations of 1800 and 3500 m a.s.l. In Peru, they live between 1770 and 2820 m a.s.l. Distribution based on QCAZ collection specimens, Duellman and Lehr (2009), and Yánez-Muñoz et al. (2012).

**Remarks.** The *P. muscosus* specimen included in our phylogeny is from the same and only population of *P. muscosus* reported for Ecuador (Yánez et al. 2012). The population is 216 km from the type locality in Peru (east slope of Abra Pardo de Miguel). Our results indicate that most species of *Huicundomantis* have small geographic ranges. The large geographic distance from the type locality of *P. muscosus* suggests that the Ecuadorian population may represent an undescribed species. The same applies to Peruvian populations of *P. cryptomelas*, which are widely separated from the type locality in Ecuador.

### Pristimantis nangaritza sp. nov.

Taxon classificationAnimaliaAnuraStrabomantidae

2E237460391E5375A410AFD27EC11A21

http://zoobank.org/E35A79CF-16A8-4838-90BA-103D0C48A019

#### Common name.

English: Nangaritza Rain Frog. Spanish: Cutín de Nangaritza.

#### Holotype.

**Figure 38.**
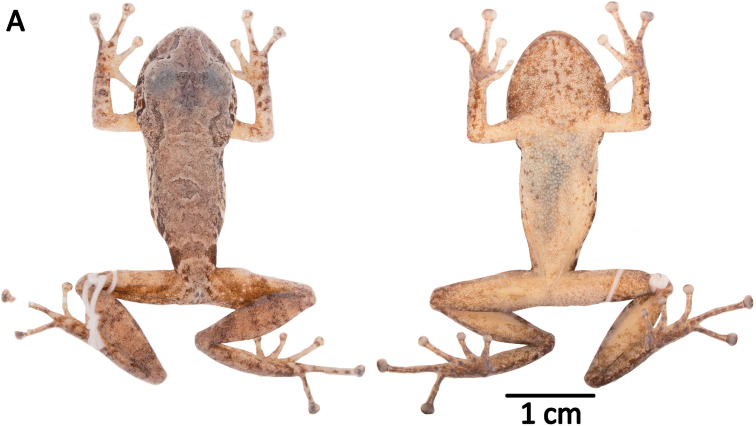
Holotype of *Pristimantis nangaritza* sp. nov. Photographs of preserved holotype of **A***P. nangaritza* (QCAZ 41710, female). Dorsal view on the left, ventral view on the right.

QCAZ 41710, an adult female from Alto Nangaritza Protected Forest, Las Orquídeas, Tepuy Forest, Zamora Chinchipe Province, Ecuador (4.2620S, 78.6902W, 1809 m), collected by Elicio E. Tapia on April 18, 2009. Figure 38.

#### Paratypes

**(17: 13 males, 3 females, 1 juveniles).** All from Alto Nangaritza Protected Forest, Las Orquídeas, Tepuy Forest. Ecuador: Zamora Chinchipe Province: QCAZ 41704–708, QCAZ 41725–726, QCAZ 41728–729, QCAZ 41731–733, adult males, QCAZ 41734–736, adult females, QCAZ 41730, juvenile (4.2632S, 78.6911W, 1843 m), collected by Elicio E. Tapia, Jessica Loe Deichmann, Amable Fermín Jiménez and Holger Braun in April 2009; QCAZ 41709, adult male (4.2567S, 78.6780W, 1820 m), collected by Elicio E. Tapia and Jessica Loe Deichmann in April 2009.

#### Diagnosis.

**Figure 39.**
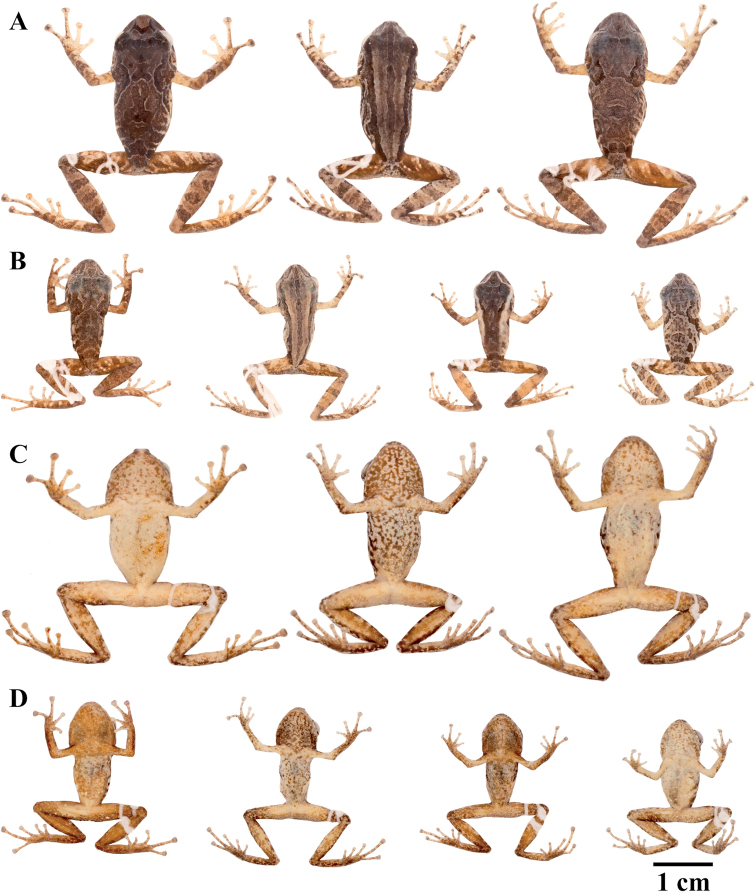
Color variation in preserved individuals of *Pristimantis nangaritza* sp. nov. **A** Dorsal view of (from left to right): QCAZ 41730 (juvenile female), QCAZ 41735 (female), QCAZ 41736 (female) **B** Dorsal view of: QCAZ 41708 (male), QCAZ 41732 (male), QCAZ 41728 (male), QCAZ 41725 (male) **C** Ventral view of specimens in (**A**) **D** Ventral view of specimens in B. See Suppl. material 2 for locality data. All specimens are shown at the same scale.

A species of the *Pristimantis cryptomelas* group with the following combination of characters: (1) dorsal surfaces finely tuberculate; middorsal fold present or absent; head with a middorsal row of two small tubercles; dorsolateral folds absent; thin lateral folds on anterior half of flanks present or absent; skin on venter coarsely areolate; discoidal fold present or absent;) (-shaped postocular folds; (2) tympanic membrane and tympanic annulus prominent, its upper and posterior margin concealed by thick supratympanic fold; (3) snout moderately long, subacuminate in dorsal view, rounded in profile; (4) upper eyelid with subconical tubercles surrounded by several smaller tubercles; cranial crests absent; (5) dentigerous processes of vomers prominent, oblique, narrowly to broadly separated, posteromedial to choanae; (6) vocals slits and white nuptial pads present in adult males; vocal sac not externally expanded; (7) Finger I shorter than Finger II; discs of digits broadly expanded, rounded to elliptical; (8) fingers with lateral fringes; (9) low ulnar tubercles; (10) heel bearing a subconical tubercle surrounded by smaller tubercles; inner and outer edge of tarsus bearing low tubercles; short inner tarsal fold; (11) inner metatarsal tubercle ovoid, elevated five times the size of round outer metatarsal tubercle; supernumerary tubercles numerous; (12) toes with lateral fringes; basal webbing barely evident; Toe V longer or much longer than Toe III (disc on Toe III reaches the middle or exceeds distal edge of penultimate subarticular tubercle on Toe IV, disc on Toe V reaches the middle or exceeds distal edge of distal subarticular tubercle on Toe IV); toe discs smaller than those on fingers, elliptical (Fig. 8C); (13) in preservative, dorsum light to dark brown with darker markings including a scapular W, irregular chevrons and interorbital stripe, or a pattern of longitudinal stripes; head with black or dark brown supratympanic, canthal and labial vertical bars; flanks with oblique pale bars that merge with ventral coloration; posterior surfaces of thighs light brown with or without minute pale flecks; groins with the same coloration as posterior surfaces of thighs or venter; venter and throat cream with different levels of brown mottling (Fig. 39); (14) average SVL in adult females: 29.1 ± 2.7 mm (25.9–32.4 mm; *n* = 4); in adult males: 18.8 ± 1.1 mm (17.4–20.5 mm; *n* = 13).

#### Comparison with other species.

It is most similar to *P. cryptomelas*, *P. gagliardoi*, *P. muscosus*, *P. spinosus*, and *P. versicolor*. The postocular folds of *P. nangaritza* are lower than those of *P. cryptomelas*, the background color of the posterior surfaces of thighs is light brown instead of black, its body is smaller (Table 5), and males have vocal slits (absent in males of *P. cryptomelas*). *Pristimantis gagliardoi* can be distinguished from *P. nangaritza* by having W-shaped postocular folds (“) (”-shaped in *P. nangaritza*), smaller tympanum (Table 5), and by lacking vocal slits. *Pristimantis muscosus* has smaller tubercles on the upper eyelid (round in *P. muscosus*; subconical in *P. nangaritza*), lacks tubercles on the inner edge of tarsus (present in *P. nangaritza*), and have dark brown or black groins. *Pristimantis spinosus* differs from *P. nangaritza* in having low cranial crests (absent in *P. nangaritza*) and black posterior surfaces of thighs with white spots. *Pristimantis versicolor* is readily recognized from *P. nangaritza* by lacking postocular folds and having a proportionally larger tympanum (females *Z* = 2.16506, *p* = 0.0304 TD/SVL = 4.7–5.9% in *P. nangaritza*, 5.2–7.2% in *P. versicolor*; males *Z* = 3.45394, *p* = 0.0006, TD/SVL = 5.1–5.4% in *P. nangaritza*, 5.8–6.4% in *P. versicolor*).

#### Description of the holotype.

An adult female (QCAZ 41710, SC28142). Measurements (in mm): SVL 32.4; TL 15.5; FL 13.4; HL 12.2; HW 12.1; ED 3.7; TD 1.7; IOD 3.1; EW 3.5; IND 2.5; EN 3.7; TED 1.2. Head longer than wide, wider than body; snout moderately long, subacuminate in dorsal view, rounded in profile; cranial crests absent; nostrils slightly protuberant, narrow, directed laterally with slight dorsal inclination; canthus rostralis concave in dorsal view, sharp in cross section; loreal region slightly concave; upper eyelid with subconical tubercles, those on posterior half larger; tympanic membrane distinct; tympanic annulus prominent, its upper and posterior edge concealed by supratympanic fold; two small prominent postrictal tubercles. Choanae large, circular, not concealed by palatal shelf of maxillae; dentigerous processes of vomers prominent, oblique, narrowly separated, positioned posteromedial to choanae; each vomer bearing several distinct teeth; tongue as long as wide, posteriorly notched; posterior third not adherent to the floor of mouth.

Dorsal surfaces of body finely tuberculate; dorsolateral folds absent; bearing) (-shaped postocular folds; skin on venter coarsely areolate, ventral surfaces of limbs smooth, ventral surfaces of thighs coarsely areolate; weak discoidal fold. Low median ulnar tubercles; outer palmar tubercle bifid, twice the size of ovoid thenar tubercle; subarticular tubercles prominent, rounded; prominent supernumerary tubercles at the base of fingers, slightly smaller than subarticular tubercles; fingers bearing lateral fringes; Finger I shorter than Finger II; discs on fingers broadly expanded, rounded; pads on fingers surrounded by circumferential grooves on all fingers (Fig. 8C).

Hindlimbs slender; dorsal surfaces of hindlimbs finely tuberculate; posterior surfaces of thighs smooth, ventral surfaces of thighs coarsely areolate; heel bearing a medium sized, subconical tubercle surrounded by several smaller tubercles; outer and inner edge of tarsus bearing low tubercles; inner tarsal fold present; inner metatarsal tubercle ovoid, elevated 5 × the size of round outer metatarsal tubercle; supernumerary tubercles prominent at the base of each toe, those on plantar surface distinct, but low; subarticular tubercles prominent, rounded; toes bearing lateral fringes; basal webbing barely evident between toes IV and V; discs on toes smaller than those on fingers, expanded and elliptical; all toes having pads surrounded by circumferential grooves; relative lengths of toes: I < II < III < V < IV; Toe V longer than Toe III (disc on Toe III exceeds distal edge of penultimate subarticular tubercle on Toe IV, disc on Toe V exceeds the distal of distal subarticular tubercle on Toe IV; Fig. 8C). Color of the holotype in preservative is shown in Figure 38; coloration in life is unknown.

*Coloration of holotype in preservative*. Dorsum light brown with lighter irregular reticulations and a W-shaped scapular mark bordered by dark brown lines; head with dark brown canthal, supratympanic, and labial bars; flanks with cream oblique cream bars bordered by dark brown spots and lines; groins, and concealed surfaces of thighs dark brown with scattered cream flecks; venter cream and ventral surfaces of thighs cream; throat cream with brown mottling; ventral surfaces of limbs dusty cream (Fig. 38).

*Coloration of holotype in life*. Unknown.

#### Variation.

This section is based on 18 specimens of the type series. In preservative, dorsum varies from light to dark brown with darker markings including a scapular W, irregular chevrons, and interorbital stripe. Some individuals have a pattern of longitudinal parallel stripes. Posterior surfaces of thighs are brown with or without minute pale flecks. Groins with the same coloration as posterior surfaces of thighs or venter. Venter cream with varying amount of brown mottling. This variation is shown in Figure 39. Coloration in life is unknown.

#### Distribution, natural history, and conservation status.

*Pristimantis nangaritza* is only known from its type locality, Alto Nangaritza Protected Forest, Zamora Chinchipe Province, Ecuador, a low vegetation forest with bromeliads, orchids, moss, and *Podocarpus* trees that belongs to the Eastern Foothill Forest, between 1809 and 1843 m a.s.l. (Fig. 1). Individuals were found active at night on branches, 1–2 m above ground. Calling males were found in April.

We consider it as a Data Deficient species because adjacent areas in Alto Nangaritza Protected Forest are difficult to access and poorly explored.

#### Etymology.

The specific epithet refers to the type locality of this species, Alto Nangaritza Protected Forest. This protected area preserves native vegetation of the Cordillera del Cóndor and hosts unique geologic formations in Ecuador, called Tepuyes. This reserve is largely unexplored and is currently threatened by the potential opening of roads for mining activities; 80% of its territory is under mining concessions.

## On the identity of *Pristimantis riveti*

Several species described here were previously misidentified as *P. riveti*, including *P. lutzae*, *P. gloria*, *P. multicolor*, *P. chomskyi*, and *Pristimantis* sp. (CCS1) (e.g., Almendáriz and Orcés 2006; Heinicke et al. 2007; Padial et al. 2014). These misidentifications were based on a redescription of “*P. riveti*” by Lynch (1979). Populations used for Lynch’s redescription (Azuay Province, Cuenca) are geographically distant (400 km to the south) from the type locality of *P. riveti*, “Mirador”, which, based on the collector itinerary, we infer to be in Carchi Province, Ecuador. Although Despax (1911) did not mention the province for the type locality, the holotype was collected by Paul Rivet during the Second Geodesic Mission which visited “Mirador” in Carchi Province (Perrier 1913). According to Frost (2019), Mirador is in Tungurahua Province, Ecuador, but we could not find a reference to support that statement.

The great distance between the type locality of *P. riveti* and populations in Azuay Province used in Lynch’s redescription indicate that they are not conspecific. This conclusion is consistent with morphological comparisons of photographs of the holotype of *P. riveti*, MNHNP 1902.357 (Fig. 23B) with *P. lutzae* (= “*P. riveti*” of Lynch 1979) which showed marked differences. The holotype of *P. riveti* is an adult female whose snout-vent length is 34.2 mm, a size above the range for females of *Pristimantis lutzae* (29.7–33.9 mm); *Pristimantis riveti* has distinctive big white irregular blotches dispersed over the dorsal surface, which are absent in *P. lutzae*; besides, Toe V is slightly longer than toe III in *P. riveti* while it is longer or much longer than Toe III in *P. lutzae*. The combined evidence indicates that the populations from Azuay usually ascribed to *P. riveti* represent a previously undescribed species that we have named here.

Based on information published by Perrier (1913) we were able to infer the position of the type locality of *P. riveti*. Mirador is a mountain peak at an elevation of 3830 m near the border between Carchi and Sucumbíos Provinces. Recent collections made on that mountain, at an elevation between 3500 and 3792 m, include specimens similar to the holotype of *P. riveti* (QCAZ 69853, 69855, 69857–859, 69861, 69865–869). We tentatively considered these specimens to be the only documented records of *P. riveti* sensu stricto besides that of the holotype.

## Discussion

### Cryptic diversity within *Huicundomantis*

Herein, we document the existence of 16 new candidate species within a clade of which only 12 species were previously described. These results represent an increase in species richness of 133%. Moreover, several populations not included in our study likely represent additional undescribed species. For example, in the QCAZ collection, specimens from 6 km E Loja, Loja Province (e.g., QCAZ 21708) or Zamorahuaico, Zamora Chinchipe Province (e.g., QCAZ 22529) are morphologically similar to *P. phoxocephalus* and appear to represent undescribed species. These populations were excluded from our review because they lacked tissues for DNA analyses. Similarly, several populations of “*P. phoxocephalus*” from southern Ecuador reported by Lynch (1979) (e.g., 10 km SW Victoria del Portete, Azuay Province; 18.4 km NW El Tambo, Cañar Province; Saraguro, Loja Province) and from northern Peru by Duellman and Lehr (2009), Duellman and Pramuk (1999), and Duellman and Wild (1993) are likely undescribed species as indicated by our samples of “*P. phoxocephalus*” from Peru which fall outside *Huicundomantis*. Given the branch lengths that separate them, the three Peruvian samples appear two represent two different species (Suppl. material 3). Our results suggest that Andean *Pristimantis* still hide a large number of cryptic undescribed species. If the content of undescribed species of our group is a representative sample of all *Pristimantis*, we would expect the discovery of hundreds of new species in the coming decades.

Characters that help diagnose species of *Huicundomantis* are body tuberculation, the presence of dermal folds (e.g., middorsal, lateral, postocular folds), and the coloration in life of the groins, concealed surfaces of thighs, and iris. These characters are frequently lost in preservative, which could explain why previous taxonomic reviews, mainly based on morphology of preserved specimens, lumped so many species into single binomens. Hence, we highlight the importance of documenting coloration and skin texture in live individuals, prior to preservation. We also found extensive intraspecific and even intrapopulation morphological variation, especially in coloration and skin texture (e.g., *P. multicolor*; Fig. 24). This variation can only be properly described by sampling a large number of individuals. Future taxonomic reviews of *Pristimantis* will greatly benefit from documenting both life coloration and genetic variation in a large number of specimens.

Morphometrics were of little help to diagnose species except for several species pairs. For morphometric diagnoses, we compared body size and head proportions, which can be useful taxonomic characters (Arroyo et al. 2005). The addition of other measurements or the use of new approaches such as geometric morphometrics (Kaliontzopoulou 2011; Acevedo et al. 2016) could help to better differentiate cryptic species of *Huicundomantis*.

Similar to morphometrics, environmental envelopes were of limited help to distinguish closely related species. However, we were able to discriminate among species inhabiting high-altitude Paramo habitats from those inhabiting warmer regions in Eastern Foothill Forest, Deciduous Costa Forest, and Western Foothill Forest. These environmental differences are mirrored by distinct body types that appear to be related to different environments. Species from paramo habitats have a chubby appearance and usually have narrow finger discs while species from lower altitudes have a slenderer body and wider discs.

When available, advertisement calls allowed unequivocally diagnosing of closely related species. This is congruent to previous studies in *Pristimantis* (Hutter and Guayasamin 2015), and other Neotropical frogs (e.g., Padial et al. 2008; Funk et al. 2012; Caminer et al. 2014,) in which divergence in advertisement calls led to the discovery of cryptic species. Advertisement calls are under sexual selection and were the most variable trait between species. Therefore, it seems likely that sexual selection played an important role in speciation of *Huicundomantis*.

### *Huicundomantis*, a new subgenus for *Pristimantis*

We propose *Huicundomantis* as a subgenus given that it has strong phylogenetic support and morphological distinctiveness. Currently, two subgenera are recognized within the genus *Pristimantis*: *Pristimantis* and *Hypodictyon*. According to recent reviews, *Pristimantis* is paraphyletic while *Hypodictyon* (sensu Crawford et al. 2010) is monophyletic (Heinicke et al. 2017). We agree with Heinicke et al. (2017) on the need for the recognition of new subgenera within *Pristimantis* to convey phylogenetic information among its 532 species. Padial et al. (2014) did not recognize the subgenus Pristimantis given its rampant paraphyly. However, the recognition of *Hypodictyon* is justified given its well established monophyly. The description of the new monophyletic subgenus Huicundomantis is an additional step to reach the goal of splitting *Pristimantis* into monophyletic subgenera. Moreover, *Huicundomantis* does not generate taxonomic instability because the subgenus Pristimantis has been largely ignored as a result of its nearly complete redundancy with its parent genus. Finally, we highlight that, if needed, the species content of *Huicundomantis* can be redefined to accommodate the requirements of a large-scale taxonomic review of its parent genus.

The name *Huicundomantis* refers to the association of most of its species to bromeliad plants (*huicundos* in Quechua). This association has been reported, at least facultatively, in *P. atillo*, *P. atratus*, *P. chomsky*, *P. cryptomelas*, *P. jimenezi*, *P. multicolor*, *P. phoxocephalus*, *P. tinguichaca*, *P. torresi*, *P. totoroi*, *P. verrucolatus*, and *P. versicolor* (Ron et al. 2019 and references therein). Another clade of *Pristimantis* with a large number of species associated to bromeliads is the *P. lacrimosus* group (Hedges et al. 2008). Both clades are not closely related (Pyron 2014), which suggests that the bromeliad association evolved independently in both groups. Numerous other species of *Pristimantis* of uncertain phylogenetic position also occur in bromeliads (e.g., *P. huicundo*, *P. orphnolaimus*). Understanding the evolution origin of this ecological character will require a comprenhensive analysis across all species of *Pristimantis*.

### Speciation and genetic divergence

We found seven sister species pairs (including unconfirmed candidate species) within *Huicundomantis*. In all of them, their distribution ranges do not overlap and do not have significant differences in environmental envelope. Allopatric distribution and lack of differences in environmental envelope suggest that speciation has been mainly driven by incidental divergence in allopatry, rather than ecological divergence along environmental gradients (Peterson et al. 1999; Graham et al. 2004). The fragmented landscape of the Andean slopes, and expansions and contractions of the distribution range due to environmental oscillations should frequently result in geographic isolation, making allopatric speciation the most likely mode of speciation for *Huicundomantis*. Moreover, Andean slopes allow the existence of high diversity and endemism because they present sharp gradients of elevation, temperature, and precipitation. These heterogeneous conditions should explain the small distribution ranges observed in montane species of frogs (Wollenberg et al. 2008). The pattern of species richness and diversification of *Huicundomantis* is congruent with other studies from mountainous areas, which report allopatric speciation as the most frequent mode of speciation in the group (e.g., Lynch and Duellman 1997; Coloma et al. 2012; Jungfer et al. 2013).

We found that the 3% genetic distance threshold (gene 16S) proposed to identify candidate species (Fouquet et al. 2007; Vieites et al. 2009) could be too conservative. In *Huicundomantis*, the minimum genetic distance between confirmed candidate species was 2.0%; applying the 3% threshold would have resulted in 3 confirmed candidate species overlooked. Our results are similar to those of other systematic reviews of Neotropical amphibians (e.g., Padial and de la Riva 2009; Coloma et al. 2012; Funk et al. 2012; Jungfer et al. 2013; Caminer and Ron 2014; Ortega et al. 2015) showing that the 3% genetic distance threshold could result in underestimation of species numbers.

### Cryptic diversity and conservation

The discovery of cryptic diversity can impact estimates of the conservation status of species (Bickford et al. 2007). One of the main variables to assess the conservation status of species is the size of its distribution range (IUCN 2017). After dividing what was thought to be a single species into several cryptic species, distribution ranges of the resulting species are smaller than those of the original; therefore, the conservation status should be revaluated after cryptic species are discovered. For example, until now, *Pristimantis phoxocephalus* was considered to be distributed along the western and eastern Andean slopes of Ecuador and Peru (elevations 1800–3100 m a.s.l; Rodríguez et al. 2004). Given its large distribution range, it was considered Least Concern by the IUCN Red List (Rodríguez et al. 2004). Our review shows that *P. phoxocephalus* is known from a single locality, Pilaló, in the western Andean slopes of central Ecuador. Therefore, we propose that *P. phoxocephalus* is Critically Endangered according to the IUCN (2017) guidelines.

Accurate taxonomic assessments contribute to conservation by helping to delimit priority conservation areas. Species of *Huicundomantis* are distributed along the central and southern Andes of Ecuador between 230 and 4200 m of elevation. Sixteen out of 28 species occur in protected areas: *P. atillo* (Sangay National Park), *P. atratus* (Podocarpus National Park, Tapichalaca Reserve, Yacuri National Park), *P. chomskyi* (Tapichalaca Reserve), *P. cryptomelas* (Huashapamba Protected Forest, Podocarpus National Park, Tapichalaca Reserve, Yacuri National Park), *P. gagliardoi* (Sangay National Park, Mazar Wildlife Reserve), *P. hampatusami* (Buenaventura Reserve), *P. lutzae* (Cajas National Park, Mazar Wildlife Reserve, Yanuncay-Irquis Protected Forest, Mazán Protected Forest, Sangay National Park), *P. multicolor* (Yacuri National Park), *P. muscosus* (Tapichalaca Reserve), *P. nangaritza* (Alto Nangaritza Protected Forest), *P. philipi* (Cajas National Park), *P. tinguichaca* (Sangay National Park), *P. totoroi* (Cashca Totoras Protected Forest), *P. versicolor* (Podocarpus National Park, Tapichalaca Reserve), *Pristimantis* sp. (CCS1) (Yanuncay-Irquis Protected Forest), and *Pristimantis* sp. (UCS2) (Sangay National Park). Nonetheless, *P. balionotus*, *P. gloria*, *P. jimenezi*, *P. percultus*, *P. phoxocephalus*, *P. spinosus*, *P. teslai*, *P. torresi*, *P. verrucolatus*, *Pristimantis* sp. (CCS2), *Pristimantis* sp. (UCS1), and *Pristimantis* sp. (UCS3) occur in areas currently threatened by agriculture, cattle raising, dam construction, mining, logging and human settlement (Coloma et al. 2004; Rodríguez et al. 2004). All species of *Huicundomantis* have a restricted distribution range (maximum confirmed Extent of Occurrence is 4330 km^2^ for *P. atratus*). As a result, most species are either Data Deficient or threatened with extinction. The recognition of 13–16 new species of *Pristimantis* for central and southern Andes of Ecuador, all with small distribution ranges (up to 2388 km^2^ for the new species), provides new reasons for the conservation of this region.

## Supplementary Material

XML Treatment for Huicundomantis

XML Treatment for Pristimantis atillo

XML Treatment for Pristimantis chomskyi

XML Treatment for Pristimantis gloria

XML Treatment for Pristimantis jimenezi

XML Treatment for Pristimantis lutzae

XML Treatment for Pristimantis multicolor

XML Treatment for Pristimantis phoxocephalus

XML Treatment for Pristimantis teslai

XML Treatment for Pristimantis torresi

XML Treatment for Pristimantis totoroi

XML Treatment for Pristimantis verrucolatus

XML Treatment for Pristimantis nangaritza

## Supplementary materials

Supplementary material 1 Primers used for DNA PCRs Data type: molecular data Explanation note: Primers used for amplification of DNA. https://binary.pensoft.net/file/322432This dataset is made available under the Open Database License (http://opendatacommons.org/licenses/odbl/1.0/). The Open Database License (ODbL) is a license agreement intended to allow users to freely share, modify, and use this Dataset while maintaining this same freedom for others, provided that the original source and author(s) are credited.Nadia B. Páez, Santiago R. Ron

Supplementary material 2 Examined specimens Data type: occurrence Explanation note: Museum specimens analyzed in this taxonomic review. *: analyzed only with molecular information. ** = analyzed only with photographic material. https://binary.pensoft.net/file/322433This dataset is made available under the Open Database License (http://opendatacommons.org/licenses/odbl/1.0/). The Open Database License (ODbL) is a license agreement intended to allow users to freely share, modify, and use this Dataset while maintaining this same freedom for others, provided that the original source and author(s) are credited.Nadia B. Páez, Santiago R. Ron

Supplementary material 3 Phylogram of *Huicundomantis* including outgroup Data type: phylogram Explanation note: ML tree for genes 16S, ND1 and RAG1. Bootstrap values (%) are shown under the corresponding branches; missing values indicate values below 50 %. The number of collection, identification, country and locality of the samples are shown next to each terminal. EC is for Ecuador, PE for Peru, NP is for National Park, PF is for Protected Forest. Terminals pointed with an arrow correspond to specimens from Peru previously ascribed to *P. phoxocephalus*. Figure includes only outgroup within *Pristimantis*. See vouchers and GenBank accession numbers of all sequences used for molecular analyses in Table 1. https://binary.pensoft.net/file/322434This dataset is made available under the Open Database License (http://opendatacommons.org/licenses/odbl/1.0/). The Open Database License (ODbL) is a license agreement intended to allow users to freely share, modify, and use this Dataset while maintaining this same freedom for others, provided that the original source and author(s) are credited.Nadia B. Páez, Santiago R. Ron

Supplementary material 4 Mitochondrial phylogram depicting relationships within *Huicundomantis* Data type: phylogram Explanation note: ML tree for genes 16S and ND1. Bootstrap values (%) are shown under the corresponding branches; missing values indicate values below 50 %; asterisks indicate support values of 100%. The number of collection, identification, country and locality of the samples are shown next to each terminal. EC is for Ecuador, PE for Peru, PA is for Panama, NP is for National Park, PF is for Protected Forest. Outgroup is shown. https://binary.pensoft.net/file/322435This dataset is made available under the Open Database License (http://opendatacommons.org/licenses/odbl/1.0/). The Open Database License (ODbL) is a license agreement intended to allow users to freely share, modify, and use this Dataset while maintaining this same freedom for others, provided that the original source and author(s) are credited.Nadia B. Páez, Santiago R. Ron

Supplementary material 5 Phylogram depicting relationships within *Huicundomantis* Data type: phylogram Explanation note: ML tree for nuclear gene RAG1. Bootstrap values (%) are shown under the corresponding branches; missing values indicate values below 50 %. The number of collection, identification, country and locality of the samples are shown next to each terminal. EC is for Ecuador, PE for Peru, PA for Panama, NP is for National Park, PF is for Protected Forest. Outgroup is shown. https://binary.pensoft.net/file/322436This dataset is made available under the Open Database License (http://opendatacommons.org/licenses/odbl/1.0/). The Open Database License (ODbL) is a license agreement intended to allow users to freely share, modify, and use this Dataset while maintaining this same freedom for others, provided that the original source and author(s) are credited.Nadia B. Páez, Santiago R. Ron
