# Supplementary material for: Systematics of Huicundomantis, a new subgenus of Pristimantis (Anura, Strabomantidae) with extraordinary cryptic diversity and eleven new species
Source: Zookeys. 2019 Aug 1;868:1–112. doi: 10.3897/zookeys.868.26766 (PMC6687670; doi:10.3897/zookeys.868.26766)
Supplement: Supplementary material 1 [file zookeys-868-001-s001.pdf]

Appendix A. Genes and primers used in the study.

Gene	Primer	Sequence	Source
16S	16L19 (forward)	AATACCTAACGAACTTAGCGATAGCTGGTT	Henicke et. al. 2007
	16H36E (reverse)	AAGCTCCAWAGGGTCTTCTCGTC	Henicke et. al. 2007
ND1	16s-frog (forward)	TTACCCTRGGGATAACAGCGCAA	Wiens et. al. 2005
	tMet-frog (reverse)	TTGGGGTATGGGCCCAAAAGCT	Wiens et. al. 2005
RAG1	R182 (forward)	GCCATAACTGCTGGAGCATYAT	Henicke et. al. 2007
	R270 (reverse)	AGYAGATGTTGCCTGGGTCTTC	Henicke et. al. 2007
