# Supplementary material for: Systematics of Huicundomantis, a new subgenus of Pristimantis (Anura, Strabomantidae) with extraordinary cryptic diversity and eleven new species
Source: Zookeys. 2019 Aug 1;868:1–112. doi: 10.3897/zookeys.868.26766 (PMC6687670; doi:10.3897/zookeys.868.26766)
Supplement: Supplementary material 3 [file zookeys-868-001-s003.pdf]

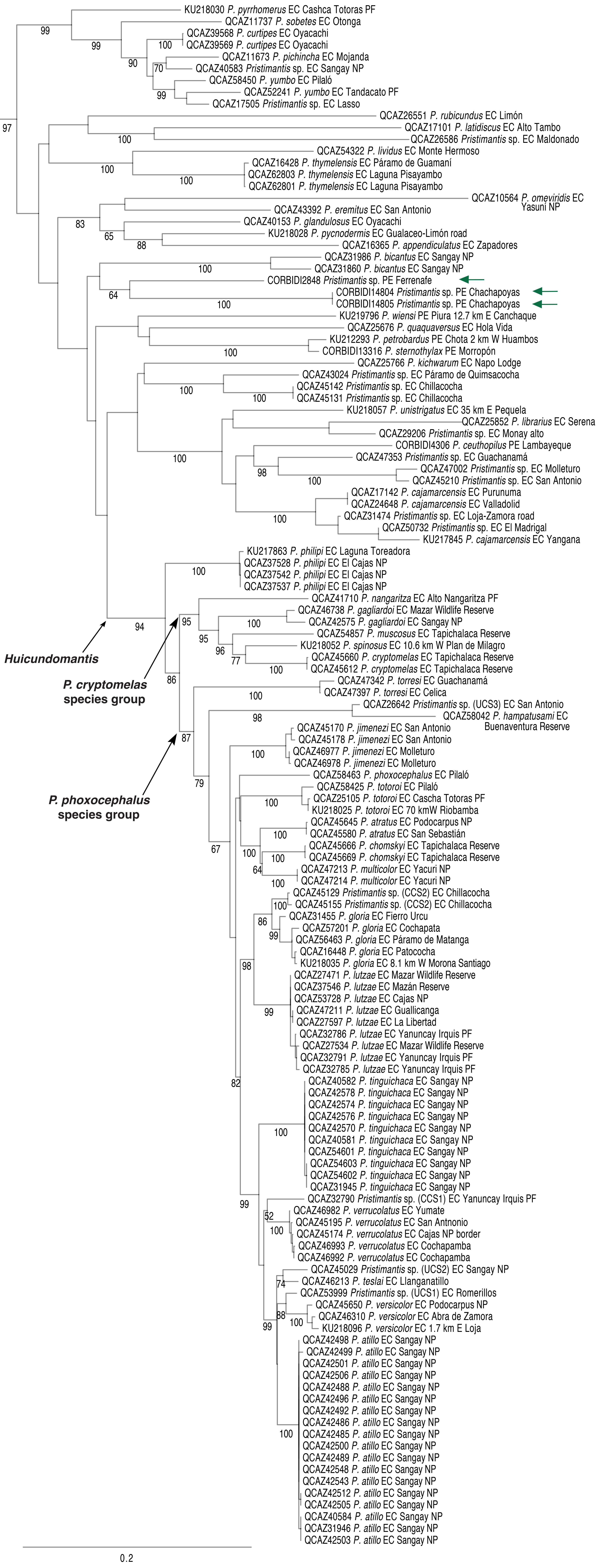

Appendix C. Phylogram of *Huicundomantis* including outgroup. ML tree for genes 16S, ND1 and RAG1. Bootstrap values (%) are shown under the corresponding branches; missing values indicate values below 50 %. The number of collection, identification, country and locality of the samples are shown next to each terminal. EC is for Ecuador, PE for Peru, NP is for National Park, PF is for Protected Forest. Terminals pointed with an arrow correspond to specimens from Peru previously ascribed to P. phoxocephalus. Figure includes only outgroup within *Pristimantis*. See vouchers and GenBank accession numbers of all sequences used for molecular analyses in Table 1.
