# Supplementary figures and images for: Systematics of Huicundomantis, a new subgenus of Pristimantis (Anura, Strabomantidae) with extraordinary cryptic diversity and eleven new species
Source: Zookeys. 2019 Aug 1;868:1–112. doi: 10.3897/zookeys.868.26766 (PMC6687670; doi:10.3897/zookeys.868.26766)

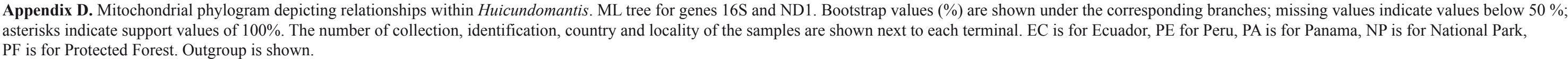

Supplement: Supplementary material 4 [file zookeys-868-001-s004.pdf]
