# Supplementary material for: Systematics of Huicundomantis, a new subgenus of Pristimantis (Anura, Strabomantidae) with extraordinary cryptic diversity and eleven new species
Source: Zookeys. 2019 Aug 1;868:1–112. doi: 10.3897/zookeys.868.26766 (PMC6687670; doi:10.3897/zookeys.868.26766)
Supplement: Supplementary material 5 [file zookeys-868-001-s005.pdf]

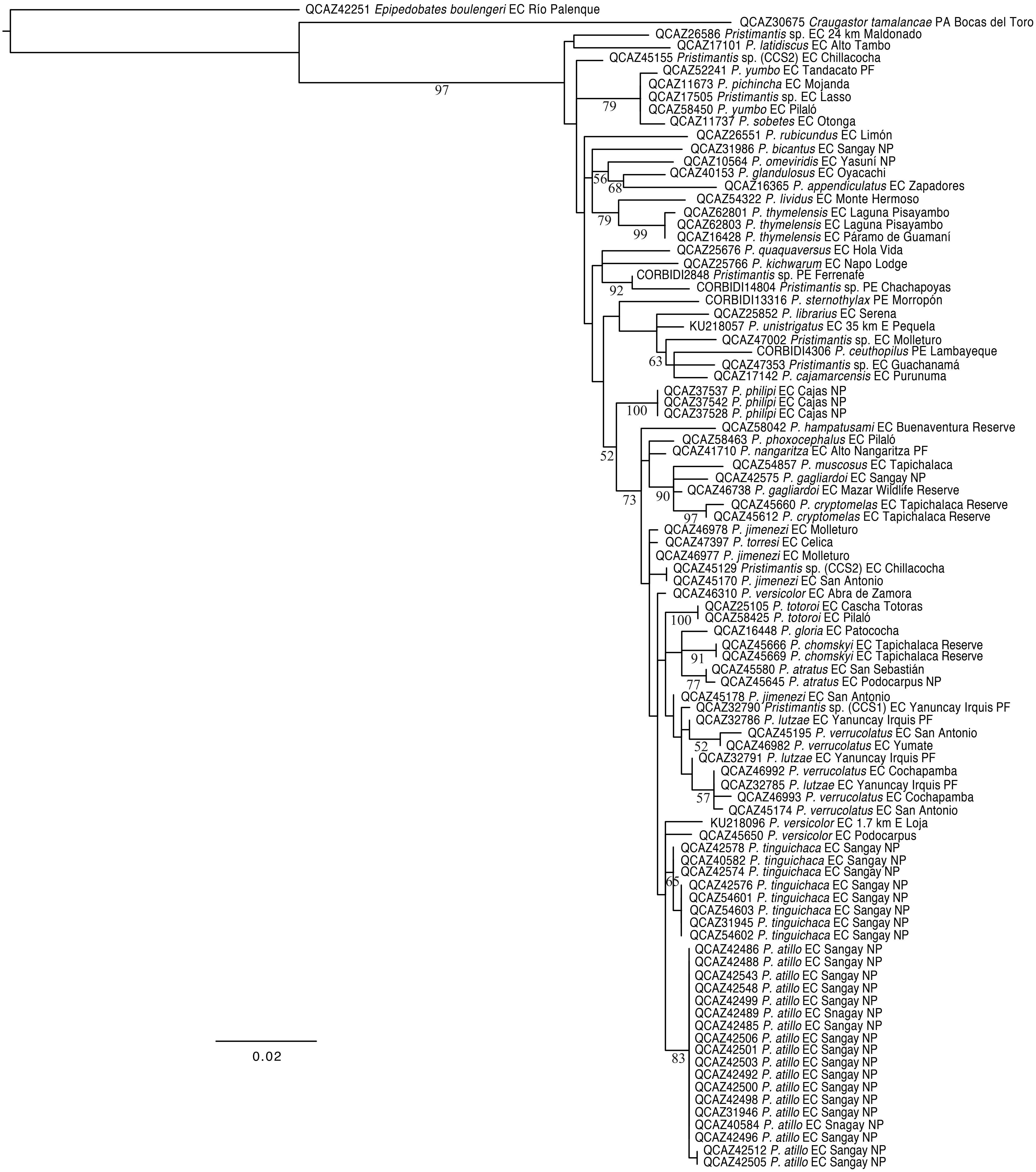

Appendix E. Phylogram depicting relationships within *Huicundomantis*. ML tree for nuclear gene RAG1. Bootstrap values (%) are shown under the corresponding branches; missing values indicate values below 50 %. The number of collection, identification, country and locality of the samples are shown next to each terminal. EC is for Ecuador, PE for Peru, PA for Panama, NP is for National Park, PF is for Protected Forest. Outgroup is shown.
